# Prioritizing natural-selection signals from the deep-sequencing genomic data suggests multi-variant adaptation in Tibetan highlanders

**DOI:** 10.1093/nsr/nwz108

**Published:** 2019-08-07

**Authors:** Lian Deng, Chao Zhang, Kai Yuan, Yang Gao, Yuwen Pan, Xueling Ge, Yaoxi He, Yuan Yuan, Yan Lu, Xiaoxi Zhang, Hao Chen, Haiyi Lou, Xiaoji Wang, Dongsheng Lu, Jiaojiao Liu, Lei Tian, Qidi Feng, Asifullah Khan, Yajun Yang, Zi-Bing Jin, Jian Yang, Fan Lu, Jia Qu, Longli Kang, Bing Su, Shuhua Xu

**Affiliations:** 1 Key Laboratory of Computational Biology, CAS-MPG Partner Institute for Computational Biology, Shanghai Institute of Nu-trition and Health, Shanghai Institutes for Biological Sciences, University of Chinese Academy of Sciences, Chinese Academy of Sciences, Shanghai 200031, China; 2 School of Life Science and Technology, ShanghaiTech University, Shanghai 201210, China; 3 State Key Laboratory of Genetic Resources and Evolution, Kunming Institute of Zoology, Chinese Academy of Sciences, Kunming 650223, China; 4 State Key Laboratory of Genetic Engineering and Ministry of Education (MOE) Key Laboratory of Contemporary Anthropology, School of Life Sciences, Fudan University, Shanghai 200433, China; 5 The Eye Hospital, School of Ophthalmology & Optometry, Wenzhou Medical University, China National Center for International Research in Regenerative Medicine and Neurogenetics, State Key Laboratory of Ophthalmology, Optometry and Visual Science, Wenzhou 325027, China; 6 Institute for Molecular Bioscience, Queensland Brain Institute, The University of Queensland, Brisbane, QLD 4072, Australia; 7 Key Laboratory for Molecular Genetic Mechanisms and Intervention Research on High Altitude Disease of Tibet Autonomous Region, School of Medicine, Xizang Minzu University, Xianyang 712082, China; 8 Center for Excellence in Animal Evolution and Genetics, Chinese Academy of Sciences, Kunming 650223, China; 9 Collaborative Innovation Center of Genetics and Development, Shanghai 200438, China; 10 Human Phenome Institute, Fudan University, Shanghai 201203, China

**Keywords:** Tibetan, adaptive genetic variant, high-altitude adaptation, next-generation sequencing (NGS), archaic ancestry, expression quantitative traits loci (eQTL), tissue-specific expression, hemoglobin concentration, hypoxia

## Abstract

Human genetic adaptation to high altitudes (>2500 m) has been extensively studied over the last few years, but few functional adaptive genetic variants have been identified, largely owing to the lack of deep-genome sequencing data available to previous studies. Here, we build a list of putative adaptive variants, including 63 missense, 7 loss-of-function, 1,298 evolutionarily conserved variants and 509 expression quantitative traits loci. Notably, the top signal of selection is located in *TMEM247*, a transmembrane protein-coding gene. The Tibetan version of *TMEM247* harbors one high-frequency (76.3%) missense variant, rs116983452 (c.248C > T; p.Ala83Val), with the T allele derived from archaic ancestry and carried by >94% of Tibetans but absent or in low frequencies (<3%) in non-Tibetan populations. The rs116983452-T is strongly and positively correlated with altitude and significantly associated with reduced hemoglobin concentration (*p* = 5.78 × 10^−5^), red blood cell count (*p* = 5.72 × 10^−7^) and hematocrit (*p* = 2.57 × 10^−6^). In particular, *TMEM247*-rs116983452 shows greater effect size and better predicts the phenotypic outcome than any *EPAS1* variants in association with adaptive traits in Tibetans. Modeling the interaction between *TMEM247*-rs116983452 and *EPAS1* variants indicates weak but statistically significant epistatic effects. Our results support that multiple variants may jointly deliver the fitness of the Tibetans on the plateau, where a complex model is needed to elucidate the adaptive evolution mechanism.

## INTRODUCTION

It is generally believed that long-term human inhabitation of the Tibetan highlands, where the oxygen pressure is much lower than at sea level (∼60%), is linked to a genetic adaptation to hypoxic environments [1]. Many genetic studies have been conducted to search for candidate loci associated with high-altitude adaptation (HAA) in Tibetans. The convergence of these studies strongly supports the crucial roles of two genes, *EPAS1* and

*EGLN1*, as members of the hypoxia-inducible transcription factor (HIF) pathway in the HAA of Tibetans [2–11]. A major undertaking of subsequent studies is to determine the functional genetic variants of the HAA candidate genes identified from previous genome-wide scans. One successful example is a high-frequency missense mutation in *EGLN1* contributing functionally to the Tibetans’ high-altitude phenotype *in vitro* [9–11]. However, most other attempts with the similar purpose of identifying functional variants in other genes, including *EPAS1*, have not been successful. Previous studies rely largely on the examination of some tagging single-nucleotide polymorphisms (SNPs) in individual candidate genes or SNP-array-based genome-wide scans. These strategies suffer from SNP ascertainment bias and thus possibly have less power to locate the functional variants [12,13], while only the whole-genome sequencing (WGS) offers near complete coverage of the genome, including non-coding regions [14]. More importantly, HAA involves a wide range of phenotypic variation. Like other complex traits, it is expected to be driven by enormously large numbers of variants spreading across the genome [15]. However, the lack of high-coverage WGS data in any single studies previously prevented the identification of functional variants associated with adaptive traits [9–11].

To obtain a comprehensive knowledge of the genome variation of Tibetan highlanders, and to gain further insights into the genetic bases of human adaptation to high altitudes, we complied a multi-omics dataset encompassing deep-sequenced genomes (30–60×) of 38 Tibetan highlanders (TIB) and 39 Han Chinese lowlanders (HAN) [16], RNA-Seq transcriptomes of 57 term placentas of Tibetans [17] and 62 quantitative traits in 2,849 Tibetan highlanders [18]. A systematic analysis of these data enabled us to search for known and novel candidate adaptive genetic variants (hereafter referred to as AGVs) on a whole-genome scale, while minimizing bias. These efforts are expected to facilitate further molecular-functional studies and provide a better understanding of the evolutionary mechanisms of human adaptation to life on the Himalayan plateau.

## RESULTS

### Candidate AGVs in Tibetan highlanders

We analysed 11.57 million biallelic single-nucleotide variants (SNVs) discovered in the deep-sequenced genomes, including 1.75 million (15.1%) novel SNVs not reported in dbSNP build 151 (Supplementary Table 1). Most of the SNVs (∼95%) act as modifiers in regulatory regions with mild impact, e.g. transcription factor binding variants, and are difficult to capture without whole-genome deep sequencing (Supplementary Table 2). The remaining 5% include 56,473 high-impact variants (3000 loss-of-function (LoF) variants and 53,473 missense variants) and 54,572 low-impact variants. By analysing the genetic variation within and between populations (see Methods), we identified 374 genomic regions with fine-mapped signals of positive selection. Of these regions, 254 contain at least one protein-coding gene and 66 regions do not overlap with any known genes (Supplementary Table 3).

**Figure 1.**
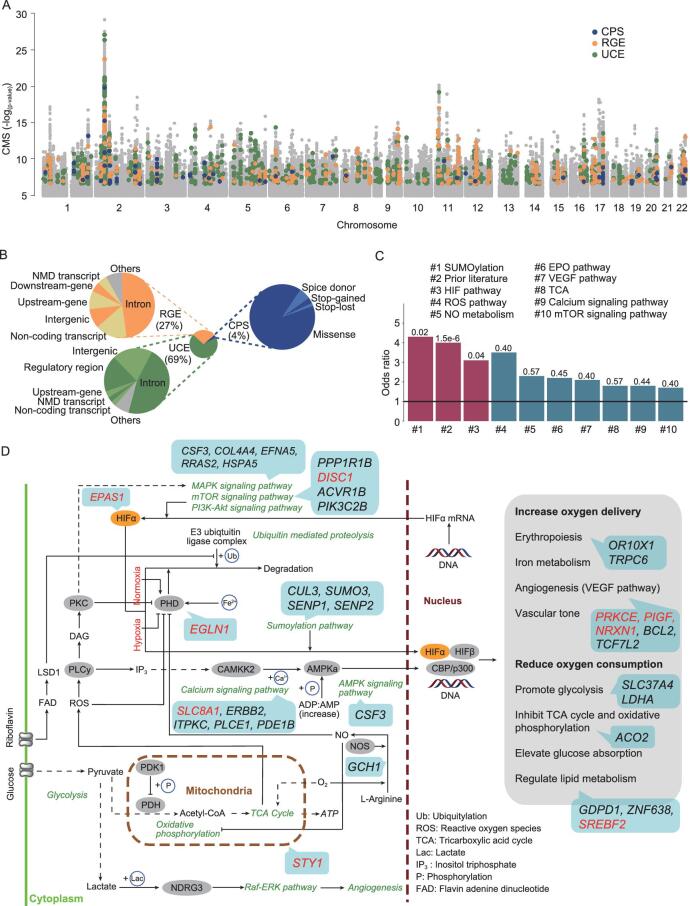
The landscape of the candidate AGVs in TIB and the candidate adaptive genes involved in the hypoxia-induced pathways. (A) Manhattan plot of the CMS scores across the autosomes. The candidate AGVs are labeled according to their biological effect. CPS, changing protein sequence; RGE, regulating gene expression; UCE, unknown function but conserved in evolution. (B) Proportions of different types of candidate AGVs. A majority of the candidate AGVs are located in the non-coding regions, making our analyses more comprehensive than those of previous studies. (C) The functional enrichment of AGV-related genes. The full priori gene list for each pathway or category appears in Supplementary Table 7. ‘Prior literature’ indicates genes reported by previous studies on high-altitude adaptation in human and non-human species. Here, we only show the 10 categories with odds ratios >1 (the *y*-axis). The horizontal line in black indicates odds ratio at 1. The adjusted *p* values for the enrichment of each category are shown above the bars. The red bars indicate significant enrichments (adjusted *p* < 0.05). (D) HIF pathways and related reactions under normoxia and hypoxia. Candidate adaptive genes (in the blue boxes) are mapped to the pathways they could possibly be involved in. Genes highlighted in red are suggested to carry genomic segments introgressed from archaic hominids (see Methods).

As the aim of this study is to identify candidate AGVs specific to Tibetan highlanders, we screened the above genomic regions and retained those showing considerable divergence between Tibetan and non-Tibetan populations (see Methods). To this end, we built a list of 1,877 candidate AGVs with at least one of the three categories of biological effects (see Methods, Fig. 1A and B, and Supplementary Table 4): changing protein sequence (CPS), including 1 stop-lost variant, 2 stop-gained variants, 4 splice-donor variants and 63 missense variants (Table 1 and Supplementary Table 5); regulating gene expression (RGE), including 509 expression quantitative traits loci (eQTLs); unknown function but conserved in evolution (UCE), including 1,297 variants with a combined annotation dependent depletion (CADD) [19] score >15 or a genomic evolutionary rate profiling (GERP) [20] score >2. These candidate AGVs fell into 521 genes (hereafter referred to as candidate adaptive genes; Supplementary Table 6) in 319 fine-mapped candidate regions. Only 21 candidate adaptive genes have been formally reported in previous HAA studies on either human or non-human species inhabiting the highlands in Tibet, Ethiopia or South America (Supplementary Table 6), suggesting that the vast majority of the genes and regions we identified are novel candidates of HAA.

### Potential functional and phenotypic effects of the candidate AGVs

Based on database and literature analyses, here we briefly summarize the potential functional and phenotypic effects of the candidate AGVs. There were three missense candidate AGVs (rs192690066, rs116983452 and rs12612916) identified in *TMEM247,* which encodes for transmembrane proteins and is located close to the well-studied *EPAS1.* In particular, rs116983452 had a larger composite multiple signal (CMS) score (19.85) over the other two missense loci, and it showed a greater genetic differentiation between TIB and HAN (*F*_ST_ = 0.72) than that of any other SNPs in *EPAS1* and *TMEM247*. The missense candidate AGV in *EGLN1*, rs186996510, is the only functional causal mutation identified in the Tibetan people in previous studies [6,7,9,10,15]. The candidate AGV rs5758511 is a stop-gain polymorphism in *CENPM*. The derived allele at this locus was reported to be associated with reduced birth weight in the Europeans [21] and it presents a higher frequency in TIB (0.76) than in HAN (0.46) in our data. We did not identify any candidate CPS-AGV in *EPAS1*, but found a downstream intergenic variant rs1900592 showing the largest CMS score (23.76) among the candidates. According to the Genotype-Tissue Expression (GTEx) database [22], rs1900592 is an eQTL that regulates the expression of *EPAS1* in blood. The region Chr11: 18344845–18479845 showed the second strongest CMS signal across the genome (Fig. 1A) and encompasses four candidate RGE-AGVs and two candidate UCE-AGVs. *LDHAL6A*, *LDHA* and *LDHC* belonging to the lactate dehydrogenase gene family are in this region and they are involved in anaerobic glycolysis. Other interesting candidate adaptive genes include *HLA-DMA* involved in immunity [23], *FRAS1* associated with renal agenesis [24], *SREBF2* related to female reproduction [25] and *DISC1* associated with response to the ultraviolet (UV) exposure [5,26].

### Enrichment of candidate AGVs in hypoxia-related pathways

As most of the candidate adaptive genes identified in our study are novel candidates of HAA and therefore could have not been well investigated, we used two intersecting approaches to infer the possible functional effects of them. First, we referred to genes involved in several hypoxia-related pathways because of their known functions. Second, we tested the associations between the candidate AGVs and phenotypes, as well as gene-expression levels in the Tibetans.

We collected genes involved in several hypoxia-related pathways defined by PathCards [27] and merged them with those that have been reported in previous HAA studies. The resulting set of 2,201 functional candidate genes is listed in Supplementary Table 7. We performed enrichment analysis (see Methods) and found that the 521 candidate adaptive genes were enriched in SUMOylation (odds ratio = 4.3, adjusted *p* = 0.01, Fisher’s exact test) and in the HIF pathway (odds ratio = 3.1, adjusted *p* = 0.03, Fisher’s exact test) (Fig. 1C). In addition, some genes appeared in the intersection of the candidate adaptive gene list and previous HAA studies (‘priori literatures’ in Supplementary Table 7), which was not likely to have occurred by chance (odds ratio = 4.0, adjusted *p* = 1.5 × 10^−6^, Fisher’s exact test).

**Table 1.** Candidate AGVs with missense or loss-of-function mutations^a^.

SNP	Gene		Nucleotide	Amino acid	*F*_ST_	*f*_TIB_	*f*_HAN_	SNP	Gene		Nucleotide	Amino acid	*F*_ST_	*f*_TIB_	*f*_HAN_
rs116983452	*TMEM247*		c.248C > T	p.Ala83Val	0.72	0.76	0.03	rs6740879	*CCDC138*		c.344G > A	p.Arg115Lys	0.16	0.22	0.03
rs186996510	*EGLN1*		c.12C > G	p.Asp4Glu	0.45	0.53	0.04	rs10008489	*FRAS1*		c.118 T > C	p.Ter40ArgextTer23	0.15	0.87	0.60
rs6679056	*OR10R2*		c.647A > G	p.Glu216Gly	0.24	0.84	0.49	rs6126344	*SALL4*		c.1520 T > G	p.Leu507Arg	0.15	0.62	0.32
rs1804020	*ZNF638*		c.1996G > A	p.Val666Met	0.23	0.87	0.53	rs1800517	*COL4A4*		c.3011C > T	p.Pro1004Leu	0.15	0.63	0.33
rs2838697	*SUMO3*		c.205G > T	p.Val69Phe	0.22	0.78	0.42	rs11062385	*KDM5A*		c.2594 T > C	p.Met865Thr	0.15	0.82	0.54
rs863363	*OR10X1*		c.179 T > C	p.Ile60Thr	0.21	0.84	0.51	rs2289247	*GNL3*		c.1063G > A	p.Val355Met	0.15	0.72	0.44
rs2228313	*SREBF2*		c.2580G > C	p.Arg860Ser	0.21	0.37	0.08	rs6617	*SPCS1*		c.121C > G	p.Pro41Ala	0.15	0.72	0.44
rs2075939	*NCF4*		c.815 T > C	p.Leu272Pro	0.20	0.93	0.65	rs1029871	*NEK4*		c.673C > G	p.Pro225Ala	0.15	0.72	0.44
rs4946188	*ZUFSP*		c.1135A > G	p.Asn379Asp	0.20	0.55	0.22	rs1459853	*GUCY1B3*		c.363 + 1G > A	NA	0.14	0.62	0.33
rs3827760	*EDAR*		c.1205 T > C	p.Val402Ala	0.20	0.32	0.05	rs1136410	*PARP1*		c.2285 T > C	p.Val762Ala	0.14	0.82	0.55
rs17029277	*RP11-766F14.2*		c.95G > A	p.Arg32Gln	0.20	0.91	0.62	rs3744093	*RNF43*		c.139A > G	p.Ile47Val	0.14	0.76	0.49
rs4689254	*ZBTB49*		c.1042G > A	p.Ala348Thr	0.20	0.88	0.58	rs292592	*WDR91*		c.770C > T	p.Pro257Leu	0.13	0.74	0.46
rs2271111	*DOCK5*		c.3068A > G	p.Gln1023Arg	0.19	0.88	0.59	rs2305925	*CATSPERD*		c.2227A > T	p.Thr743Ser	0.13	0.79	0.53
rs7105857	*COLCA2*		c.-285 + 2C > T	NA	0.18	0.80	0.49	rs6890099	*ATOX1*		c.-229 + 2 T > C	NA	0.13	0.63	0.36
rs2272843	*MOV10L1*		c.3536C > A	p.Ala1179Glu	0.18	0.42	0.13	rs2296129	*FAM209B*		c.386A > C	p.Glu129Ala	0.12	0.80	0.55
rs2272051	*DUSP11*		c.107A > G	p.Asp36Gly	0.18	0.58	0.26	rs4434138	*STAB1*		c.6844A > G	p.Ile2282Val	0.12	0.72	0.46
rs1877031	*STARD3*		c.350G > A	p.Arg117Gln	0.18	0.68	0.36	rs3745640	*PRR22*		c.353C > T	p.Pro118Leu	0.12	0.79	0.54
rs2295283	*MIIP*		c.499A > G	p.Lys167Glu	0.18	0.78	0.46	rs1048013	*CYP20A1*		c.1060C > T	p.Leu354Phe	0.12	0.87	0.64
rs2037814	*ALMS1*		c.2012 T > G	p.Val671Gly	0.18	0.51	0.21	rs2302190	*MTMR4*		c.838A > G	p.Ser280Gly	0.12	0.89	0.68
rs6762208	*SENP2*		c.872C > A	p.Thr291Lys	0.18	0.51	0.21	rs8073754	*SEPT4*		c.5G > A	p.Arg2Lys	0.12	0.89	0.68
rs8182086	*ZNF592*		c.2777G > A	p.Ser926Asn	0.18	0.76	0.45	rs12623638	*AC009965.2*		n.136 + 1G > A	NA	0.11	0.82	0.58
rs16853773	*PIK3C2B*		c.100C > T	p.Arg34Cys	0.18	0.82	0.51	rs4665385	*AC074091.13*		c.196G > A	p.Gly66Arg	0.11	0.80	0.56
rs3803650	*SLC7A6OS*		c.134G > A	p.Gly45Asp	0.17	0.53	0.22	rs1063478	*HLA-DMA*		c.211G > A	p.Val71Ile	0.11	0.91	0.71
rs12648093	*NUDT6*		c.340 T > C	p.Cys114Arg	0.17	0.21	0.01	rs3824915	*ALX4*		c.104G > C	p.Arg35Thr	0.10	0.74	0.50
rs10747561	*LMBR1L*		c.178G > A	p.Val60Ile	0.17	0.49	0.19	rs17773492	*LINC01118*		c.91A > G	p.Asn31Asp	0.09	0.93	0.77
rs7726005	*MGAT1*		c.668G > A	p.Arg223Gln	0.16	0.93	0.69	rs3865452	*ADCK4*		c.280A > G	p.Thr94Ala	0.08	0.68	0.46
rs5758511	*CENPM*		c.7C > T	p.Arg3Ter	0.16	0.76	0.46								

^a^The missense or loss-of-function (LoF, highlighted with underlined fonts) candidate AGVs showing the highest differentiation (*F*_ST_) between TIB and HAN. *f*_TIB_ and *f*_HAN_ denote the adaptive allele frequency in TIB and HAN, respectively. Genes coding for non-coding RNAs (e.g. miRNA and LincRNA) are underlined, as the missense or LoF effects in these genes are hard to confirm. Variants were annotated based on the Ensembl database version 90 using Variant Effect Predictor (VEP). NA, not applicable. A full list of 70 missense or LoF candidate AGVs is given in Supplementary Table 5.

To provide a more intuitive understanding of the roles that the candidate adaptive genes might play in HAA, we constructed a putative adaptive map of the hypoxia-related pathways for the Tibetan population, including the HIF pathway and its related pathways as listed in Supplementary Table 7 (see Methods, Fig. 1D). *EPAS1*, encoding HIF2α, is the central gene in the HIF pathway. We identified several candidate adaptive genes involved in the post-translational modifications of the HIFα proteins and they may strongly affect the stability and activity of HIFα. For instance, *EGLN1* encoding an oxygen-dependent hydroxylase-domain enzyme called prolyl hydroxylase 2 (PHD2) may induce the degradation of HIFα under normoxia [28–31]. The SUMOylation of HIFα in the nucleus also relies on *SENP1*, *SENP2*, *SUMO3* and *CUL3* [32–35]. Increasing the oxygen delivery and reducing the oxygen consumption are the two primary responses to hypoxia. The former relies on the improvement of blood and vascular conditions (e.g. erythropoiesis and angiogenesis) and seven candidate adaptive genes (*OR10X1*, *TRPC6*, *PRKCE*, *PIGF*, *NRXN1*, *BCL2* and *TCF7L2*) are related to this process; the latter mainly refers to the metabolism of glucose and lipids, in which *ACO2*, *SLC37A4*, *LDHA*, *GDPD1*, *ZNF638* and *SREBF2* may play crucial roles. We found that most of the genes presented in the pathway had significant interactions with *EP300* (histone acetyltransferase p300, *p* = 2.65 × 10^−4^; Supplementary Fig. 1). *EP300* is a co-activator of HIF1α [6,36,37] and it stimulates the hypoxia-induced genes, such as the vascular endothelial growth factor (VEGF) [37–39]. This gene has been reported to show signals of selection in the genome-wide comparisons between Tibetans and Han Chinese [6] and might contribute to HAA through regulating nitric oxide (NO) production in Tibetans according to a genetic-association test [40]. Taken together, these results emphasize the importance of post-translational modifications of *EPAS1* and indicate that the regulation of HIF-induced downstream pathways underlies the response to hypoxic conditions in Tibetans.

### Association of candidate AGVs with phenotypes in Tibetans

We next performed association studies of the candidate AGVs with 62 quantitative traits collected from 2,849 Tibetan samples (Supplementary Table 8) [18]. We applied a linear additive model and found that 73 candidate AGVs distributed in 17 genes were associated with at least one of these traits after correcting for multiple tests (Supplementary Table 9). Importantly, 61 of these candidate AGVs were located in seven continuous protein-coding genes on chromosome 2: *EPAS1*, *TMEM247*, *ATP6V1E2*, *RHOQ*, *PIGF*, *CRIPT* and *SOCS5*. The adaptive alleles at these loci showed strong associations with the reduced levels of red blood cell count (RBC, adjusted *p* = 3.10 × 10^−7^ – 0.045), hemoglobin (HGB, adjusted *p* = 2.90 × 10^−5^ – 0.045) and hematocrit (HCT, adjusted *p* = 1.21 × 10^−6^ – 0.046), which were proved to be adaptive traits of the Tibetan highlanders [2,5,41,42]. Moreover, except *RHOQ* and *PIGF*, the other five genes showed significant associations with uric acid (UA) level (top adjusted *p* = 1.89 × 10^−5^ – 0.024 for each gene)—a useful biomarker of vascular dysfunction (e.g. pulmonary hypertension) [43]. Our results also suggest that *MFN2* was significantly associated with folate (adjusted *p =* 0.035). The folate-increasing effect of the *MFN2* variant indicates the possibility of genetic compensation for the UV-induced folate degradation to support pregnancy and increase fertility at highlands [18]. It is also interesting that the *PPP1R1B* locus was associated with phosphorus. Phosphorus plays an important role in multiple biological processes, including oxidative phosphorylation, which is crucial for energy metabolism. *PPP1R1B* is associated with RBC and HGB in the populations with European ancestry [44], but these associations were not observed in the Tibetans studied here. It is notable that most of these associations (except that between rs1495099 in *PPP1R1B* and phosphorus) were confirmed when using an alternative approach—a mixed linear model-based leave one chromosome out association (MLMA-LOCO) analysis (see Methods; Supplementary Table 10). Additionally, several candidate AGVs were identified to be associated with the reduced height and the increased creatinine level in Tibetans using this approach. We found a weak association between *EGLN1* and HGB in the Tibetan males (*p* = 0.038 at rs186996510, but not significant after correcting for multiple test), consistently with previous findings [4,9,18].

### Association of candidate AGVs with gene expression in term placentas

Previous data showed that Tibetan women with high oxygen saturation have more surviving children than those with low oxygen saturation [45]. Gene expression in term placenta—a key organ for maternal–fetal oxygen exchange—may largely reflect the status and fitness of the fetus, but the data have never been reported by the GTEx Project [22]. Therefore, we performed a quantitative transcriptomics analysis to quantify the gene expression in 57 Tibetan term placentas. We tested the associations between the expression profiles of 592 candidate AGVs in the coding regions and 310 candidate adaptive genes, and identified 54 candidate AGVs that have *cis*-regulatory effects on 19 genes in term placenta (Supplementary Table 11 and Supplementary Fig. 2).

The eQTLs for each gene tended to be in extremely strong linkage (*r^2^* > 0.5 for pairwise SNPs located <100 kb from each other in the 57 Tibetan genotypes for the eQTL study; *r^2^* > 0.9 in the 2,849 Tibetan genotypes for the trait study and in the 38 Tibetan genomes), but with three exceptions including *EPAS1*, *TMEM247* and *CSF2RB* (Supplementary Fig. 3). The aforementioned missense variant rs116983452 was significantly associated with the expression of *EPAS1* (adjusted *p* = 0.003) and *TMEM247* (adjusted *p* = 0.038). According to our data, the expression level is high for *EPAS1* (in top 1% of the whole genome) but low for *TMEM247* in Tibetans’ placentas, and both of them were down-regulated by the adaptive allele (T, derived allele) at rs116983452. Consistently, Peng *et al.* [17] also reported the down-regulation of *EPAS1* transcription in placentas in the Tibetans. However, we could not determine the causality of this variant to the gene expression, as it is in strong linkage with several intronic eQTLs located in *TMEM247* (rs1868079, *r^2^* = 0.92; rs116871724, *r^2^* = 0.92; rs79542054, *r^2^* = 0.88), which is also the case for some other missense candidates showing substantial correlation with the gene expression (Supplementary Fig. 3). The missense variant rs3745640, which is also the top CMS signal in *PRR22*, was identified as an eQTL of *DUS3L*. It is in strong linkage with the synonymous rs10811 in *DUS3L* (*r^2^* = 0.78). The most significant eQTL of *SEPT3*, rs2228313, is a missense candidate AGV in *SREBF2*. It is involved in the HIF pathway (Fig. 1D) and is in complete linkage with rs17848337 (*r^2^* = 1) in *SEPT3*. Interestingly, the four eQTLs of *GNL3* are all missense variants in an linkage disequilibrium (LD) block (pairwise *r^2^* = 0.88–1), in which rs11177 and rs2289247 are the top two CMS signals in *GNL3*, while rs6617 and rs1029871 are candidate AGVs identified in *SPCS1* and *NEK4*, respectively. Different roles that a variant could play in different genes also increased the difficulty to unravel the genetic basis of HAA. For instance, rs3865452 acts as a missense candidate AGV in *ADCK4*, but was identified to be the only eQTL of *RAB4B*, which is 60 kb downstream from *ADCK4*. *RAB4B* encodes a protein that is involved in vesicular trafficking [46] and is an important paralogue of *EGLN2*; *EGLN2* is a HIF that plays an essential role in the response to hypoxia.

### Colocalization of eQTLs and phenotype-associated signals

**Figure 2.**
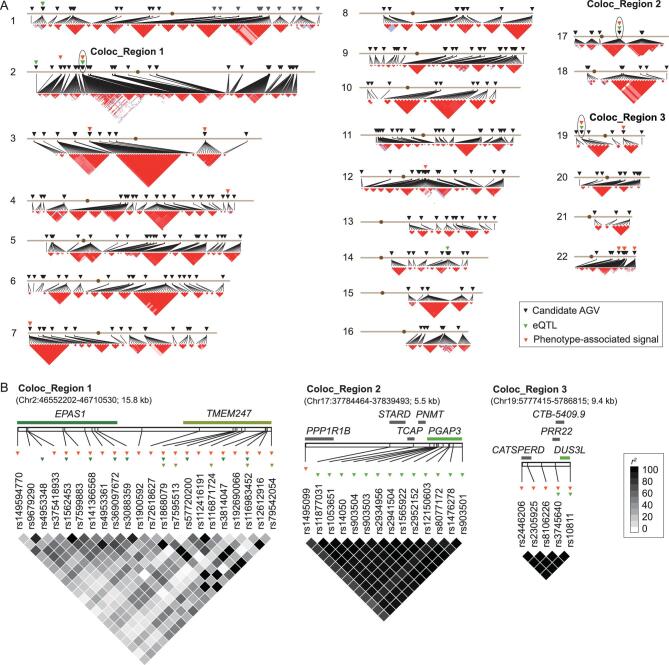
Colocalization of eQTLs and phenotype-associated candidate AGVs. (A) Genome-wide distribution and linkage disequilibrium (LD) of the candidate AGVs. The candidate AGVs, eQTLs and phenotype-associated loci are indicated by inverted triangles in black, green and orange, respectively. The LD blocks were inferred using Haploview version 4.2 [104] and are presented using the standard color scheme. The three regions of colocalization are marked using ellipses and are labeled as ‘Coloc_Region 1’, ‘Coloc_Region 2’ and ‘Coloc_Region 3’, respectively. (B) Zoom-in plots of candidate AGVs in the three colocalization regions. In each plot, gene locations are shown above the chromosome. The *cis*-regulated genes are indicated by green bars, while others are indicated by gray bars. The eQTLs and phenotype-associated loci are indicated by inverted triangles in green and orange, respectively. In Coloc_Region 1, the color of each inverted triangle for the eQTL matches that of the bar for the gene regulated by this eQTL. The LD of pairwise SNPs was measured by *r^2^* using Haploview version 4.2 [104].

Some eQTLs are colocalized with phenotype-associated signals in three regions of the Tibetan genomes (Fig. 2). For instance, most of the eQTLs in *EPAS1* and *TMEM247* are exactly matched with the association signals of UA, RGB, RBC and HCT; the eQTLs of *PGAP3* are only 3 kb downstream from a phosphorous-associated locus in *PPP1R1B*;

the *DUS3L* eQTLs were significantly associated with the gamma-glutamyl transpeptidase (GGT) level (Fig. 2 and Supplementary Table 9). In the latter two regions, the eQTLs and phenotype-associated loci are almost in complete LD. We further asked whether it is a coincidence or a causal relationship that leads to such colocalization. Using a stepwise regression approach implemented in the R package *coloc* version 3.1 [47], we tested the jointly estimated coefficients of the significantly associated candidate AGVs for each trait as mentioned above and those for gene expression (see Methods). The results presented in Supplementary Table 12 suggest that the expression of *EPAS1* and *TMEM247* is likely to be responsible for the variation of UA, RGB, RBC and HCT (adjusted *p* > 0.05 for all tested loci) and so is the *DUS3L* expression for GGT (adjusted *p* > 0.05 for all tested loci). We are not able to test the colocalization of signals in the *PGAP3* region, as only one phenotype-associated locus was identified but *coloc* considers two loci for each trait.

### Tissue-specific expression patterns of candidate HAA-related genes

**Figure 3.**
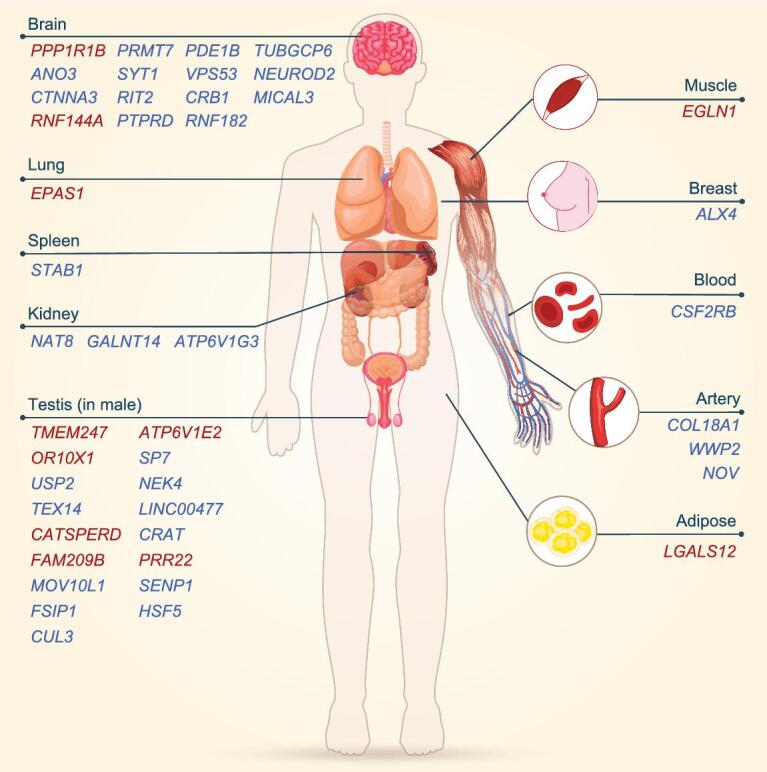
A human-anatomy plot showing tissue- and organ-specific expression of the candidate HAA-related genes. The expression profiles for these genes were obtained from the Genotype-Tissue Expression (GTEx) database. All reported tissues and organs are shown, except for the cell lines. Genes reported to be HAA-related in Tibetans or showing significant associations with phenotypes in our analysis of 2,849 Tibetans are highlighted in red, while the others are in blue. A full list of the expression patterns of all candidate HAA-related genes is given in Supplementary Table 6. The human-anatomy image was constructed at pngtree.com.

Based on our literature searches and data analysis, we selected 157 genes with potential functional relations with HAA from the candidate adaptive gene list and they were thus defined as candidate HAA-related genes (see Methods; Supplementary Table 6). We examined the expression profiles of the candidate HAA-related genes in GTEx and observed that 51 of them exhibited tissue-specific expression patterns in 11 tissue types (see Methods; Fig. 3). Here, the tissue-specific expression is determined following the GTEx Project [48] or defined as an observable higher expression level of a gene in a tissue or organ than in any others—in detail the median expression level of this gene in this tissue should be at least twice that in any other tissues and the lower quartile in this tissue should also be higher than the upper quartile of that in all the other tissues.

The brain, which controls neural activity, is the most oxygen-dependent organ in the body. The acute hypoxia experienced at extremely high altitudes may give rise to severe neuropsychological outcomes, like loss of consciousness and transient ischemia [49]. *PPP1R1B* was the most strongly up-regulated gene in the brain. As discussed above, we identified a candidate AGV related to phosphorous in *PPP1R1B*. *ANO3* is expressed in several regions of the human brain, particularly the putamen [50]. The angiogenic pathway in the putamen might be activated by hypoxia, based on an experimental study in rats [51]. Testes are male reproductive organs. Interestingly, sperm quality and quantity and testosterone levels are equivalent in men inhabiting high and low altitudes [49]; this equivalence might be the result of HAA. Two genes with outstanding signatures of natural selection, *TMEM247* and *ATP6V1E2*, were specifically expressed in the testes, although at low levels. The biological function of *TMEM247* is unclear; the protein encoded by *ATP6V1E2* is a subunit of a sperm-specific V-ATPase that is expressed in acidic secretory acrosomes essential for fertilization [52]. Another signal is *CATSPERD*. Proteins coded by this gene were detected in spermatocytes and spermatids at different stages of spermatogenesis in mice [53]. In addition, *EPAS1* and *EGLN1* are specifically expressed in the lungs and the muscles, respectively. *NOV*, a blood-pressure-associated gene [54], is specifically expressed in the arteries. The arteries are crucial for the maintenance of sufficient blood flow and thus influence blood oxygen. Spleen is the primary erythropoietic organ producing RBCs [55] and one candidate HAA-related gene *STAB1* is specifically expressed in the spleen. This gene was reported to be significantly associated with HCT level [44,56] and was down-regulated in response to hypoxia. Therefore, our results suggest that HAA is a complex biological process involving multiple organs and tissues.

### Prioritization of the candidate adaptive genes

**Figure 4.**
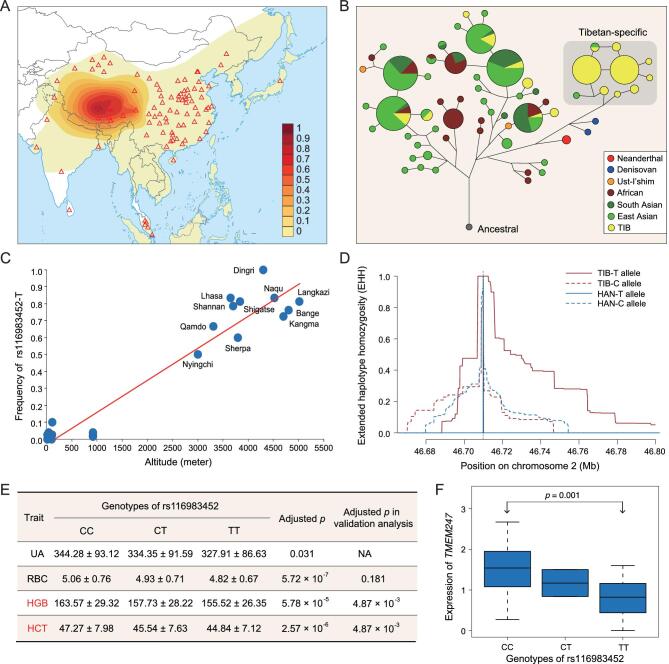
Signature of local adaptation at rs116983452 and its functional associations. (A) Global distribution of rs116983452-T. Each triangle represents a sampling locality for a population. The map is adapted from http://bzdt.ch.mnr.gov.cn (GS(2016)1665, approved by the Ministry of National Resources of the People’s Republic of China). (B) Median-joining network for *TMEM247*. The gray area highlights a group of Tibetan-enriched haplotypes with Denisovan origin, all of which carry rs116983452-T. (C) Correlation between the altitude and the derived allele frequency at rs116983452. Each dot represents an Asian population, from both public datasets and our unpublished data. Populations analysed in this plot include various Tibetan populations (labeled), as well as Uyghur, Tajik, Kazak, Hui, Han Chinese, Japanese and Malaysian peoples (unlabeled). (D) Estimation of extended haplotype diversity (EHH) in TIB and HAN around rs116983452. (E) Significant associations between rs116983452 and various quantitative traits. Associations validated in a larger Tibetan population are indicated with fonts in red. (F) The expression of *TMEM247* in three groups of Tibetan samples with different genotypes.

We further prioritized the candidate adaptive genes according to *FIS* (the functional importance score), which is a combined statistic of population differentiation and molecular functionality (see Methods; Supplementary Table 6). Strikingly, we found *TMEM247*, which has been poorly studied previously, appeared on the top of the list attributing to the key missense variant rs116983452 (c.248C > T; p.Ala83Val). The adaptive derived allele (T allele) at this locus is enriched in TIB (76.3%), while it is absent in African, European and American populations, and is in low frequencies in other East Asian populations (<3%) according to the 1000 Genomes dataset (see Fig. 4A for the contour density plots of rs116983452-T frequency). It is, to date, the most-differentiated functional variant identified between Tibetan and non-Tibetan populations. In fact, the genomic region that includes this variant is extremely divergent between TIB and HAN (maximum *F*_ST_ = 0.804) (Supplementary Fig. 4), in sharp contrast to the genome-wide average (*F*_ST_ = 0.015). The Tibetan-enriched allele was derived from archaic ancestry (Fig. 4B). It is strongly and positively correlated with altitude (*r* = 0.838, *p* value = 0.018) (Fig. 4C and Supplementary Fig. 5) and had a pronounced signature of nature selection (Fig. 4D). The selection coefficient estimated for rs116983452-T (*s* = 0.0035–0.0058) is higher than that estimated for the well-known missense variant (rs186996510) of *EGLN1* (*s* = 0.0024–0.004), although both are greater than that of most genome-wide candidate AGVs (median *s* = 0.001–0.0016) (Supplementary Table 4 and Supplementary Table 14), assuming that all these candidate AGVs share one selection event, which occurred after the split of the highlanders (TIB) and the lowlanders (HAN).

The candidate gene-association analysis showed that rs116983452-T was significantly associated with UA (adjusted *p* = 0.031), HGB (adjusted *p* = 5.78 × 10^−5^), RBC (adjusted *p* = 5.72 × 10^−7^) and HCT (adjusted *p* = 2.57 × 10^−6^), and it had substantial influence on *TMEM247* expression (Fig. 4E and F). Using 1,160 replication samples collected from four different altitudes, we validated the strong association between rs116983452-T and both altitude and the aforementioned hypoxia-related traits, e.g. HGB (adjusted *p* = 4.87 × 10^−3^) and HCT (adjusted *p* = 4.87 × 10^−3^) (Fig. 4E and Supplementary Table 15).

### Differentiation of selection and association between *TMEM247* and *EPAS1*

**Figure 5.**
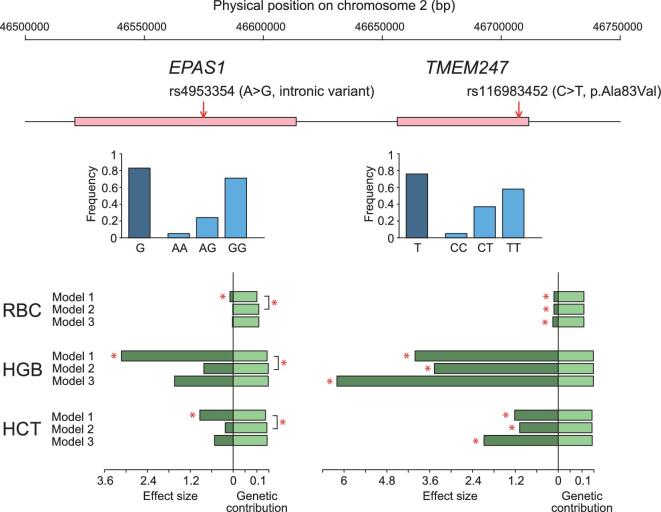
Effects of *EPAS1*-rs4953354 and *TMEM247*-rs116983452 on the adaptive traits in Tibetans. The intronic SNP rs4953354 reported in Beall *et al.* in 2010 [2] was selected as a representative adaptive *EPAS1* variant in comparison with the rs116983452 in *TMEM247*. The locations on chromosome 2 of the two genes are presented by the pink bars against the coordinates above and the positions of the two SNPs are indicated by arrows. For each SNP, the frequency of the adaptive allele and that of three genotypes are shown by blue bars. The genetic effects of rs4953354 and rs116983452 on red blood cell count (RBC), hemoglobin (HGB) and hematocrit (HCT) were tested using three linear-regression models, as illustrated in Methods. The effect size of each variant and the genetic contribution of each model are shown in the green bars below. Significant *p* values (*p* < 0.05) are denoted with asterisks. In Model 3, the effect size of the interaction of the two variants is not significant (*p* > 0.05) and thus is not shown in the figure. Detailed results can be found in Supplementary Table 13.

The adjacent physical locations (∼40 kb in distance) of *TMEM247* and *EPAS1* on the same chromosome raised the concern of a hitch-hiking effect, i.e. the observed signals at these genes might be correlated due to the LD. However, when examining the LD patterns of *TMEM247* and *EPAS1*, we found that they are located in two different LD blocks, separated by a strong recombination hotspot (Supplementary Fig. 6A). The correlations among the key candidate AGVs (e.g. rs1900592 in *EPAS1*, rs192690066, rs116983452 and rs12612916 in *TMEM247*) and other reported candidates (e.g. the 5-SNP-motif with Denisovan ancestry, the Tibetan-enriched deletion and several other important SNPs [8,11–13]) in each gene is smaller than those between *TMEM247* and *EPAS1* (Supplementary Fig. 6B). These results might suggest a much more complex mechanism of HAA in this region: the coexistence of candidate AGVs regulating gene expression and those altering protein sequences. We then statistically evaluated the individual and joint contributions of the multiple variants in the two genes, i.e. *EPAS1* and *TMEM247*, to the variation of adaptive phenotypes (RBC, HGB and HCT) in Tibetans using three models: (i) a simple linear-regression model considering either an *EPAS1* variant or a *TMEM247* variant, (ii) a binary linear-regression model considering both an *EPAS1* variant and a *TMEM247* variant and (iii) a binary linear-regression model considering an additional interaction term (see Methods). Taking rs4953354 reported in Beall *et al.* [2] as a representative candidate in *EPAS1*, we found *TMEM247*-rs116983452 explained a higher proportion of heritability of the phenotypes in Tibetans than the *EPAS1* variant (effect size in the Model 1: −0.12 vs. –0.09 for RBC; −4.01 vs. –3.13 for HGB; −1.22 vs. –0.93 for HCT) (Fig. 5 and Supplementary Table 13). The *TMEM247*-rs116983452 variant masked the effects of *EPAS1*-rs4953354 (*p* of the two loci in Model 2: 1.27 × 10^−6^ vs. 0.71 for RBC; 1.68 × 10^−4^ vs. 0.37 for HGB; 1.77 × 10^−5^ vs. 0.39 for HCT) and improved the fit of the model (genetic contributions of Model 1 (*EPAS1*-rs4953354) and Model 2: 0.100 vs. 0.108, *p* = 1.27 × 10^−6^ for RBC; 0.143 vs. 0.148, *p* = 1.68 × 10^−4^ for HGB; 0.136 vs. 0.142, *p* = 1.77 × 10^−5^ for HCT). Similar results were observed in the comparison between *TMEM247*-rs116983452 and most of the other adaptive variants in *EPAS1* (Supplementary Table 13). The epistatic interaction of *TMEM247* and *EPAS1* seems to be weak but statistically significant, in line with the loose correlation of the two genes indicated by the aforementioned LD pattern. Further investigation of these mechanisms will depend on the designation of many gene-expression and functional assays that separate the individual or joint contributions of each candidate AGV, which, though, is expected to be labor-intensive and time-consuming.

### Distinct ancestral architectures between *EGLN1* and *TMEM247*

*EGLN1* and *TMEM247* are the only two genes that harbor high-frequency missense functional candidate AGVs in the Tibetan highlanders. We therefore postulate that these two genes are functionally important and associated with the altitudinal adaptation of the Tibetan highlanders. It is noteworthy that archaic ancestry is completely absent from the entire *EGLN1* gene region in both TIB and HAN (see Methods; Supplementary Fig. 7A). We estimated that the time to the most recent common ancestor (TMRCA) of the haplotypes carrying the key *EGLN1* locus (rs186996510) [9,10] was 29,800 years (see Methods), which predates the Last Glacial Maximum (LGM)—a period of intense cold from ∼ 26,500 to 19,000 years before present (YBP) [57]. In contrast, elevated archaic ancestries in the Tibetans were observed in *TMEM247* (Supplementary Fig. 7B). The unusually high frequency of archaic sequences and substantial differences between TIB and HAN—as well as the other populations—could not be explained by recent gene flow or random processes. This indicated that *TMEM247* has been subjected to strong natural selection and likely contributes to the altitudinal adaptation of Tibetan highlanders. The surviving archaic sequences in *TMEM247* in Tibetan highlanders could be dated back to ∼ 60,000 YBP, again pre-dating the LGM [16]. Assuming that the selection of rs116983452-T in *TMEM247* occurred right after the archaic introgression, we estimated that the selection coefficient at this locus was 0.013–0.033 (Supplementary Table 14). The distinct and complicated ancestral architectures of *EGLN1* and *TMEM247* have many implications for Tibetan origins and their history in adapting to the plateau. Our results indicate that both archaic and modern human ancestries contribute to the HAA of the Tibetan highlanders and that human adaptation to high altitudes in Tibet is much more ancient than previously believed, as the key candidate AGVs facilitating the altitudinal adaptation of the Tibetan highlanders were likely derived from pre-LGM populations.

## DISCUSSION

As human migrations to the Tibetan plateau are likely a series of ‘stochastic adventures’ rather than well-planned expeditions, a wide range of phenotypic variations driven by enormously large numbers of variants and genes spreading across the genome are expected to have been instrumental in human adaptation to the plateau [15]. The convergence of previous studies supports the roles of two genes that are part of the HIF pathway, *EPAS1* and *EGLN1*, in the HAA of Tibetans [2–8]. However, our study provides a more comprehensive and prioritized list of candidate AGVs, of which only a few have been reported. It would facilitate further molecular-functional studies of HAAs and improve our understanding of human adaptation to the Himalayan plateau.

We analysed the same data—the whole-genome SNPs of 2,849 Tibetan samples—as Yang *et al.* [18] did, but used different strategies and criteria for the purpose of this study. Of the nine adaptive genes reported by Yang *et al.* [18], three (*EPAS1*, *EGLN1* and *NEK7*) were identified as candidates in our study. We realized that the candidate loci in the other six genes showed minor genetic differentiations between Tibetan and Han Chinese (Table 1 in Yang *et al.* [18]) and thus were not considered as candidates according to our criteria for selecting candidate AGVs. It should also be noted that *TMEM247*, rather than *EPAS1*, presented as the top signal in our study, inconsistently with previous findings [58–60]. Despite *EPAS1* variants also showing significant signals of positive selection (e.g. top *F*_ST_ = 0.8), when the biological effects (e.g. variant type and conservation) were taken into account, the top signals in *EPAS1* (including rs372272284 reported by Jeong *et al.* [59]) were filtered out due to the mild biological impact and the signals in *TMEM247* (e.g. rs116983452) survived. We believe it is essential to consider both statistical signals and biological effects of variants in the identification of candidate AGVs, as they are more likely to be the adaptive genetic variants.

Functional investigations will hopefully resolve which candidate AGVs are causal for adaptation and how these candidate AGVs have contributed to the altitudinal adaptations of the Tibetan highlanders. However, the challenges are obvious. For example, determining the causality and consequence of altitudinal adaptation is difficult, with many candidate AGVs discovered from whole-genome data. In the present case of *TMEM247*, which is located in a region encompassing seven genes and many genetic variants, it is difficult and complex to determine which are the ‘drivers’ and which are ‘passengers’. For instance, the causality or independency of *EPAS1* and *TMEM247* to the phenotypes in Tibetans could not be exclusively determined by the cross-conditional association analyses, although they are in different LD blocks (Supplementary Table 16). Therefore, we give higher weights to variants that are highly differentiated between populations with elevated archaic ancestry and strong association with the adaptive traits, such as the Tibetan-enriched missense rs116983452-T located in *TMEM247.*

Withstanding the challenging environmental conditions of the Tibetan highlands must have been a very long evolutionary process, possibly even longer than the history of most of modern Eurasian populations. This has been well illustrated by dating ages of candidate AGVs in the two outstanding genes (*EGLN1* and *TMEM247*) with distinct ancestry make-up. In the Tibetan genome, the entire *EGLN1* gene derived its ancestry exclusively from modern human groups (Supplementary Fig. 7A), while the *TMEM247* variants derived their ancestry mostly from archaic groups (Supplementary Fig. 7B). Therefore, it is evident that both anatomically modern humans (AMH) and non-AMH ancestries contributed to the HAA of the Tibetan highlanders. However, the key candidate AGVs as identified in the two outstanding genes (*EGLN1* and *TMEM247*) with distinct ancestries, either AMH- or non-AMH-originated, traced their origins back to ∼30,000 years ago and thus could be derived dominantly from pre-LGM populations, indicating ancient adaptation of humans to the Himalayan plateau.

Our data and analysis also suggest latecomers to the plateau might have typically inherited candidate AGVs from predecessors via genetic admixture, rather than via the creation of one or more candidate AGVs *de novo*. Supporting this hypothesis, most of the candidate AGVs identified in this study were standing variants, while hard sweeps are rare. Although founder effects could mimic positive selection on standing variants, we do not think that would be the case in our study, as it would influence the whole genome, rather than a single locus. Indeed, selection on standing variants may be common in human adaptation to local environments, as ‘archaic adaptive introgression’ has been suggested by many recent studies [11,61–66]. Knowing ancestral origins of the candidate AGVs is imperative, being aware of genetic continuity of early highland-foragers and present-day Tibetans [16] and understanding ‘borrowed fitness’ as a driving force of adaptive evolution is helpful for further investigations of the genetic mechanism of human adaptation to local environments.

Our results suggest that human HAA, associated with a wide range of complex traits, is driven by enormously large numbers of variants spreading across the genome, of which only a few have been identified. Moreover, even considering a single certain adaptive trait, we argue that more than one variant may jointly deliver the fitness even in a closely linked genomic region, suggesting the epistatic effect or genetic interaction has to be considered carefully. However, detecting interactions among variables is a well-known challenge in statistics and data mining [67]. For example, a complete model that includes all variants and all interaction terms may require too many degrees of freedom and is thus not feasible. For this reason, interactions are only tested for *TMEM247*-rs116983452 and those *EPAS1* variants that have a statistically significant independent main effect. Those DNA sequence variations that have an interaction effect, but no or minimal main effect, could have been missed. Modeling multi-variant adaptation is not the currency of human genetics and evolution, but may open a window to understanding human adaptation to high altitude.

## METHODS

### Samples and WGS

Peripheral blood samples were collected from 33 Tibetan and 5 Sherpa individuals living in six prefectures (Lhasa, Chamdo, Nagqu, Nyingchi, Shannan and Shigatse) in the Tibet Autonomous Region, and blood samples of 39 Han Chinese individuals were collected from diverse regions in China. Each individual was third (or more)-generation offspring of non-consanguineous marriages of members of the same nationality. All samples were collected with informed consent and approved by the Biomedical Research Ethics Committee of Shanghai Institutes for Biological Sciences (Shanghai, China). Prior to sequencing and analysis, all samples were stripped of personal identifiers (if any existed). All procedures were in accordance with the ethical standards of the Responsible Committee on Human Experimentation and the Helsinki Declaration of 1975, as revised in 2000. Briefly, WGS with a target high coverage (30–60×) was performed on Illumina HiSeq X Ten following Illumina-provided protocols with standard library preparation in WuXi NextCODE at Shanghai. Details regarding sample collection and genome sequencing have previously been described [16]. The raw data can be downloaded from National Omics Data Encyclopedia (NODE, http://www.biosino.org/node; accession number: ND00000013EP) or BIG Data Center (http://bigd.big.ac.cn/; accession number: PRJCA000246). Variant calling was carried out with the HaplotypeCaller module in the Genome Analysis Toolkit (GATK) [68,69] on a combined sample set, including 77 samples from this study and 182 additional unpublished deep-sequenced samples from diverse Asian populations. Then, data filtration was carried out in each single population using VCFtools (https://vcftools.github.io/index.html) [70] by removing SNVs that significantly deviated from the Hardy-Weinberg equilibrium (*p* < 10^−6^) or with a missing rate of more than 20%. Finally, 11.43 million SNVs were retained for further analyses.

### Genotype imputation and haplotype phasing

Haplotypes of 22 autosomes of 77 Tibetan and Han Chinese genomes were inferred using SHAPEIT version 2.r837 (https://mathgen.stats.ox.ac.uk/genetics_software/shapeit/shapeit.html) [71], together with the other 182 Asian samples mentioned above. No reference population was used in haplotype phasing, as it would substantially reduce the marker density, especially for the isolated highlander population. Then we applied a sample-independent mask to remove regions with low mappability or low complexity where variant calling can be challenging, following the Simons Genome Diversity Project [72].

The SNP-array data of 2,849 Tibetans were obtained from https://www.wmubiobank.org [18] and were imputed with a pipeline suggested by IMPUTE2 (http://mathgen.stats.ox.ac.uk/impute/impute_v2.html) [73]. First, the genotypes of 526,123 SNPs were phased with SHAPEIT version 2.r837 [71]. Then, for each 5 Mb non-overlapping genome segment, genotypes were imputed by IMPUTE2 [73], using 1,025 deep-sequenced whole genomes of diverse Asian populations (unpublished) as a reference panel. Consequently, 29,411,284 SNPs were obtained for this dataset.

Transcriptomic variants of 57 Tibetan placenta tissue samples were downloaded from the BIG Data Center (http://bigd.big.ac.cn/; accession numbers: PRJCA000268 and PRJCA000269) [17]. Genotypes were called using the GATK pipeline [74]. Because calling SNPs from RNA-Seq data tends to underestimate the proportion of heterozygotes, we counted the reads for reference and alternative alleles to adjust the possible underestimation [75]. Using this method, we obtained the genotype information of 1,528,173 SNPs in the coding regions.

### Genomic annotation of SNVs

The ancestral allele of each SNV was determined based on the ancestral sequences released by the 1000 Genomes Project. Genetic variant types were provided by Variant Effect Predictor (VEP, http://www.ensembl.org/info/docs/tools/vep/index.html) [76], which assigns each genetic variant to at least one of the 34 types based on the sequence ontology. Variants with high or moderate functional impact, including missense variants, transcript ablation, splice-acceptor variants, splice-donor variants, stop-gained variants, frameshift variants, stop-lost variants, start-lost variants, transcript amplification, in-frame insertion and in-frame deletion, are assorted to the biological effect of CPS. We further scanned the remaining variants for those with biological effects of RGE, consisting of the eQTLs provided by the GTEx database (http://www.gtexportal.org/home/) [77]. We searched for eQTLs in a total number of 202,789 eQTLs obtained from 44 tissues in the GTEx. For each tissue, if a gene contains eQTLs, we selected the eQTL(s) showing the most significant *p* value(s) as a representative. For SNPs belonging to neither CPS nor RGE, if it shows high conservation score, e.g. GERP > 2 (http://cadd.gs.washington.edu) [20] or CADD > 15 (http://mendel.stanford.edu/SidowLab/downloads/gerp/) [19], it is assorted to the biological effect of UCE. SNPs were mapped to genes according to the Ensembl database version 90 (GRCh37, https://asia.ensembl.org/index.html) [78].

### Collection of hypoxia-related pathways (genes) and functional-enrichment analysis

We focused on the adaptive patterns of some genes of particular interest, including genes involved in hypoxia-related pathways defined by PathCards (https://pathcards.genecards.org/); genes reported to be related to hypoxia in previous experimental studies; and genes identified previously as local adaptation in highlanders. A full list of priori candidate genes can be found in Supplementary Table 7. We integrated these genes into a map of hypoxia-induced pathways (Fig. 1D) and then reviewed the pathways and related genes. We calculated the odds ratio to evaluate the enrichment of the candidate adaptive genes in each pathway. Each pathway was tested independently as follows:

$$\begin{equation*}
\mathrm{Odds}\ \mathrm{Ratio}=\frac{A_1}{A_2}/\frac{N_1}{N_2}
\end{equation*}$$

where *A*_1_ denotes the number of candidate adaptive genes involved in the hypoxia-related pathway; *A*_2_ denotes the number of candidate adaptive genes not involved in the hypoxia-related pathway; *N*_1_ denotes the number of non-candidate adaptive genes involved in the hypoxia-related pathway; and *N*_2_ denotes the number of non-candidate adaptive genes not involved in the hypoxia-related pathway. The sum of *A*_1_, *A*_2_, *N*_1_ and *N*_2_ is the total number of genes across the genome. An odds ratio significantly above 1 (*p* < 0.05, the Fisher’s exact test) indicated that the candidate adaptive genes are enriched in the hypoxia-related pathway (Fig. 1C and Supplementary Table 7).

### Detection of natural selection and identification of candidate AGVs

We detected signatures of natural selection primarily based on four population genetic statistics for analysing genetic variation within and between populations: *F*_ST_, calculated for each SNP following Weir and Cockerham [79] using an in-house computer script; the difference in the allele frequency (ΔAF) between TIB and HAN; the integrated haplotype score (iHS) [80] in TIB, estimated with *Selscan* version 1.2.0 (https://github.com/szpiech/selscan) [81]; and the cross-population extended haplotype homozygosity (XP-EHH) [82], also estimated in *Selscan* [81] using HAN as the reference population for TIB. We further conducted CMS analysis [83] using estimates of the above four statistics as inputs. These analyses were restricted to the 4.63 million SNPs with minor allele frequencies >0.05 and known ancestral alleles. The CMS score for each selected SNP was calculated as follows:

$$\begin{equation*}
\mathrm{CMS}=-\log \prod_{{i}}{{p}}_{{i}}
\end{equation*}$$

where *p_i_* is the empirical *p* value of the *i*^th^ test. We divided the whole genome into overlapping regions, each spanning 30 kb, with a step of 15 kb. We considered one region as an adaptive candidate if more than 30% of the variants encompassed had significant CMS scores (in the top 1% across the whole genome). Using this approach, 374 candidate regions were identified in total (Supplementary Table 3).

Candidate AGVs were further selected from the 374 candidate regions, using three criteria. First, selected SNPs had significant CMS scores (in the top 1% across the whole genome). Second, selected SNPs were likely to be highly differentiated between TIB and HAN (>5 × average *F*_ST_ across the genome). To avoid signals attributed to HAN, we ensured that the allele frequency (AF) in TIB was different from that of HAN, and different from the average AF in East Asian populations annotated by VEP. Third, we required the selected SNPs to have possible biological effects, e.g. CPS, RGE and UCE. For each candidate AGV, the allele with a higher frequency in TIB over HAN was regarded as the adaptive allele.

### Calculating the FIS for each gene

Based on the conservation score (*CS*) provided by the various annotation methods (i.e. CADD [19], GERP [20], SIFT [84] and PolyPhen [85]), we measured the accumulated effects of each candidate AGV in the TIB population relative to the HAN population as

\begin{equation*} {\it FIS}_{i}={\it CS}_{i} \times | \Delta {\it AF}| \end{equation*}

where *FIS_i_* is the functional importance score calculated by the *i^th^* method; and ΔAF is the difference in AF between TIB and HAN. We then defined an integrated score for the *j^th^* candidate AGV based on the rank of *FIS* in each of the *n* methods as:

\begin{equation*} {\mathrm{Rank}}_{{j}}=\frac{\sum_{{i}=1}^{{n}}{\mathrm{Rank}}_{\it{i}{j}}}{{n}} \end{equation*}

For each gene carrying at least one candidate AGV, we selected the candidate AGV showing the highest *Rank* (which indicated the greatest degree of functional importance) as the key candidate AGV for that gene. The *Rank* of the key candidate AGV represents the functional importance of the gene.

In this analysis, we used the conservation scores provided by CADD and GERP. Negative GERP scores, indicating evolutionary neutrality, were converted to 0. We weighted the conservation score according to the biological effects of the candidate AGVs, giving candidate AGVs with CPS or RGE effects the maximum *CS*. Gene rankings are shown in Supplementary Table 6.

### Estimating the associations of candidate AGVs with phenotypes in Tibetans

We conducted the association analysis to detect phenotype-associated candidate AGVs in the 2,849 Tibetan subjects inhabiting the Tibetan plateau in Sichuan, China [18]. Two approaches were applied, based on a linear model and a mixed linear model, respectively. We first applied a principal component analysis to the 2,849 Tibetan samples, using 26,520 independent SNPs that were over 100 kb distant from each other. Then, we performed the linear-regression analysis under the additive model implemented in PLINK version 1.07 (http://zzz.bwh.harvard.edu/plink/) [86] and, alternatively, MLMA-LOCO analysis implemented in GCTA version 1.26.0 [87], taking sex and the first five principal components (PCs) as covariates. Of the 1,877 candidate AGVs, 1,865 were included in the association tests, as the genotypes for the other 12 loci were not successfully imputed. Each of the 62 phenotypes (Supplementary Table 8) was tested independently. To control the genome-wide type I error rate, we used Benjamini-Hochberg (BH) FDR correction, which is implemented in *R* version 3.2.1 [88], to account for multiple testing. We strictly tested 115,630 (= 1,865 × 62) independent hypotheses and used *p* < 0.05 as a significant level.

Two approaches were applied to test whether *EPAS1* and *TMEM247* have independent effects on the adaptive phenotypes of Tibetans. For each trait (e.g. RBC, HGB and HCT), we tested three possible linear-regression models on any pair of variants (denoted as 1 and 2):

\begin{equation*} Y=\alpha +{\beta}_i{X}_i+\varepsilon\ \left(i=1\ or\ 2\right)\ \left(\mathrm{Model}\ 1\right) \end{equation*}

\begin{equation*} Y=\alpha +{\beta}_1{X}_1+{\beta}_2{X}_2+\varepsilon\ \left(\mathrm{Model}\ 2\right) \end{equation*}

\begin{equation*} Y=\alpha {+}{\beta}_1{X}_1+{\beta}_2{X}_2{+}{\beta}_3{X}_1{X}_2+\varepsilon \left(\mathrm{Model}\ 3\right) \end{equation*}

where *Y* is the phenotype vector, *X* is the genotype vector for each variant, *α* is the baseline phenotype level, *β*_1_ and *β*_2_ are effect sizes for two respective variants, *β*_3_ is the joint effect size of the two variants and *ɛ* represents stochastic uncertainties. Model 1 tests for the independent effect of each variant in either *EPAS1* or *TMEM247* on the phenotypes; Model 2 estimates the influence of two variants—one in *EPAS1* and the other in *TMEM247*—on the phenotypes; Model 3 includes an additional interaction variant of the two variants based on Model 2. Next, we applied analysis of variance (ANOVA) to compare the fits of two models (e.g. Model 1 vs. Model 2 and Model 2 vs. Model 3) to evaluate the necessity of each variant to the phenotypic variation and to examine possible interactions between variants. We analysed pairwise combinations of variants—one in *EPAS1* and the other in *TMEM247*—that are associated with RBC, HGB or HCT, respectively. Several *EPAS1* variants reported to be adaptive candidates, e.g. rs4953354, rs372272284 and rs149594770, are also included in our analysis [2,17,59]. Sex and the first five PCs were used as covariates. The alternative approach is the cross-conditional association analysis, in which each phenotype-associated locus in *EPAS1* and *TMEM247* (Supplementary Table 9) were tested as a covariate for the other loci. We performed the linear-regression analysis for each variant–phenotype pair independently, under the additive model implemented in PLINK version 1.07 (http://zzz.bwh.harvard.edu/plink/) [86]. Again, sex and the first five PCs were used as additional covariates.

### Detecting the expression quantitative trait loci (eQTLs)

Using the RNA-Seq data from 57 Tibetan placenta tissue samples [17], we explored the impact of the candidate AGVs on gene expression. First, we mapped the preprocessed reads to the reference genome using STAR (http://code.google.com/p/rna-star/) [89] and the resulting bam files were used as inputs to the RSEM program (http://github.com/deweylab/RSEM) to estimate the gene-expression levels [7]. Linear regressions between gene-expression levels and the imputed allele dosage of 592 coding candidate AGVs were performed using ‘MatrixEQTL’ in *R* package. Batch was included in the model as a covariate. Using *p* < 0.05 (BH-FDR correction for 183,520 (= 592 × 310) tests) as a cutoff, we considered a candidate AGV as an eQTL variant if it was associated with the expression level of a gene no more than 100 kb distant from the candidate AGV. Such genes, in this case, were determined to be *cis*-associated with the candidate AGV (Supplementary Table 11).

### Colocalization test for the eQTLs and the phenotype-associated loci

We scanned each 100 kb window across the genome for the colocalization of eQTLs and phenotype-associated loci in Tibetans and found three genomic regions encompassing both signals. The zoom-in plots of the three regions are shown in Fig. 2B. Each region was tested independently using the imputed full genotype data of 57 Tibetans and those of 2,849 Tibetans. For each gene–phenotype pair, we selected a set of four SNPs (two eQTLs and two phenotype-associated loci) and went through all the combinations. For the region on chromosome 2, we treated *EPAS1* and *TMEM247* separately considering that they are in different LD blocks (Supplementary Fig. 6A). The *EPAS1* analysis was restricted to the signals located in *EPAS1* (the intergenic variant rs1900592 was also included), while the *TMEM247* analysis included all the signals in *TMEM247* and five other downstream genes in the same LD block. We performed the statistical test using an R package *coloc* version 3.1 [47] and conducted BH-FDR correction for multiple tests (1,000 tests (= 360 SNP sets for UA + 280 SNP sets for RBC + 150 SNP sets for RGB + 210 SNP sets for HCT) for *EPAS1*; 30,840 tests (= 780 SNP sets for UA + 12,750 SNP sets for RBC + 7,410 SNP sets for RGB + 9,900 SNP sets for HCT) for *TMEM247*; 15 tests for *DUS3L*). We did not test the colocalization of signals in the *PGAP3* region on chromosome 17, as only one phenotype-associated locus was identified in this region but *coloc* analyses consider two loci for each trait. Adjusted *p* < 0.05 was used to reject the null hypothesis of a shared causal variant for the gene expression and phenotype variation.

### Selecting for HAA-related candidate genes

HAA-related candidate genes were further selected from the 521 candidate adaptive genes using these criteria: (i) selected genes should be associated with HAA-related traits (70 in total, listed in Supplementary Table 17) or involved in hypoxia-related pathways in previous studies or in public databases (Supplementary Table 7) or (ii) selected genes should be significantly associated with any of the 62 quantitative traits measured in the 2,849 Tibetan samples (listed in Supplementary Table 8). The 157 candidate HAA-related genes are highlighted in Supplementary Table 6.

### Estimating the correlation between adaptive AF of the candidate AGVs and altitude

To investigate possible relationship between the candidate AGVs and the altitude, we grouped the 33 sequenced Tibetan individuals according to the geographical regions and calculated the correlation between the frequency of the adaptive allele and altitude. The Tibetan samples were grouped into seven regional populations based on altitude: Lhasa (*n* = 3, at 3,650 m), Nyingchi (*n* = 2, at 3,000 m), Chamdo (*n* = 6, at 3,240 m), Shannan (*n* = 7, at 3,573 m), Shigatse (*n* = 8, at 3,853 m), Nagqu (*n* = 3, at 4,522 m) and Dingri (*n* = 4, at 4,300 m). The results are shown in Supplementary Table 4. When assessing the altitudinal correlation of the key locus in *TMEM247* (rs116983452-T), we additionally included 5 Sherpa samples, 39 HAN samples and other Asian samples covering a wider geographical area (unpublished data). The altitude was determined by where the recruited sample currently resided. The correlation coefficient (*r*) was estimated using *R* version 3.2.1 [88].

### Identification of archaic sequences in modern human genomes and local ancestry inference for *TMEM247* and *EGLN1*

We identified genomic segments of non-modern-human origin using *ArchaicSeeker* version 2.1, an improved version of *ArchaicSeeker* [16], in this study. Compared with the old version, *ArchaicSeeker* version 2.1 adopted a hidden Markov model to determine the precise boundaries of the introgressed segments and used a likelihood-based segmental matching algorithm to assign the accurate ancestry to each segment. Using this method, we detected archaic segments in the TIB genomes, especially in *TMEM247*.

The archaic ancestry in *TMEM247* was further confirmed by the S*-statistic analysis [90], which helps to identify genomic segments that could not have been derived from modern human genomes [90,91]. We calculated S* for each 50-kb region of the genome, stepping by 20 kb. The significance of S* for each segment can be calculated by simulating a null distribution of S* in the case of no archaic human introgression. This simulation was performed with *ms* (http://home.uchicago.edu/~rhudson1/source/mksamples.html) [92], using the demographic parameters well established in previous publications (Supplementary Fig. 8 and Supplementary Table 18) [93,94]. The full sequence data of Africans (YRI), Europeans (CEU) and East Asians (CHB) from the 1000 Genomes Project Phase III panel (http://www.1000genomes.org/data) were used in simulation. The recent explosions of the three continental populations were also taken into consideration following Vernot *et al.* [90].

To investigate the fine-scale genetic make-up of some interesting regions, specifically *TMEM247* and *EGLN1*, we developed a method based on the results of ChromoPainter (https://people.maths.bris.ac.uk/∼madjl/finestructure-old/chromopainter_info.html) [95] to obtain the ancestry make-up of a particular genomic region in TIB. The major advantage of this method is that it does not require more than one individual in each of the reference panels, which is different from most existing methods for local ancestral inference [95–98]. We first applied ChromoPainter, using Han Chinese genomes and available archaic genomes, including an Altai Neanderthal genome (https://www.ebi.ac.uk/ena/data/view/PRJEB1265) [99], a Denisovan genome (https://www.ebi.ac.uk/ena/data/view/PRJEB2263) [100] and a 45,000-year-old Siberian genome (Ust’-Ishim, https://www.ebi.ac.uk/ena/data/view/PRJEB6622) [101] as reference data. The Ust’-Ishim genome was included based on the observation that Tibetans share a considerable proportion of their ancestry with Siberian populations. The African genomes (YRI) were also integrated into our reference population panel to avoid the false-positive inference of archaic ancestral segments.

SNPs with more than 50% missing genotypes and their 100-bp flanking regions (both upstream and downstream) were filtered out prior to the analysis. A recombination map with a mean recombination rate of 1 cM per Mbp was used to avoid any bias introduced by a prior recombination map based on some particular populations. For each run of the analysis, *−ip* and *-b* commanders were used to maximize over-copying proportions using an E-M algorithm and obtain the matrix of probability of each recipient copy of each donor at every site. To provide a comparable sample size for the five reference populations, we selected one individual from each of them and ran 4212 replications of ChromoPainter to make full use of the reference samples (1 Denisovan × 1 Neanderthal × 1 Ust’-Ishim × 39 Han × 108 YRI = 4,212). Then, we obtained 4,212 matrices of the copy probability of each haplotype of TIB individuals at each site and used them to determine the ancestry of each allele. We denoted the copy probability as *P_ijkl_*, where *i* is the number of ancestry combinations (*i* = 1, 2, …, 4,212), *j* is the inferred ancestry (*j* = *j_Mod_* for modern human ancestry; *j* = *j_Arch_* for archaic hominin ancestry), *k* denotes the haplotypes of the admixed populations and *l* denotes the physical position.


\begin{eqnarray*} {\mathrm{Anc}_{kl}= \Bigg\lbrace\begin{array}{cc} {j}^{\prime }&\quad \mathrm{Case}\ \mathrm{I}\sim \mathrm{III}\\[4pt] \mathrm{Uncertain}&\quad \mathrm{Case}\ \mathrm{IV}\sim \mathrm{VI}\end{array}}\end{eqnarray*}

where Case I: $\mathrm{count}({P}_{i{j}_{YRI} kl}>0.8)\le 42,{j}^{\prime }={j}_{Arch}$

Case II: $\mathrm{count}({P}_{i{j}_{Mod} kl}>0.8)\le 421,{j}^{\prime }={j}_{Arch}$

Case III: ${j}^{\prime }={j}_{Mod}$

Case IV: $\mathrm{count}({P}_{i{j}_{YRI} kl}>0.8)>42$

Case V: $\mathrm{count}({P}_{i{j}_{Mod} kl}>0.8)>421$

Case VI: None of Case I ∼ V

At a given site, we counted the runs of *P_ijkl_* larger than 0.8 for each reference population. For the sites with possible modern human ancestry, if the maximum count of runs was larger than 421 (10% of total runs) for a particular reference population, then the ancestry of the allele at that site was inferred as the reference population, while, for a potential archaic site, we required the counts of runs of ${P}_{i{j}_{YRI} kl}$ to be smaller than 42 (1% of total runs) and that of ${P}_{i{j}_{Mod} kl}$ to be smaller than 421. In other cases, the site was treated as an uncertain ancestry.

Based on inferred local ancestry, we classified the genomic segments of TIB into six categories: Denisovian-like sequences, Neanderthal-like sequences, Ust’-Ishim-like sequences, Han-Chinese-like sequences, African-like sequences and sequences with uncertain ancestry (the ancestry could not be determined based on the reference sequence possibly due to an unknown archaic origin or the high similarity among different reference sequences).

### Validation studies of rs116983452

To further validate rs116983452 in larger samples, we collected samples from 1,160 native Tibetans living at Lhasa (*n* = 285, at 3,680 m), Kangma (*n* = 148, at 4,700 m), Bange (*n* = 478, at 4,801 m) and Langkazi (*n* = 249, at 5,018 m). Molecular inversion probes [102] were used to genotype rs116983452 in the 1,160 Tibetans. We measured 24 physiological traits for these individuals: serum NO level, systolic pulmonary arterial pressure, degree of blood oxygen saturation, HGB, RBC, HCT, mean red cell volume, red cell distribution width, platelets, lymphocyte count, systolic pressure, diastolic blood pressure, heart rate, peak expiratory flow rate (PEF), maximum ventilatory volume (MVV), forced expiratory flow (FEF), forced expiratory volume in 1 second (FEV1), forced vital capacity (FVC), FEV1/FVC (FFR), height, weight, body mass index (BMI), chest circumference and hip circumference.

We evaluated the genetic association between rs116983452 and 24 physiological traits using PLINK 1.07 [86], under the additive model. We split four populations with the association analysis and then performed a meta-analysis by testing the homogeneity of different population datasets. Sex, age and altitude were treated as covariates where applicable. Especially, BMI was added into the list of covariates when analysing the lung functions, including PEF, MVV, FEF, FEV1, FVC and FFR. For multiple test correction, we used BH-FDR control to adjust the *p* value across the 24 traits.

### Estimating the TMRCA

The TMRCAs of haplotypes carrying the adaptive allele at rs116983452 in *TMEM247* and at rs186996510 in *EGLN1* were independently calculated in the 38 Tibetan samples, based on the average pairwise nucleotide differences of the haplotypes ($\overline{\pi}$) as follows:

\begin{equation*} \overline{\pi}=\frac{2\times \sum_{i=1}^{n-1}\sum_{j=i+1}^n{\pi}_{ij}}{\left(n-1\right)\times n} \end{equation*}

where *n* is the number of sequences in a given region and *π_ij_* is the nucleotide difference between the two sequences *i* and *j* (*i* ≠ *j*).

The TMRCA was then estimated as

$$\begin{equation*}
{\it TMRCA} = \frac{\overline{\pi}}{2\times {\mu}_{ab}\times {l}_{ab}}
\end{equation*}$$

where *μ_ab_* is the local mutation rate of a genomic region with length *l*_ab_ started from position *a* to position *b*. The value of *μ_ab_* was estimated as

\begin{equation*} {\mu}_{{ab}}=\frac{{{d}}_{\mathrm{Hum}-\mathrm{AncHumChimp}}}{{{l}}_{{ab}}\times {{T}}_{\mathrm{Hum}-\mathrm{HumChimp}}} \end{equation*}

where *d*_Hum–AncHumChimp_ denotes the nucleotide difference between human reference genome and the Human-Chimp-Ancestor of region *ab*. *T_Hum–HumChimp_* is the divergence time between humans and chimps and was here set to 13 million years.

*TMEM247* and *ATP6V1E2* were in an LD block (Supplementary Fig. 6). Therefore, we considered the entire block in this calculation, the boundary of which (Chr2:46657114–46772997) was identical to that of the TIB-specific haplotype reported in Lu *et al.* [16]. To eliminate the inter-ancestral recombination, we used 26 TIB-specific markers (Supplementary Table 19) as integral TIB-specific haplotypes. Interestingly, most of the haplotypes carrying rs116983452-T, 53 in total, also carry the derived alleles at these 26 loci. The TMRCA of these 53 haplotypes was estimated to be 56,200 ± 24,800 years. In *EGLN1*, there were 40 haplotypes carrying rs186996510-G and the TMRCA was estimated to be 29,800 ± 24,200 years.

We used *startmrca* [103] in *R* package to validate the TMRCAs of haplotypes carrying rs116983452-T. *startmrca* uses a hidden Markov model taking into account the length distribution of the shared ancestral haplotype, the accumulation of derived mutations and the surrounding background haplotype diversity. We ran this analysis five times, each including 15,000 iterations. We took the acceptable TMRCA estimations in the last 6,000 iterations of each run as the final results. A mutation rate of 1.25 × 10^−8^ per site per generation was used for this estimation. Consistently with our previous TMRCA analysis, *startmrca* estimated a TMRCA of 58,100 ± 2,800 years.

### Estimation of selection coefficient

Here, we applied a simple deterministic model of selective sweep with additive genetic effects, using the following formula, which is the same as that used in a previous study [60]:

\begin{equation*} s=\frac{1}{t}\log \frac{p_t\left(1-{p}_0\right)}{p_0\left(1-{p}_t\right)} \end{equation*}

We assumed that selection began right after the split of the Tibetan and the Han Chinese. We therefore used an estimated divergence time of 9,000–15,000 years (360–600 generations, assuming a generation time of 25 years) between TIB and HAN [16] as an approximation of the onset of selection (*t*) in TIB. We took the AF in HAN and TIB as approximates of the initial AF (*p*_0_) and the current AF (*p_t_*), respectively. For rs116983452, we also applied an alternative hypothesis, which assumed that selection occurred right after the introgression of the beneficial allele in TIB from the Denisovan. For this analysis, we used the TMRCA of haplotypes carrying the derived allele at rs116983452, which was estimated to be around 60,000 YBP (2,400 generations), as an approximate of *t* and estimated *p*_0_ to be 1/2*N*_e_ (*N*_e_ = ∼ 1,000–3,000 around 60,000 years ago; see Supplementary Table 14 for more details).

## Supplementary Material

nwz108_Supplemental_File

Table_S3_nwz108

Table_S4_nwz108

Table_S6_nwz108

Table_S7_nwz108

Table_S12_nwz108

Table_S13_nwz108
