# Supplementary material for: Prioritizing natural-selection signals from the deep-sequencing genomic data suggests multi-variant adaptation in Tibetan highlanders
Source: Natl Sci Rev. 2019 Aug 7;6(6):1201–22. doi: 10.1093/nsr/nwz108 (PMC8291452; doi:10.1093/nsr/nwz108)
Supplement: nwz108_Supplemental_File [file nwz108_supplemental_file.docx]

**Supplementary Data for**

**Prioritizing natural selection signals from the deep-sequencing genomic data suggests multi-variant adaptation in Tibetan**

*Running title:* Prioritizing adaptive variants in Tibetans

Lian Deng, Chao Zhang, Kai Yuan, Yang Gao, Yuwen Pan, Xueling Ge, Yaoxi He, Yuan Yuan, Yan Lu, Xiaoxi Zhang, Hao Chen, Haiyi Lou, Xiaoji Wang, Dongsheng Lu, Jiaojiao Liu, Lei Tian, Qidi Feng, Asifullah Khan, Yajun Yang, Zi-Bing Jin, Jian Yang, Fan Lu, Jia Qu, Longli Kang, Bing Su, Shuhua Xu

Supplementary Data include 8 figures and 19 tables:

**Fig. S1** A protein-protein interaction (PPI) network for the candidate adaptive genes that may play roles in the HIF-related pathway.

**Fig. S2** Significant correlations between the candidate AGVs and gene expression levels in term placenta.

**Fig. S3** Linkage disequilibrium (LD) between eQTLs in Tibetans.

**Fig. S4** Two genomic regions with great genetic differentiation between TIB and HAN.

**Fig. S5** Correlation between the frequency of rs116983452-T and the altitude in Tibetan samples.

**Fig. S6** Linkage disequilibrium (LD) pattern of *TMEM247* and the surrounding genes.

**Fig. S7** The inferred local ancestry in *EGLN1* and *TMEM247*.

**Fig. S8** Demographic model used in simulation.

**Table S1.** Summary of SNVs discovered from deep whole-genome sequencing.

**Table S2.** Number of SNVs in each impact group.

**Table S3.** Top 1% genomic regions with significant CMS score. (see Table_S3.xlsx)

**Table S4.** A list of candidate AGVs. (see Table_S4.xlsx)

**Table S5.** Full list of missense and loss-of-function candidate AGVs.

**Table S6.** A list of candidate adaptive genes. (see Table_S6.xlsx)

**Table S7.** Priori functional candidate genes. (see Table_S7.xlsx)

**Table S8.** Summary of the 62 quantitative traits in Tibetans.

**Table S9.** Significant associations between the candidate AGVs and traits identified by the linear model.

**Table S10.** Significant associations between the candidate AGVs and traits identified using MLMA-LOCO.

**Table S11.** A list of eQTLs identified in the candidate AGVs in the Tibetans.

**Table S12.** Colocalization test for eQTLs and phenotype-associated candidate AGVs. (see Table_S12.xlsx)

**Table S13.** Effects of the candidate AGVs in *EPAS1* and those in *TMEM247* on the adaptive traits in Tibetans. (see Table_S13.xlsx)

**Table S14.** Selection coefficient estimated for rs116983452 in TIB.

**Table S15.** Validation of the association between rs116983452-T and hypoxia-related traits.

**Table S16.** Cross-conditional association analysis of *TMEM247* and *EPAS1*.

**Table S17.** HAA-related traits collected from literatures.

**Table S18.** Parameters used in the simulation.

**Table S19.** TIB-specific markers used in the calculation of TMRCA for the haplotypes carrying rs116983452-T.

**
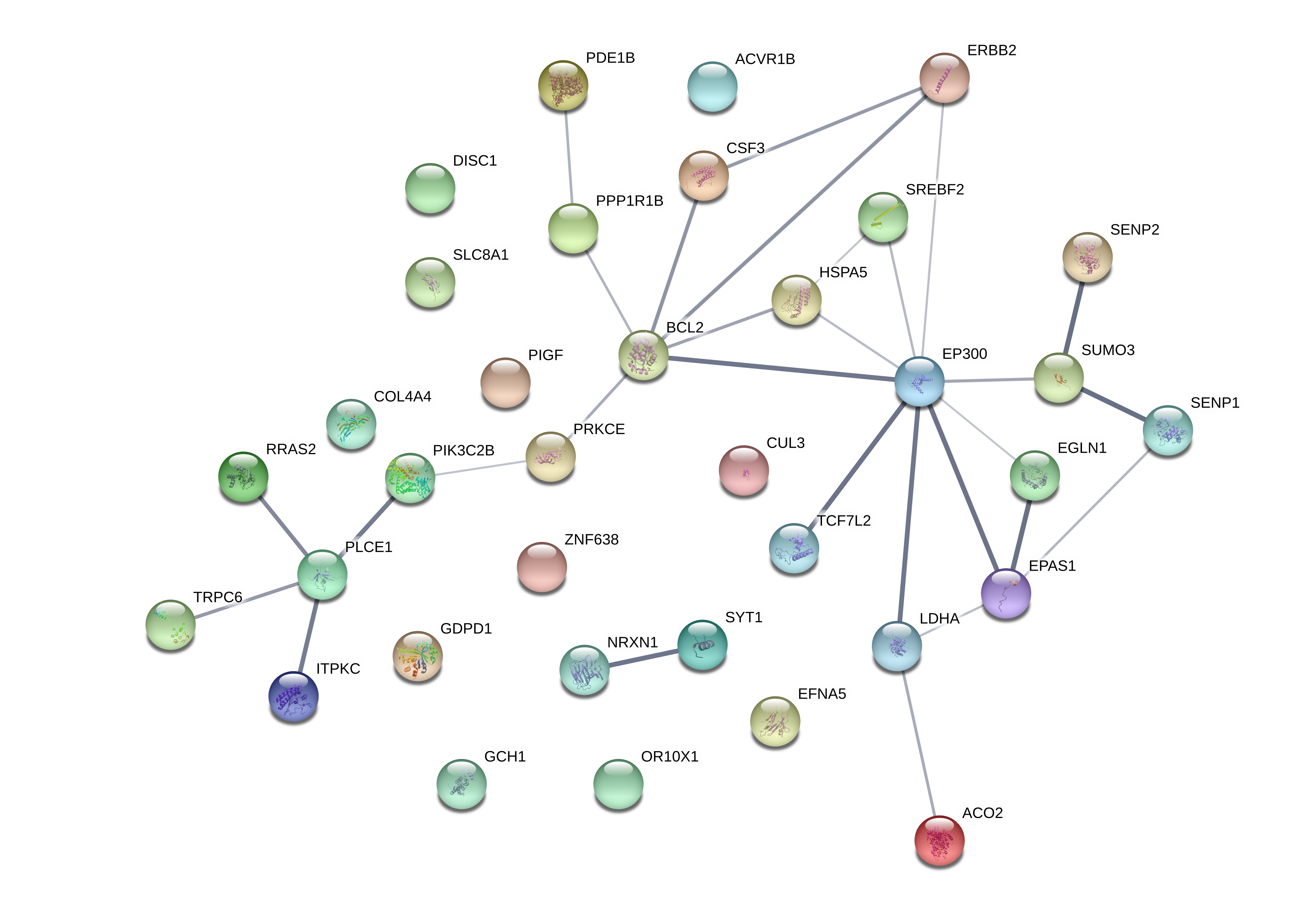
**

**Fig. S1 A protein-protein interaction (PPI) network for the candidate adaptive genes that may play roles in the HIF-related pathway.**

The network was drawn using String (<http://string-db.org/)>, which shows significant interactions among the candidate genes presented in Fig. 1D against a random set of proteins of similar size (PPI enrichment *p* = 2.65 × 10^-4^). The thickness of the edges indicates the confidence of the interactions.

**Fig. S2 Significant correlations between the candidate AGVs and gene expression levels in term placenta.**

Each plot shows the expression of a gene in three genotypes of an eQTL, with the homozygotes of adaptive alleles on the right.

**Fig. S3 Linkage disequilibrium (LD) between eQTLs in Tibetans.**

LD was measured by *r^2^* using Haploview version 4.2 (A) in the 57 Tibetans samples used in the eQTL analysis, (B) in the 2,849 Tibetan samples used in the phenotype association study, and (C) in the 38 deep-sequenced Tibetan samples. In each plot, the physical positions of genes are indicated above the chromosome, with cis-regulated genes noted by colored bars and with bold fonts. For genomic regions encompassing multiple cis-regulated genes, the eQTLs are labeled with dots in consistent colors with the associated genes. The eQTLs with missense variants are highlighted in red.

** Fig. S4 Two genomic regions with great genetic differentiation between TIB and HAN.**

(A) Genome-wide distribution of *F*_ST_. The vertical line in red indicates the top 0.1% threshold of *F*_ST_ (*F*_ST_ = 0.155). (B) Zoomed-in plots for the two outstanding signal genomic regions. Genes are displayed on the top of each plot. The missense candidate AGVs are presented by dots in red, including rs186996510 in *EGLN1* and rs12612916, rs116983452 and rs196290066 in *TMEM247*. In both plots, the candidate AGVs in the two highly differentiated regions between TIB and HAN are indicated by blue dots.

**
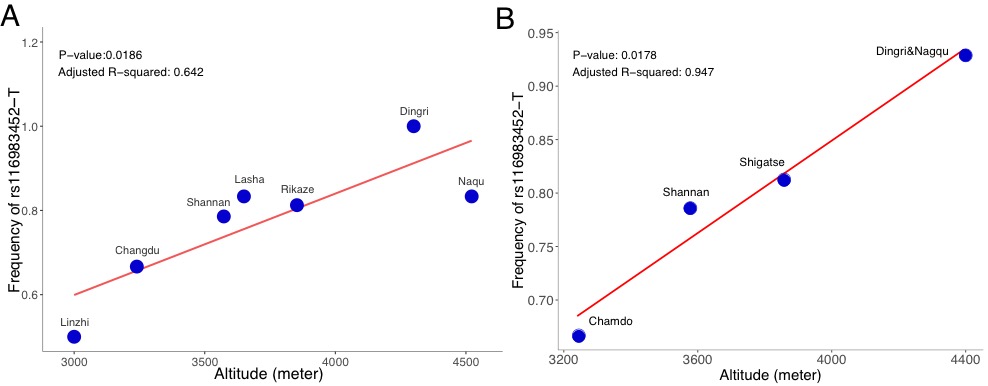
**

**Fig. S5 Correlation between the frequency of rs116983452-T and the altitude in Tibetan samples.**

(A) Correlation calculated for 7 Tibetan subpopulations according to the altitudinal information (see Methods). (B) Correlation calculated for 4 selected Tibetan subpopulations. As the sample size of each subpopulation is rather small in (A), we selected four of the groups with larger sample size, including Chamdo (6 samples), Shannan (7 samples), Shigatse (9 samples) and Dingri&Nagqu (6 samples), to calculate the correlation. Dingri and Nagqu samples were combined to enlarge the group as they are located at higher altitude than all the other samples.

**Fig. S6 Linkage disequilibrium (LD) pattern of *TMEM247* and the surrounding genes.**

(A) Recombination map and LD pattern of *TMEM247* and its surrounding genes. The upper panel shows the recombination map obtained from the HapMap Project. The red dash line indicates the position of the recombination hotspot as shown in the above map. The lower panel shows that *TMEM247* is in a large LD block with the 4 downstream genes, but independent from *EPAS1*. The LD was measured by the 4 gamete frequencies using Haploview version 4.2, from which the D’, LOD and r^2^ calculations are derived. The white unit indicates 4 distinct 2-marker haplotypes, suggesting possible recombination events between pairs of sites; the black unit indicates < 4 distinct 2-marker haplotypes. (B) LD pattern of the important AGVs in *TMEM247* and *EPAS1*, with several reported candidate adaptive variants. The variants are labeled as: ^1^, the missense variants in *TMEM247*; ^2^, the key candidate AGV (an eQTL) in *EPAS1*; ^3^, SNPs in the same frequency block with TED (1); ^4^, the 5-SNP-motif with Denisovan origin (2); ^5^, a regulatory variant and its putative interacted variant in *EPAS1* (3); ^6^, two intronic variant in *EPAS1* and a variant downstream to *TMEM247* (4). These variants lie in two LD-blocks. The LD was presented using the standard color scheme based on D’ and LOD (white: D’ < 1 and LOD <2; blue: D’ = 1 and LOD < 2; shades of pink/red: D’ < 1 and LOD ≥ 2; bright red: D’ = 1 and LOD ≥ 2).

**
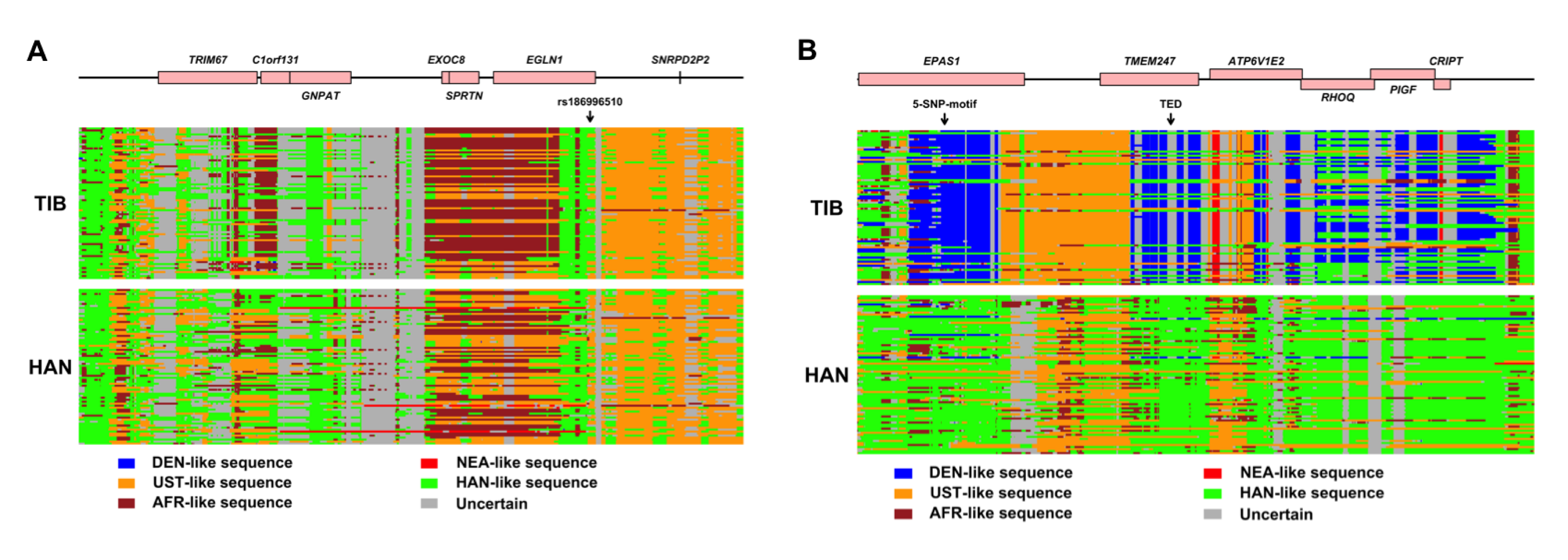
Fig. S7 The inferred local ancestry in *EGLN1* and *TMEM247*.**

The detailed local ancestry of two regions (Chr1:231250000-231650000 and Chr2:46520000-46900000), covering *EGLN1* and *TMEM247*, are shown in (A) and (B), respectively. From the above to the bottom, it includes (1) genes located in each (the pink box); (2) several important variants reported in previous studies (the vertical arrows), e.g., two Tibetan-specific variant tags 5-SNP-motif (2) and TED (1) (in plot A), and a high-frequency missense mutation in *EGLN1* contributing functionally to the Tibetan high-altitude phenotype (5, 6) (in plot B); (3) the inferred ancestry of haplotypes in TIB and HAN, with each row represents a haplotype with ancestry derived from NEA (red), DEN (blue), UST (orange), HAN (green) and AFR (brown), and with uncertain ancestry (grey). The local ancestry was inferred with ChromoPainter (7) (see Methods). DEN, Denisovan; NEA, Neadertal; UST, Ust’-Ishim; AFR, African; HAN, Han Chinese; TIB, Tibetan.

**Fig. S8 Demographic model used in simulation.**

Parameters are listed in Table S17.

**Table S1. Summary of SNVs discovered from deep whole-genome sequencing.**

| Sample | Number of SNVs | |  | Novelty rates of SNVs | | | |  |
| --- | --- | --- | --- | --- | --- | --- | --- | --- |
|  | Population level | Individual level |  | dbSNP 138 | dbSNP 142 | dbSNP147 | dbSNP151 | |
| Total | 11.57 | - |  | 24.8% (2.87) | 19.1% (2.22) | 18.1% (2.09) | 15.1%(1.75) | |
| TIB | 9.18 | 3.56 |  | 17.3% (1.59) | 13.5% (1.24) | 12.7% (1.16) | 11.1%(1.02) | |
| HAN | 9.52 | 4.29 |  | 17.6% (1.68) | 13.6% (1.30) | 12.8% (1.22) | 11.3%(1.08) | |

The novelty rates of SNV calls were calculated based on the dbSNP build 138, 142, 147 and 151. The numbers in the brackets are the absolute number of SNVs in million.

**Table S2. Number of SNVs in each impact group.**

| Variants types | All samples | TIB | HAN |
| --- | --- | --- | --- |
| High impact (Loss of function) | | | |
| splice_donor_variant | 994 | 740 | 787 |
| splice_acceptor_variant | 716 | 520 | 555 |
| stop_gained | 926 | 582 | 639 |
| stop_lost | 154 | 124 | 122 |
| initiator_codon_variant | 210 | 154 | 162 |
| Moderate impact |  |  |  |
| missense_variant | 53473 | 37153 | 40249 |
| Low impact |  |  |  |
| splice_region_variant | 13070 | 10034 | 10465 |
| incomplete_terminal_codon_variant | 5 | 5 | 3 |
| stop_retained_variant | 59 | 46 | 48 |
| synonymous_variant | 41338 | 31485 | 32946 |
| Modifier |  |  |  |
| coding_sequence_variant | 7 | 5 | 4 |
| mature_miRNA_variant | 346 | 267 | 289 |
| 5_prime_UTR_variant | 36842 | 27821 | 29144 |
| 3_prime_UTR_variant | 117204 | 90006 | 94102 |
| non_coding_transcript_exon_variant | 173025 | 135919 | 140954 |
| intron_variant | 5803695 | 4575457 | 4756732 |
| non_coding_transcript_variant | 1 | 1 | 1 |
| upstream_gene_variant | 554422 | 439372 | 459407 |
| downstream_gene_variant | 451479 | 358788 | 373258 |
| TF_binding_site_variant | 2762 | 2100 | 2239 |
| regulatory_region_variant | 280701 | 223518 | 231153 |
| intergenic_variant | 4042536 | 3243163 | 3350673 |
| All | 11573965 | 9177282 | 9523954 |

The variant type of each SNV was predicted by variant effect predictor (VEP). As different types of variants have diverse functional impacts on the genome, the VEP estimated the severity of each variant effect of the variants and categorized them into 4 degrees, i.e., high impact, moderate impact, low impact and modifier. We defined the variants with high impact as Loss-of-Function (LoF) variants and those belong to the low impact and modifier groups as natural ones.

**Table S3. Top 1% genomic regions with significant CMS score.**

The whole genome was divided into regions, each spanning 30kb with 15kb as slide steps. We considered one region as adaptive candidate if there are more than 30% of the variants with significant CMS scores in it. The adjacent regions were combined. The column of “Putative AGVs” lists the candidate AGVs with biological effects of changing protein sequencing (CPS), regulating gene expression (RGE) and unknown but conserved in evolution (UCE) in the corresponding region. The short line indicates that there’s no gene reported or no putative AGV identified in that region.

**Table S4. A list of candidate AGVs.**

CPS: changing protein sequence; RGE: regulating gene expression; UCE: unknown but conserved in evolution. For the candidate AGVs in the category of RGE, we also provide the tissues in which the gene expression is regulated by the candidate AGV. Significant correlation between the altitude and the beneficial allele frequency was noted by an asterisk.

**Table S5. Full list of missense and loss-of-function candidate AGVs.**

| **Chr** | **SNP** | **Gene** | **Mutation** | | ***F*_ST_** | **Frequency** | | | |
| --- | --- | --- | --- | --- | --- | --- | --- | --- | --- |
|  |  |  | **Nucleotide** | **Amino acid** |  | **TIB** | **HAN** | **ASN** | **Global** |
| 2 | rs116983452 | *TMEM247* | c.248C>T | p.Ala83Val | 0.72 | 0.76 | 0.03 | 0.02 | 0.006 |
| 2 | rs192690066 | *TMEM247* | c.41C>T | p.Ala14Val | 0.35 | 0.36 | 0.00 | 0.0017 | 0.0005 |
| 2 | rs12612916 | *TMEM247* | c.460G>A | p.Glu154Lys | 0.32 | 0.92 | 0.53 | 0.49 | 0.81 |
| 1 | rs186996510 | *EGLN1* | c.12C>G | p.Asp4Glu | 0.45 | 0.53 | 0.04 | 0.01 | 0.01 |
| 1 | rs6679056 | *OR10R2* | c.647A>G | p.Glu216Gly | 0.24 | 0.84 | 0.49 | 0.61 | 0.55 |
| 1 | rs1418843 | *OR10R2* | c.715C>T | p.Leu239Phe | 0.24 | 0.84 | 0.49 | 0.61 | 0.47 |
| 2 | rs1804020 | *ZNF638* | c.1996G>A | p.Val666Met | 0.23 | 0.87 | 0.53 | 0.66 | 0.35 |
| 21 | rs2838697 | *SUMO3* | c.205G>T | p.Val69Phe | 0.22 | 0.78 | 0.42 | 0.42 | 0.68 |
| 1 | rs863363 | *OR10X1* | c.179T>C | p.Ile60Thr | 0.21 | 0.84 | 0.51 | 0.62 | 0.55 |
| 1 | *rs863362 | *OR10X1* | c.198G>A | p.Trp66Ter | 0.21 | 0.84 | 0.51 | 0.62 | 0.53 |
| 22 | rs2228313 | *SREBF2* | c.2580G>C | p.Arg860Ser | 0.21 | 0.37 | 0.08 | 0.08 | 0.07 |
| 22 | rs2075939 | *NCF4* | c.815T>C | p.Leu272Pro | 0.20 | 0.93 | 0.65 | 0.70 | 0.82 |
| 6 | rs4946188 | *ZUFSP* | c.1135A>G | p.Asn379Asp | 0.20 | 0.55 | 0.22 | 0.30 | 0.37 |
| 2 | rs3827760 | *EDAR* | c.1205T>C | p.Val402Ala | 0.20 | 0.32 | 0.05 | 0.12 | 0.71 |
| 4 | rs17029277 | *RP11-766F14.2* | c.95G>A | p.Arg32Gln | 0.20 | 0.91 | 0.62 | 0.59 | 0.78 |
| 4 | rs4689254 | *ZBTB49* | c.1042G>A | p.Ala348Thr | 0.20 | 0.88 | 0.58 | 0.66 | 0.85 |
| 4 | rs75060923 | *ZBTB49* | c.274G>A | p.Glu92Lys | 0.20 | 0.88 | 0.58 | 0.66 | 0.89 |
| 8 | rs2271111 | *DOCK5* | c.3068A>G | p.Gln1023Arg | 0.19 | 0.88 | 0.59 | 0.67 | 0.71 |
| 11 | *rs7105857 | *COLCA2* | c.-285+2C>T | NA | 0.18 | 0.80 | 0.49 | 0.61 | 0.67 |
| 22 | rs2272843 | *MOV10L1* | c.3536C>A | p.Ala1179Glu | 0.18 | 0.42 | 0.13 | 0.19 | 0.13 |
| 22 | rs12628964 | *MOV10L1* | c.52A>T | p.Thr18Ser | 0.09 | 0.57 | 0.33 | 0.31 | 0.24 |
| 22 | rs9617066 | *MOV10L1* | c.109A>T | p.Met37Leu | 0.09 | 0.57 | 0.33 | 0.31 | 0.24 |
| 2 | rs2272051 | *DUSP11* | c.107A>G | p.Asp36Gly | 0.18 | 0.58 | 0.26 | 0.31 | 0.38 |
| 17 | rs1877031 | *STARD3* | c.350G>A | p.Arg117Gln | 0.18 | 0.68 | 0.36 | 0.48 | 0.49 |
| 1 | rs2295283 | *MIIP* | c.499A>G | p.Lys167Glu | 0.18 | 0.78 | 0.46 | 0.49 | 0.66 |
| 2 | rs2037814 | *ALMS1* | c.2012T>G | p.Val671Gly | 0.18 | 0.51 | 0.21 | 0.23 | 0.14 |
| 2 | rs3820700 | *ALMS1* | c.2555G>A | p.Ser852Asn | 0.18 | 0.51 | 0.21 | 0.23 | 0.14 |
| 2 | rs2017116 | *ALMS1* | c.2848G>C | p.Asp950His | 0.18 | 0.51 | 0.21 | 0.23 | 0.15 |
| 2 | rs1052161 | *ALMS1* | c.11960G>A | p.Arg3987Lys | 0.17 | 0.59 | 0.28 | 0.28 | 0.42 |
| 3 | rs6762208 | *SENP2* | c.872C>A | p.Thr291Lys | 0.18 | 0.51 | 0.21 | 0.30 | 0.36 |
| 15 | rs8182086 | *ZNF592* | c.2777G>A | p.Ser926Asn | 0.18 | 0.76 | 0.45 | 0.55 | 0.66 |
| 1 | rs16853773 | *PIK3C2B* | c.100C>T | p.Arg34Cys | 0.18 | 0.82 | 0.51 | 0.62 | 0.80 |
| 16 | rs3803650 | *SLC7A6OS* | c.134G>A | p.Gly45Asp | 0.17 | 0.53 | 0.22 | 0.20 | 0.30 |
| 4 | rs12648093 | *NUDT6* | c.340T>C | p.Cys114Arg | 0.17 | 0.21 | 0.01 | 0.06 | 0.35 |
| 12 | rs10747561 | *LMBR1L* | c.178G>A | p.Val60Ile | 0.17 | 0.49 | 0.19 | 0.29 | 0.51 |
| 5 | rs7726005 | *MGAT1* | c.668G>A | p.Arg223Gln | 0.16 | 0.93 | 0.69 | 0.81 | 0.84 |
| *(Continued)* | | | | | | | | | |
| 22 | *rs5758511 | *CENPM* | c.7C>T | p.Arg3Ter | 0.16 | 0.76 | 0.46 | 0.49 | 0.73 |
| 2 | rs6740879 | *CCDC138* | c.344G>A | p.Arg115Lys | 0.16 | 0.22 | 0.03 | 0.09 | 0.22 |
| 4 | *rs10008489 | *FRAS1* | c.118T>C | p.Ter40ArgextTer23 | 0.15 | 0.87 | 0.60 | - | - |
| 4 | rs4859905 | *FRAS1* | c.95A>G | p.Asp32Gly | 0.10 | 0.71 | 0.47 | 0.47 | 0.48 |
| 20 | rs6126344 | *SALL4* | c.1520T>G | p.Leu507Arg | 0.15 | 0.62 | 0.32 | 0.43 | 0.65 |
| 2 | rs1800517 | *COL4A4* | c.3011C>T | p.Pro1004Leu | 0.15 | 0.63 | 0.33 | 0.48 | 0.51 |
| 12 | rs11062385 | *KDM5A* | c.2594T>C | p.Met865Thr | 0.15 | 0.82 | 0.54 | 0.51 | 0.35 |
| 3 | rs2289247 | *GNL3* | c.1063G>A | p.Val355Met | 0.15 | 0.72 | 0.44 | 0.51 | 0.51 |
| 3 | rs11177 | *GNL3* | c.80G>A | p.Arg27Gln | 0.13 | 0.72 | 0.45 | 0.52 | 0.61 |
| 3 | rs6617 | *SPCS1* | c.121C>G | p.Pro41Ala | 0.15 | 0.72 | 0.44 | 0.51 | 0.51 |
| 3 | rs1029871 | *NEK4* | c.673C>G | p.Pro225Ala | 0.15 | 0.72 | 0.44 | 0.51 | 0.62 |
| 4 | *rs1459853 | *GUCY1B3* | c.363+1G>A | NA | 0.14 | 0.62 | 0.33 | 0.35 | 0.62 |
| 1 | rs1136410 | *PARP1* | c.2285T>C | p.Val762Ala | 0.14 | 0.82 | 0.55 | 0.54 | 0.76 |
| 17 | rs3744093 | *RNF43* | c.139A>G | p.Ile47Val | 0.14 | 0.76 | 0.49 | 0.52 | 0.39 |
| 17 | rs2257205 | *RNF43* | c.350G>A | p.Arg117His | 0.13 | 0.87 | 0.63 | 0.65 | 0.81 |
| 17 | rs2526374 | *RNF43* | c.1252C>A | p.Leu418Met | 0.11 | 0.72 | 0.47 | 0.45 | 0.40 |
| 7 | rs292592 | *WDR91* | c.770C>T | p.Pro257Leu | 0.13 | 0.74 | 0.46 | 0.59 | 0.66 |
| 19 | rs2305925 | *CATSPERD* | c.2227A>T | p.Thr743Ser | 0.13 | 0.79 | 0.53 | 0.50 | 0.72 |
| 5 | *rs6890099 | *ATOX1* | c.-229+2T>C | NA | 0.13 | 0.63 | 0.36 | 0.29 | 0.23 |
| 20 | rs2296129 | *FAM209B* | c.386A>C | p.Glu129Ala | 0.12 | 0.80 | 0.55 | 0.55 | 0.88 |
| 20 | rs3209183 | *FAM209B* | c.220C>A | p.Gln74Lys | 0.11 | 0.72 | 0.47 | 0.48 | 0.34 |
| 3 | rs4434138 | *STAB1* | c.6844A>G | p.Ile2282Val | 0.12 | 0.72 | 0.46 | 0.50 | 0.58 |
| 3 | rs13303 | *STAB1* | c.7517T>C | p.Met2506Thr | 0.10 | 0.68 | 0.45 | 0.49 | 0.40 |
| 19 | rs3745640 | *PRR22* | c.353C>T | p.Pro118Leu | 0.12 | 0.79 | 0.54 | 0.51 | 0.71 |
| 2 | rs1048013 | *CYP20A1* | c.1060C>T | p.Leu354Phe | 0.12 | 0.87 | 0.64 | 0.72 | 0.62 |
| 17 | rs2302190 | *MTMR4* | c.838A>G | p.Ser280Gly | 0.12 | 0.89 | 0.68 | 0.71 | 0.72 |
| 17 | rs3744108 | *MTMR4* | c.550C>G | p.Leu184Val | 0.11 | 0.74 | 0.49 | 0.52 | 0.40 |
| 17 | rs8073754 | *SEPT4* | c.5G>A | p.Arg2Lys | 0.12 | 0.89 | 0.68 | 0.73 | 0.76 |
| 2 | *rs12623638 | *AC009965.2* | n.136+1G>A | NA | 0.11 | 0.82 | 0.58 | 0.63 | 0.59 |
| 2 | rs4665385 | *AC074091.13* | c.196G>A | p.Gly66Arg | 0.11 | 0.80 | 0.56 | 0.57 | 0.27 |
| 6 | rs1063478 | *HLA-DMA* | c.211G>A | p.Val71Ile | 0.11 | 0.91 | 0.71 | 0.77 | 0.85 |
| 11 | rs3824915 | *ALX4* | c.104G>C | p.Arg35Thr | 0.10 | 0.74 | 0.50 | 0.49 | 0.42 |
| 2 | rs17773492 | *LINC01118* | c.91A>G | p.Asn31Asp | 0.09 | 0.93 | 0.77 | 0.83 | 0.77 |
| 19 | rs3865452 | *ADCK4* | c.280A>G | p.Thr94Ala | 0.08 | 0.68 | 0.46 | 0.47 | 0.46 |

The loss-of-function variants were marked with ‘*’. Variants were annotated based on Ensembl database version 90 using VEP. *F*_ST_ indicates the genetic differentiation between TIB and HAN. Genes coding for non-coding RNAs (e.g. miRNA and LincRNA) are underlined as the missense or LoF effects are hard to confirm. Allele frequencies for Asian (ASN) and global populations (Global) were calculated from the 1000 Genomes database. NA: not applicable.

**Table S6. A list of adaptive candidate genes.**

CPS: changing protein sequence; RGE: regulating gene expression; UCE: unknown but conserved in evolution. Genes were ordered according to the functional importance assessment (see Methods). HAA-related genes are highlighted in red. Genes reported to be HAA-related in Tibetans or show significant association with HAA-related traits in the Tibetans are noted by ‘*’. The information of tissue-specific expression of the HAA-related genes were obtained from GTEx. Reported denotes whether the regions have been reported in previous studies.

|  |
| --- |
| **^References/Databases^** |
| ^(8)^ |
| ^(9-14); PathCards^ |
| ^(15)^ |
| ^-^ |
| ^-^ |
| ^(9-12, 16, 17); PathCards^ |
| ^PathCards^ |
| ^(18)^ |
| ^PathCards^ |
| ^(19)^ |
| ^(15, 20); PathCards^ |
| ^RGD^ |
| ^(21)^ |
| ^-^ |
| ^(16)^ |
| ^PathCards^ |
| ^PathCards^ |
| ^PathCards^ |
| ^PathCards^ |
| ^-^ |
| ^(22)^ |
| ^PathCards^ |
| ^PathCards^ |
| ^(1, 23-25)^ |
| ^(15)^ |
| ^(15); PathCards^ |
| ^-^ |
| ^(18, 21)^ |
| ^(26)^ |
| ^(27).^ |
| ^(19, 21, 28)^ |
| ^(16)^ |
| ^-^ |
| ^(29)^ |
| ^PathCards^ |
| ^(20)^ |
| ^(27)^ |
| ^(1, 24)^ |
| ^(22, 30) ; PathCards;^ |
| ^(18)^ |
| ^(31) ;PathCards^ |
| ^PathCards^ |
| ^(1)^ |
| ^(32)^ |
| ^-^ |
| ^(16)^ |
| ^(1, 33); PathCards^ |
| ^(19)^ |
| ^(19)^ |
| ^PathCards^ |
| ^(19)^ |
| ^(34)^ |
| ^(14, 35)^ |
| ^(16, 36)^ |
| ^(21, 24)^ |
| ^(21, 23)^ |
| ^(16, 20, 22, 37-42) ; PathCards^ |
| ^(19, 28)^ |
| ^(22, 43-45)^ |
| ^(23)^ |
| ^(22, 28)^ |
| ^RGD^ |
| ^(22, 43-46)^ |
| ^PathCards^ |
| ^(19)^ |
| ^(47)^ |
| ^(16, 31)^ |
| ^(13)^ |
| ^(19)^ |
| ^(36, 48-51)^ |
| ^(52)^ |
| ^(53)^ |
| ^(52)^ |
| ^(18, 21, 54)^ |
| ^PathCards^ |
| ^-^ |
| ^-^ |
| ^(21)^ |
| ^PathCards^ |
| ^(16)^ |
| ^(55)^ |
| ^(23, 24, 34, 50, 52)^ |
| ^(56) ;PathCards^ |
| ^PathCards^ |
| ^(19)^ |
| ^(1, 52)^ |
| ^(52, 57, 58)^ |
| ^(19)^ |
| ^(18, 21)^ |
| ^(16, 27) ; PathCards^ |
| ^(13, 52)^ |
| ^-^ |
| ^-^ |
| ^(18)^ |
| ^(31)^ |
| ^(24)^ |
| ^(16, 20, 59, 60)^ |
| ^(23)^ |
| ^(23, 61)^ |
| ^(62)^ |
| ^(19); PathCards^ |
| ^(16); PathCards^ |
| ^(63)^ |
| ^(20)^ |
| ^(19)^ |
| ^(16)^ |
| ^(64)^ |
| ^(22, 65)^ |
| ^(66)^ |
| ^(18) ;PathCards^ |
| ^(52)^ |
| ^(52)^ |
| ^(21, 44, 45)^ |
| ^(52, 67)^ |
| ^(16)^ |
| ^(47, 52, 68)^ |
| ^PathCards^ |
| ^(13, 24)^ |
| ^(20).^ |
| ^(11, 22, 52, 69); PathCards^ |
| ^(18)^ |
| ^(19)^ |
| ^(1, 13, 19, 23, 24, 48, 49, 51, 70-100)^ |
| ^(52)^ |
| ^(52)^ |
| ^(19, 24)^ |
| ^(14, 32, 36, 48, 49, 51, 70, 73, 85)^ |
| ^(85)^ |
| ^(1, 16, 24, 51) RGD; PathCards^ |
| ^(18, 24)^ |
| ^(50)^ |
| ^(20)^ |
| ^(23, 24) (1, 101, 102)^ |
| ^(22)^ |
| ^-^ |
| ^(12, 15)^ |
| ^(100, 103);PathCards^ |
| ^(14, 22)^ |
| ^(104)^ |
| ^(20, 38)^ |
| ^(52)^ |
| ^(105)^ |
| ^(34, 36)^ |
| ^(18)^ |
| ^(21)^ |
| ^-^ |
| ^(34, 43, 45);PathCards^ |
| ^(31)^ |
| ^(52)^ |
| ^(19)^ |
| ^(106)^ |
| ^(52, 107, 108)^ |
| ^(1)^ |
| ^(1, 13, 24, 52)^ |
| ^(16)^ |
| ^(21)^ |
| ^(31)^ |

**Table S7. Priori functional candidate genes.**

Genes presented in this table are likely to be involved in high-altitude adaptation based on categories provided in the first column. On one hand, we integrated genes associated with hypoxia-related pathways and functions (see Fig. 1D) from the PathCards Database (http://pathcards.genecards.org). “HIF pathway” is an integrated category of “HIF-1 signaling pathway”, “HIF repressor pathway”, “HIF1α transduction factor network”, “HIF2α transduction factor network” and “Hypoxic and oxygen homeostasis regulation of HIF1α”; “TCA” is an integrated category of “Citrate cycle” and “Pyruvate metabolism and citric acid cycle”; “DNA damage and repair” is an integrated category of “DNA damage” and “DNA double-strand break repair”. On the other hand, we collected genes identified in previous studies (references are listed below). These genes have been either experimentally confirmed to be involved in the responses to hypoxia, or identified in population genomic studies on high-altitude human or other animal groups. Potential candidate genes identified in the mitochondrial genome and on the X chromosome were not considered for this study. References for the “prior literatures”: (6, 8-13, 15, 17, 22, 26, 35, 54, 56-58, 62, 103, 106, 109-150)

**Table S8. Summary of the 62 quantitative traits in Tibetans.**

| **Trait name** | **Description** | **Men (n=1,064)** | |  | **Women (n=1,785)** | |
| --- | --- | --- | --- | --- | --- | --- |
|  |  | **Mean** | **SD** |  | **Mean** | **SD** |
| AVSLPTIM | Average sleep time per day | 7.67 | 1.87 |  | 7.54 | 1.67 |
| HEIGHT | Height | 166.68 | 7.44 |  | 155.76 | 6.86 |
| WEIGHT | Weight | 69.38 | 12.12 |  | 60.08 | 10.42 |
| BMI | Body mass index | 24.62 | 4.17 |  | 24.42 | 3.97 |
| SBP | Systolic blood pressure | 126.91 | 21.3 |  | 118.48 | 22.75 |
| DBP | Diastolic blood pressure | 76.1 | 13.94 |  | 73.31 | 13.17 |
| XL | Heart rate | 81.95 | 14.15 |  | 81.72 | 12.5 |
| FT3 | Free triiodothyronine | 5.67 | 1.18 |  | 5.38 | 1.94 |
| FT4 | Free thyroxine | 18.47 | 3.57 |  | 18.9 | 8.01 |
| TSH | Thyroid Stimulating Hormone | 3.47 | 3.98 |  | 3.71 | 5.17 |
| PTH | Parathyroid Hormone | 28.57 | 19.53 |  | 33.44 | 21.81 |
| B12 | Vitamin B12 | 473.3 | 261.62 |  | 515 | 284.99 |
| FOLATE | Folate | 6.29 | 2.41 |  | 7.36 | 2.71 |
| ALT | Glutamate pyruvate transaminase | 27.45 | 28.11 |  | 18.41 | 17.38 |
| AST | Glutamic oxalacetic transaminase | 25.78 | 12.92 |  | 22.65 | 12.95 |
| AST2ALT | AST/ALT ratio | 1.2 | 0.65 |  | 1.48 | 0.88 |
| TP | Total protein | 77.2 | 5.08 |  | 77.35 | 5.36 |
| ALB | Albumin | 47.62 | 3.35 |  | 46.85 | 2.85 |
| GLB | Globulin | 29.55 | 4.17 |  | 30.45 | 4.69 |
| A2G | ALB/GLB ratio | 1.72 | 1.57 |  | 1.64 | 1.38 |
| TBIL | Total bilirubin | 13.33 | 7.48 |  | 10.38 | 6.96 |
| DBIL | Direct bilirubin | 5.73 | 2.65 |  | 4.54 | 3.11 |
| IBIL | Indirect bilirubin | 7.63 | 5.16 |  | 5.89 | 4.84 |
| ALP | Alkaline phosphatase | 94.7 | 35.22 |  | 85.05 | 30.95 |
| GGT | Gamma-glutamyl transpeptidase | 49.25 | 50.19 |  | 29.98 | 32.67 |
| GLU | Glucose | 5.43 | 1.79 |  | 5.04 | 1.03 |
| UREA | Urea nitrogen | 4.83 | 1.79 |  | 4.56 | 1.7 |
| CRE | Creatinine | 76.68 | 14.81 |  | 59.44 | 10.86 |
| UA | Uric acid | 394.93 | 82.99 |  | 298.1 | 73.26 |
| TG | Triglyceride | 1.18 | 0.66 |  | 1.03 | 0.47 |
| TCH | Total cholesterol | 4.6 | 1.08 |  | 4.51 | 1.02 |
| HDL | High density lipoprotein | 1.2 | 0.23 |  | 1.35 | 0.28 |
| LDL | Low density lipoprotein | 2.89 | 0.86 |  | 2.61 | 0.76 |
| K | Potassium | 4.3 | 0.47 |  | 4.35 | 0.45 |
| NA. | Sodium | 140.93 | 3.9 |  | 140.39 | 3.32 |
| CL | Aluminium | 106.85 | 3.34 |  | 107.16 | 2.83 |
| CA | Calcium | 2.39 | 0.13 |  | 2.34 | 0.12 |
| PHOS | Phosphorus | 1.14 | 0.19 |  | 1.22 | 0.16 |
| FE | Ferrum | 18.89 | 8.94 |  | 14.98 | 8.55 |
| FER | Ferrtin | 207.85 | 185.3 |  | 83.36 | 117.91 |
| *(Continued)* | | | | | | |
| HCY | Homocysteine | 27.17 | 24.15 |  | 18.64 | 7.86 |
| HBA1C | Glucosylated hemoglobin | 5.46 | 0.83 |  | 4.92 | 0.68 |
| WBC | White blood cell count | 6.18 | 1.89 |  | 6.19 | 1.87 |
| LYMPH | Lymphocyte count | 1.87 | 0.62 |  | 1.92 | 0.67 |
| MID1 | Intermediate cell count | 0.35 | 0.15 |  | 0.33 | 0.19 |
| GRAN1 | Neutrophile granulocyte | 3.96 | 1.63 |  | 3.94 | 1.6 |
| LYMPH1 | Lymphocyte percentage | 31.4 | 8.84 |  | 32.13 | 9.54 |
| MID2 | Intermediate cell percentage | 5.94 | 2.11 |  | 5.67 | 2.93 |
| GRAN2 | Neutrophile granulocyte percentage | 62.66 | 9.07 |  | 62.2 | 10.25 |
| RBC | Red blood cell count | 5.19 | 0.73 |  | 4.75 | 0.64 |
| HGB | Hemoglobin | 171.09 | 25.87 |  | 149.92 | 26.01 |
| MCHC | Mean corpuscular hemoglobin concentration | 349.23 | 18.05 |  | 343.59 | 13.19 |
| MCV | Mean corpuscular volume | 94.63 | 5.94 |  | 91.77 | 8.21 |
| MCH | Mean corpuscular hemoglobin | 33.04 | 2.98 |  | 31.55 | 3.55 |
| RDW_CV | Coefficient of variation of red blood cell | 14.64 | 1.26 |  | 14.78 | 1.67 |
| HCT | Hematocrit | 49.04 | 7.41 |  | 43.48 | 6.85 |
| PLT | Platelets | 213.05 | 57.49 |  | 252.74 | 75.11 |
| MPV | Mean platelet volume | 8.33 | 0.87 |  | 8.51 | 0.91 |
| PDW | Platelet distribution width | 15.97 | 0.3 |  | 15.9 | 0.31 |
| PCT | Thrombocytocrit | 0.18 | 0.04 |  | 0.21 | 0.05 |
| RDW_SD | Standard deviation of red blood cell distribution | 49.91 | 4.28 |  | 48.66 | 4.48 |
| VITD | Vitamin D | 19.75 | 7.58 |  | 17.05 | 5.99 |

This table was extracted from Table S5 in Yang et al. (56)

**Table S9. Significant associations between the candidate AGVs and traits identified by the linear model.**

| **CHR** | **ID** | **POS** | **Nearest Gene** | **Traits** | | | | | | | | | |
| --- | --- | --- | --- | --- | --- | --- | --- | --- | --- | --- | --- | --- | --- |
|  |  |  |  | **B12** | **FOLATE** | **ALP** | **GGT** | **UA** | **PHOS** | **HCY** | **RBC** | **HGB** | **HCT** |
| 1 | rs198383 | 11933640 | *RP5-934G17.6* | - | - | - | - | - | - | 1.85e-4 | - | - | - |
| 1 | rs41278638 | 12072722 | *MFN2* | - | 0.035 | - | - | - | - | - | - | - | - |
| 1 | rs2295283 | 12082926 | *MIIP* | - | - | - | - | - | - | 0.025 | - | - | - |
| 1 | rs2180179 | 12095030 | *RN7SL649P* | - | 0.003 | - | - | - | - | 0.005 | - | - | - |
| 2 | rs309272 | 7202101 | *RNF144A* | - | - | 0.015 | - | - | - | - | - | - | - |
| 2 | rs149594770 | 46552202 | *EPAS1* | - | - | - | - | 0.011 | - | - | 0.008 | 0.045 | 0.004 |
| 2 | rs375418933 | 46558530 | *EPAS1* | - | - | - | - | 0.002 | - | - | 0.005 | 0.019 | 0.001 |
| 2 | rs1562453 | 46580474 | *EPAS1* | - | - | - | - | 0.002 | - | - | 0.001 | 0.020 | 0.004 |
| 2 | rs7599883 | 46581544 | *EPAS1* | - | - | - | - | 0.007 | - | - | - | - | - |
| 2 | rs141366568 | 46594122 | *EPAS1* | - | - | - | - | 1.89e-5 | - | - | 8.19e-6 | 8.55e-5 | 4.55e-6 |
| 2 | rs4953361 | 46598568 | *EPAS1* | - | - | - | - | 0.029 | - | - | 0.017 | - | - |
| 2 | rs369097672 | 46600358 | *EPAS1* | - | - | - | - | 3.11e-5 | - | - | 7.78e-6 | 1.84e-4 | 9.30e-6 |
| 2 | rs3088359 | 46602251 | *EPAS1* | - | - | - | - | 2.32e-4 | - | - | 4.55e-6 | 0.002 | 1.78e-4 |
| 2 | rs1900592 | 46629693 | *EPAS1* | - | - | - | - | 0.005 | - | - | 8.48e-6 | 0.002 | 1.00e-4 |
| 2 | rs72618627 | 46665512 | *TMEM247* | - | - | - | - | - | - | - | - | - | 0.038 |
| 2 | rs1868079 | 46686533 | *TMEM247* | - | - | - | - | 0.006 | - | - | 5.14e-7 | 5.68e-5 | 2.50e-6 |
| 2 | rs7595513 | 46687867 | *TMEM247* | - | - | - | - | - | - | - | - | - | 0.039 |
| 2 | rs57720200 | 46688955 | *TMEM247* | - | - | - | - | - | - | - | 0.003 | - | - |
| 2 | rs112416191 | 46691903 | *TMEM247* | - | - | - | - | 0.006 | - | - | 3.10e-7 | 3.58e-5 | 1.56e-6 |
| 2 | rs116871724 | 46693993 | *TMEM247* | - | - | - | - | 0.006 | - | - | 3.10e-7 | 2.90e-5 | 1.37e-6 |
| 2 | rs3814047 | 46706618 | *TMEM247* | - | - | - | - | - | - | - | 0.002 | - | 0.046 |
| 2 | rs192690066 | 46706765 | *TMEM247* | - | - | - | - | - | - | - | 0.008 | - | - |
| 2 | rs116983452 | 46707674 | *TMEM247* | - | - | - | - | 0.025 | - | - | 5.64e-7 | 4.90e-5 | 2.22e-6 |
| 2 | rs79542054 | 46710530 | *TMEM247* | - | - | - | - | 0.005 | - | - | 3.10e-7 | 3.40e-5 | 1.37e-6 |
| 2 | rs13018477 | 46710568 | *TMEM247* | - | - | - | - | - | - | - | 0.001 | - | 0.025 |
| 2 | rs12473626 | 46716828 | *ATP6V1E2* | - | - | - | - | - | - | - | 0.005 | - | - |
| 2 | rs12986653 | 46718090 | *ATP6V1E2* | - | - | - | - | 0.010 | - | - | 3.10e-7 | 3.58e-5 | 1.21e-6 |
| 2 | rs67739992 | 46728925 | *ATP6V1E2* | - | - | - | - | - | - | - | - | - | 0.026 |
| 2 | rs79117809 | 46729845 | *ATP6V1E2* | - | - | - | - | 0.023 | - | - | 3.10e-7 | 9.99e-5 | 3.20e-6 |
| 2 | rs11125079 | 46732215 | *ATP6V1E2* | - | - | - | - | 0.003 | - | - | 0.005 | 0.007 | 0.001 |
| 2 | rs117128262 | 46733738 | *ATP6V1E2* | - | - | - | - | 0.046 | - | - | 3.10e-7 | 1.18e-4 | 3.36e-6 |
| 2 | rs13024546 | 46734325 | *ATP6V1E2* | - | - | - | - | 0.027 | - | - | 3.10e-7 | 9.99e-5 | 2.22e-6 |
| 2 | rs2346415 | 46745452 | *ATP6V1E2* | - | - | - | - | - | - | - | 1.37e-6 | 0.001 | 2.84e-5 |
| 2 | rs6544900 | 46752101 | *ATP6V1E2* | - | - | - | - | - | - | - | 0.045 | - | - |
| 2 | rs78082841 | 46752292 | *ATP6V1E2* | - | - | - | - | 0.045 | - | - | 4.24e-6 | 0.001 | 8.51e-5 |
| 2 | rs11676473 | 46762596 | *ATP6V1E2* | - | - | - | - | - | - | - | 0.002 | 0.020 | 0.004 |
| 2 | rs60604300 | 46767692 | *ATP6V1E2* | - | - | - | - | - | - | - | 0.001 | 0.003 | 7.15e-4 |
| 2 | rs75498296 | 46768078 | *ATP6V1E2* | - | - | - | - | 0.029 | - | - | 2.17e-6 | 9.22e-4 | 7.37e-5 |
| 2 | rs1001746 | 46774599 | *RHOQ* | - | - | - | - | - | - | - | 0.001 | 0.010 | 0.001 |
| 2 | rs17818399 | 46826026 | *PIGF* | - | - | - | - | - | - | - | 0.002 | 0.041 | 0.005 |
| 2 | rs13000706 | 46838247 | *PIGF* | - | - | - | - | - | - | - | 0.004 | 0.026 | 0.002 |
| 2 | rs118024480 | 46840552 | *PIGF* | - | - | - | - | - | - | - | 3.72e-5 | 0.006 | 6.46e-4 |
| *(Continued)* | | | | | | | | | | | | | |
| 2 | rs77297964 | 46844352 | *CRIPT* | - | - | - | - | - | - | - | 4.20e-5 | 0.006 | 6.22e-4 |
| 2 | rs2346419 | 46845069 | *CRIPT* | - | - | - | - | 0.019 | - | - | 5.94e-5 | 0.003 | 1.12e-4 |
| 2 | rs3087822 | 46852033 | *CRIPT* | - | - | - | - | - | - | - | 0.004 | 0.016 | 0.001 |
| 2 | rs1080871 | 46854708 | *CRIPT* | - | - | - | - | - | - | - | 0.004 | 0.011 | 7.84e-4 |
| 2 | rs7598578 | 46857085 | *CRIPT* | - | - | - | - | - | - | - | 0.010 | 0.032 | 0.002 |
| 2 | rs12104572 | 46862466 | *CRIPT* | - | - | - | - | - | - | - | 0.007 | 0.024 | 0.001 |
| 2 | rs12105006 | 46862586 | *CRIPT* | - | - | - | - | - | - | - | 4.97e-4 | 0.020 | 7.82e-4 |
| 2 | rs75553031 | 46863851 | *CRIPT* | - | - | - | - | - | - | - | 8.55e-5 | 0.010 | 0.001 |
| 2 | rs6544906 | 46863872 | *CRIPT* | - | - | - | - | - | - | - | 0.008 | 0.023 | 0.001 |
| 2 | rs13385693 | 46867158 | *CRIPT* | - | - | - | - | - | - | - | 0.010 | 0.026 | 0.001 |
| 2 | rs6544907 | 46868319 | *CRIPT* | - | - | - | - | - | - | - | 0.006 | 0.016 | 9.22e-4 |
| 2 | rs7599097 | 46874040 | *CRIPT* | - | - | - | - | - | - | - | 0.001 | - | 0.025 |
| 2 | rs10495934 | 46874746 | *CRIPT* | - | - | - | - | - | - | - | 0.008 | - | 0.022 |
| 2 | rs117611189 | 46878865 | *CRIPT* | - | - | - | - | - | - | - | 0.006 | - | - |
| 2 | rs10179861 | 46883202 | *CRIPT* | - | - | - | - | - | - | - | 2.17e-5 | 0.001 | 7.92E-05 |
| 2 | rs4953403 | 46885394 | *CRIPT* | - | - | - | - | - | - | - | 0.004 | - | - |
| 2 | rs4953408 | 46886565 | *CRIPT* | - | - | - | - | 0.024 | - | - | 1.25e-5 | 0.005 | 0.000617502 |
| 2 | rs12328738 | 46889746 | *SOCS5* | - | - | - | - | 0.024 | - | - | 1.26e-5 | 0.010 | 0.001 |
| 2 | rs11125086 | 46889996 | *SOCS5* | - | - | - | - | - | - | - | 0.005 | - | - |
| 2 | rs10209278 | 46891964 | *SOCS5* | - | - | - | - | - | - | - | 5.45e-4 | 0.007 | 0.002 |
| 2 | rs1869838 | 46899285 | *SOCS5* | - | - | - | - | - | - | - | 7.27e-4 | 0.007 | 0.003 |
| 2 | rs3814041 | 46908571 | *SOCS5* | - | - | - | - | - | - | - | 5.62e-4 | 0.007 | 0.003 |
| 2 | rs28370327 | 46910064 | *SOCS5* | - | - | - | - | - | - | - | 1.60e-4 | 0.002 | 6.22e-4 |
| 2 | rs73926564 | 46910488 | *SOCS5* | - | - | - | - | - | - | - | 5.96e-4 | 0.005 | 0.001 |
| 14 | rs61392826 | 73313188 | *DPF3* | 0.033 | - | - | - | - | - | - | - | - | - |
| 17 | rs1495099 | 37784464 | *PPP1R1B* | - | - | - | - | - | 0.026 | - | - | - | - |
| 19 | rs2446206 | 5777415 | *CATSPERD* | - | - | - | 0.018 | - | - | - | - | - | - |
| 19 | rs2305925 | 5778517 | *CATSPERD* | - | - | - | 0.045 | - | - | - | - | - | - |
| 19 | rs8106226 | 5781242 | *PRR22* | - | - | - | 0.038 | - | - | - | - | - | - |
| 19 | rs3745640 | 5783905 | *PRR22* | - | - | - | 0.024 | - | - | - | - | - | - |
| 19 | rs10811 | 5786815 | *DUS3L* | - | - | - | 0.046 | - | - | - | - | - | - |

The associations between 1,865 candidate AGVs and 62 quantitative traits (Table S8) were tested under a linear regression model (see Methods). Here listed the significant adjusted *p* values (< 0.05; red: positive association with the adaptive allele; green: negative association with the adaptive allele). Candidate AGVs did not show significant association with any trait and those traits which were not associated with any candidate AGV are not listed in this table. The short line indicates non-significant associations (adjusted *p* > 0.05). These 10 traits were reported to be HAA-related in literatures: B12, (151-153); FOLATE, (154-157); ALP, (158-161); GGT, (162-165); UA, (166-169); PHOS, (170-173); HCY, (174-177); RBC, (178-181); HGB, (182-185); HCT, (186-189).

**Table S10. Significant associations between the candidate AGVs and traits identified using MLMA-LOCO.**

| **CHR** | **ID** | | | **POS** | **Nearest Gene** | **Traits** | | | | | | | | | | | |
| --- | --- | --- | --- | --- | --- | --- | --- | --- | --- | --- | --- | --- | --- | --- | --- | --- | --- |
|  |  |  |  |  |  | **HEIGHT** | **B12** | **FOLATE** | **ALP** | **GGT** | **CRE** | **UA** | **HCY** | **RBC** | **HGB** | **HCT** | **RDW_SD** |
| 1 | rs198383 | | | 11933640 | *RP5-934G17.6* | - | - | - | - | - | - | - | 8.24e-5 | - | - | - | - |
| 1 | rs41278638 | | | 12072722 | *MFN2* | - | - | 0.014 | - | - | - | - | - | - | - | - | - |
| 1 | rs2295283 | | | 12082926 | *MIIP* | - | - | - | - | - | - | - | 0.011 | - | - | - | - |
| 1 | rs2180179 | | | 12095030 | *RN7SL649P* | - | - | 0.003 | - | - | - | - | 0.006 | - | - | - | - |
| 2 | rs309272 | | | 7202101 | *RNF144A* | - | - | - | 0.009 | - | - | - | - | - | - | - | - |
| 2 | rs10865087 | | | 31324098 | *GALNT14* | - | - | - | - | - | 0.046 | - | - | - | - | - | - |
| 2 | rs280696 | | | 35075640 | *AC012593.1* | 0.035 | - | - | - | - | - | - | - | - | - | - | - |
| 2 | rs149594770 | | | 46552202 | *EPAS1* | - | - | - | - | - | - | 0.031 | - | 0.024 | - | 0.015 | - |
| 2 | rs375418933 | | | 46558530 | *EPAS1* | - | - | - | - | - | - | 0.007 | - | 0.015 | 0.042 | 0.005 | - |
| 2 | rs1562453 | | | 46580474 | *EPAS1* | - | - | - | - | - | - | 7.20e-4 | - | 0.002 | 0.027 | 0.006 | - |
| 2 | rs7599883 | | | 46581544 | *EPAS1* | - | - | - | - | - | - | 0.002 | - | - | - | - | - |
| 2 | rs141366568 | | | 46594122 | *EPAS1* | - | - | - | - | - | - | 3.74e-5 | - | 1.72e-5 | 2.30e-4 | 1.45e-5 | - |
| 2 | rs4953361 | | | 46598568 | *EPAS1* | - | - | - | - | - | - | 0.002 | - | 0.008 | - | - | - |
| 2 | rs369097672 | | | 46600358 | *EPAS1* | - | - | - | - | - | - | 9.89e-5 | - | 3.74e-5 | 9.14e-4 | 7.07e-5 | - |
| 2 | rs3088359 | | | 46602251 | *EPAS1* | - | - | - | - | - | - | 3.06e-5 | - | 9.25e-6 | 0.002 | 2.30e-4 | - |
| 2 | rs1900592 | | | 46629693 | *EPAS1* | - | - | - | - | - | - | 6.05e-4 | - | 6.62e-6 | 8.18e-4 | 6.31e-5 | - |
| 2 | rs72618627 | | | 46665512 | *TMEM247* | - | - | - | - | - | - | - | - | - | - | 0.009 | - |
| 2 | rs1868079 | | | 46686533 | *TMEM247* | - | - | - | - | - | - | 0.020 | - | 2.42e-6 | 2.30e-4 | 1.36e-5 | - |
| 2 | rs7595513 | | | 46687867 | *TMEM247* | - | - | - | - | - | - | 0.034 | - | - | 0.044 | 0.008 | 0.041 |
| 2 | rs57720200 | | | 46688955 | *TMEM247* | - | - | - | - | - | - | - | - | 0.014 | - | - | - |
| 2 | rs112416191 | | | 46691903 | *TMEM247* | - | - | - | - | - | - | 0.017 | - | 1.61e-6 | 1.39e-4 | 8.63e-6 | - |
| 2 | rs116871724 | | | 46693993 | *TMEM247* | - | - | - | - | - | - | 0.014 | - | 1.59e-6 | 8.63e-5 | 6.62e-6 | - |
| 2 | rs3814047 | | | 46706618 | *TMEM247* | - | - | - | - | - | - | - | - | 0.020 | - | - | - |
| 2 | rs192690066 | | | 46706765 | *TMEM247* | - | - | - | - | - | - | - | - | 0.009 | - | - | - |
| 2 | rs116983452 | | | 46707674 | *TMEM247* | - | - | - | - | - | - | - | - | 2.44e-6 | 1.46e-4 | 8.78e-6 | - |
| 2 | rs12612916 | | | 46707886 | *TMEM247* | - | - | - | - | - | - | 0.038 | - | - | - | 0.014 | - |
| 2 | rs79542054 | | | 46710530 | *TMEM247* | - | - | - | - | - | - | 0.015 | - | 1.61e-6 | 1.39e-4 | 8.19e-6 | - |
| 2 | rs13018477 | | | 46710568 | *TMEM247* | - | - | - | - | - | - | - | - | 0.014 | - | - | - |
| 2 | rs12473626 | | | 46716828 | *ATP6V1E2* | - | - | - | - | - | - | - | - | 0.039 | - | - | - |
| 2 | rs12986653 | | | 46718090 | *ATP6V1E2* | - | - | - | - | - | - | - | - | 2.42e-6 | 2.98e-4 | 9.25e-6 | - |
| 2 | rs1551123 | | | 46721331 | *ATP6V1E2* | - | - | - | - | - | - | 0.013 | - | - | - | 0.011 | - |
| 2 | rs67739992 | | | 46728925 | *ATP6V1E2* | - | - | - | - | - | - | 0.014 | - | 0.027 | 0.023 | 0.004 | - |
| 2 | rs79117809 | | | 46729845 | *ATP6V1E2* | - | - | - | - | - | - | - | - | 1.61e-6 | 4.23e-4 | 1.64e-5 | - |
| 2 | rs11125079 | | | 46732215 | *ATP6V1E2* | - | - | - | - | - | - | 4.95e-4 | - | 0.004 | 0.003 | 4.75e-4 | - |
| 2 | rs117128262 | | | 46733738 | *ATP6V1E2* | - | - | - | - | - | - | - | - | 1.59e-6 | 4.64e-4 | 1.64e-5 | - |
| 2 | rs13024546 | | | 46734325 | *ATP6V1E2* | - | - | - | - | - | - | - | - | 1.59e-6 | 4.75e-4 | 1.45e-5 | - |
| 2 | rs2346415 | | | 46745452 | *ATP6V1E2* | - | - | - | - | - | - | - | - | 9.25e-6 | 0.005 | 2.24e-4 | - |
| 2 | rs6544900 | | | 46752101 | *ATP6V1E2* | - | - | - | - | - | - | - | - | 0.020 | - | - | - |
| 2 | rs78082841 | | | 46752292 | *ATP6V1E2* | - | - | - | - | - | - | - | - | 1.79e-5 | 0.004 | 3.50e-4 | - |
| 2 | rs11676473 | | | 46762596 | *ATP6V1E2* | - | - | - | - | - | - | - | - | 0.002 | 0.015 | 0.003 | - |
| 2 | rs60604300 | | | 46767692 | *ATP6V1E2* | - | - | - | - | - | - | - | - | 0.007 | 0.015 | 0.004 | - |
| 2 | rs75498296 | | | 46768078 | *ATP6V1E2* | - | - | - | - | - | - | - | - | 8.63e-6 | 0.003 | 3.10e-4 | - |
|  | |  | *(Continued)* | | | | | | | | | | | | | | |
| 2 | rs1001746 | | | 46774599 | *RHOQ* | - | - | - | - | - | - | - | - | 0.003 | 0.016 | 0.002 | - |
| 2 | rs17818399 | | | 46826026 | *PIGF* | - | - | - | - | - | - | - | - | 0.003 | - | 0.008 | - |
| 2 | rs13000706 | | | 46838247 | *PIGF* | - | - | - | - | - | - | - | - | 0.004 | 0.015 | 0.001 | - |
| 2 | rs118024480 | | | 46840552 | *PIGF* | - | - | - | - | - | - | - | - | 8.52e-5 | 0.011 | 0.001 | - |
| 2 | rs77297964 | | | 46844352 | *CRIPT* | - | - | - | - | - | - | - | - | 8.24e-5 | 0.008 | 8.92e-4 | - |
| 2 | rs2346419 | | | 46845069 | *CRIPT* | - | - | - | - | - | - | 0.007 | - | 3.74e-5 | 0.002 | 6.29e-5 | - |
| 2 | rs3087822 | | | 46852033 | *CRIPT* | - | - | - | - | - | - | - | - | 0.003 | 0.008 | 4.85e-4 | - |
| 2 | rs1080871 | | | 46854708 | *CRIPT* | - | - | - | - | - | - | - | - | 0.004 | 0.008 | 5.57e-4 | - |
| 2 | rs7598578 | | | 46857085 | *CRIPT* | - | - | - | - | - | - | - | - | 0.009 | 0.020 | 0.001 | - |
| 2 | rs12104572 | | | 46862466 | *CRIPT* | - | - | - | - | - | - | - | - | 0.006 | 0.014 | 8.92e-4 | - |
| 2 | rs12105006 | | | 46862586 | *CRIPT* | - | - | - | - | - | - | 0.027 | - | 4.04e-4 | 0.012 | 4.75e-4 | - |
| 2 | rs75553031 | | | 46863851 | *CRIPT* | - | - | - | - | - | - | - | - | 2.18e-4 | 0.019 | 0.002 | - |
| 2 | rs6544906 | | | 46863872 | *CRIPT* | - | - | - | - | - | - | - | - | 0.008 | 0.013 | 7.64e-4 | - |
| 2 | rs13385693 | | | 46867158 | *CRIPT* | - | - | - | - | - | - | - | - | 0.009 | 0.015 | 8.92e-4 | - |
| 2 | rs6544907 | | | 46868319 | *CRIPT* | - | - | - | - | - | - | - | - | 0.005 | 0.009 | 4.75e-4 | - |
| 2 | rs7599097 | | | 46874040 | *CRIPT* | - | - | - | - | - | - | - | - | 0.001 | - | 0.020 | - |
| 2 | rs10495934 | | | 46874746 | *CRIPT* | - | - | - | - | - | - | - | - | 0.004 | - | 0.006 | - |
| 2 | rs117611189 | | | 46878865 | *CRIPT* | - | - | - | - | - | - | - | - | 0.014 | - | - | - |
| 2 | rs10179861 | | | 46883202 | *CRIPT* | - | - | - | - | - | - | - | - | 1.68e-5 | 0.001 | 7.62e-5 | - |
| 2 | rs4953403 | | | 46885394 | *CRIPT* | - | - | - | - | - | - | - | - | 0.031 | - | - | - |
| 2 | rs4953408 | | | 46886565 | *CRIPT* | - | - | - | - | - | - | 0.006 | - | 6.62e-6 | 0.002 | 2.18e-4 | - |
| 2 | rs12328738 | | | 46889746 | *SOCS5* | - | - | - | - | - | - | 0.008 | - | 7.90e-6 | 0.005 | 4.64e-4 | - |
| 2 | rs11125086 | | | 46889996 | *SOCS5* | - | - | - | - | - | - | - | - | 0.020 | - | - | - |
| 2 | rs6742593 | | | 46890538 | *SOCS5* | - | - | - | - | - | - | - | - | 0.020 | - | - | - |
| 2 | rs10209278 | | | 46891964 | *SOCS5* | - | - | - | - | - | - | - | - | 3.50e-4 | 0.004 | 0.001 | - |
| 2 | rs1869838 | | | 46899285 | *SOCS5* | - | - | - | - | - | - | - | - | 8.92e-4 | 0.008 | 0.003 | - |
| 2 | rs3814041 | | | 46908571 | *SOCS5* | - | - | - | - | - | - | - | - | 6.10e-4 | 0.009 | 0.003 | - |
| 2 | rs28370327 | | | 46910064 | *SOCS5* | - | - | - | - | - | - | - | - | 4.04e-4 | 0.005 | 0.002 | - |
| 2 | rs73926564 | | | 46910488 | *SOCS5* | - | - | - | - | - | - | - | - | 7.64e-4 | 0.008 | 0.002 | - |
| 6 | rs868155 | | | 122389906 | *RP11-284G10.1* | 0.034 | - | - | - | - | - | - | - | - | - | - | - |
| 9 | rs79678337 | | | 118418579 | *RP11-284G10.1* | - | - | - | - | - | 0.020 | - | - | - | - | - | - |
| 9 | rs10982861 | | | 118425237 | *RP11-284G10.1* | - | - | - | - | - | 0.007 | - | - | - | - | - | - |
| 9 | rs10817815 | | | 118451020 | *RP11-284G10.1* | 0.043 | - | - | - | - | - | - | - | - | - | - | - |
| 10 | rs144747795 | | | 24924930 | *ARHGAP21* | 0.046 | - | - | - | - | - | - | - | - | - | - | - |
| 11 | rs12420466 | | | 100829892 | *ARHGAP42* | 0.033 | - | - | - | - | - | - | - | - | - | - | - |
| 14 | rs61392826 | | | 73313188 | *DPF3* | - | 0.019 | - | - | - | - | - | - | - | - | - | - |
| 19 | rs2436493 | | | 5777323 | *CATSPERD* | - | - | - | - | 0.020 | - | - | - | - | - | - | - |
| 19 | rs2446206 | | | 5777415 | *CATSPERD* | - | - | - | - | 0.011 | - | - | - | - | - | - | - |
| 19 | rs2305925 | | | 5778517 | *CATSPERD* | - | - | - | - | 0.014 | - | - | - | - | - | - | - |
| 19 | rs8106226 | | | 5781242 | *PRR22* | - | - | - | - | 0.019 | - | - | - | - | - | - | - |
| 19 | rs3745640 | | | 5783905 | *PRR22* | - | - | - | - | 0.009 | - | - | - | - | - | - | - |
| 19 | rs10811 | | | 5786815 | *DUS3L* | - | - | - | - | 0.014 | - | - | - | - | - | - | - |
| 19 | rs274145 | | | 56675827 | *ZNF444* | 0.022 | - | - | - | - | - | - | - | - | - | - | - |

The associations between 1,865 candidate AGVs and 62 quantitative traits (Table S8) were tested under a mixed linear model (see Methods). Here listed the significant adjusted *p*-values (< 0.05; red: positive association with the adaptive allele; green: negative association with the adaptive allele). Candidate AGVs did not show significant association with any trait and those traits which were not associated with any candidate AGV are not listed in this table. The short line indicates non-significant associations (adjusted *p* > 0.05).

**Table S11. A list of eQTLs identified in the candidate AGVs in the Tibetans.**

| ***cis*-associated**  **gene** | **AGV** | **Up/Down-**  **regulation** | **Adjusted**  ***p*-value** | ***cis*-associated**  **gene** | **AGV** | **Up/Down-**  **regulation** | **Adjusted**  ***p*-value** |
| --- | --- | --- | --- | --- | --- | --- | --- |
| *SMUG1* | rs2233921 | ↑ | 0.45×10^-10^ | *TMEM247* | rs116871724 | ↓ | 0.001 |
|  | rs3087404 | ↑ | 0.37×10^-19^ |  | rs7595513 | ↑ | 0.015 |
|  | rs3136384 | ↑ | 0.30×10^-5^ |  | rs1868079 | ↓ | 0.022 |
| *ZFAND2A* | rs2949193 | ↓ | 0.80×10^-5^ |  | rs79542054 | ↓ | 0.034 |
|  | rs6946684 | ↓ | 0.13×10^-4^ |  | rs116983452 | ↓ | 0.038 |
|  | rs2949204 | ↓ | 0.17×10^-4^ | *PGAP3* | rs2941504 | ↓ | 0.002 |
|  | rs6463499 | ↓ | 0.93×10^-4^ |  | rs14050 | ↓ | 0.002 |
|  | rs2960837 | ↓ | 0.23×10^-3^ |  | rs1877031 | ↓ | 0.002 |
|  | rs2949196 | ↓ | 0.56×10^-3^ |  | rs8077172 | ↓ | 0.012 |
|  | rs2960830 | ↓ | 0.043 |  | rs1053651 | ↓ | 0.012 |
| *RAB4B* | rs3865452 | ↓ | 0.65×10^-4^ |  | rs12150603 | ↓ | 0.023 |
| *DUS3L* | rs10811 | ↓ | 0.15×10^-3^ |  | rs1565922 | ↓ | 0.023 |
|  | rs3745640 | ↓ | 0.017 |  | rs903501 | ↓ | 0.030 |
| *AC091729.9* | rs2949196 | ↓ | 0.37×10^-3^ |  | rs2952152 | ↓ | 0.032 |
|  | rs6946684 | ↓ | 0.002 |  | rs903504 | ↓ | 0.034 |
|  | rs2949204 | ↓ | 0.003 |  | rs903503 | ↓ | 0.034 |
|  | rs2949193 | ↓ | 0.034 |  | rs2934956 | ↓ | 0.050 |
|  | rs6463499 | ↓ | 0.050 |  | rs1476278 | ↓ | 0.050 |
| *EPAS1* | rs3088359 | ↓ | 0.61×10^-3^ | *NISCH* | rs4282054 | ↑ | 0.007 |
|  | rs369097672 | ↓ | 0.84×10^-3^ |  | rs7636227 | ↑ | 0.039 |
|  | rs141366568 | ↓ | 0.84×10^-3^ | *RP11-834C11.3* | rs2233921 | ↑ | 0.007 |
|  | rs57720200 | ↓ | 0.002 |  | rs3087404 | ↑ | 0.014 |
|  | rs1868079 | ↓ | 0.019 | *TSC22D2* | rs2903834 | ↓ | 0.012 |
|  | rs3814047 | ↓ | 0.023 | *TRABD* | rs732405 | ↑ | 0.012 |
|  | rs116983452 | ↓ | 0.003 | *CTA-223H9.9* | rs1883828 | ↑ | 0.017 |
|  | rs1562453 | ↑ | 0.034 | *SEPT3* | rs2228313 | ↓ | 0.021 |
|  | rs4953348 | ↑ | 0.034 |  | rs17848337 | ↓ | 0.021 |
| *RP11-834C11.11* | rs3087404 | ↑ | 0.98×10^-4^ | *CCDC121* | rs1919129 | ↑ | 0.022 |
|  | rs2233921 | ↑ | 0.001 | *GNL3* | rs1029871 | ↓ | 0.034 |
|  | rs3136384 | ↑ | 0.050 |  | rs6617 | ↓ | 0.039 |
| *CSF2RB* | rs5750339 | ↓ | 0.98×10^-4^ |  | rs2289247 | ↓ | 0.039 |
|  | rs3788524 | ↓ | 0.017 |  | rs11177 | ↓ | 0.039 |
|  | rs2075939 | ↓ | 0.022 | *PALLD* | rs79454881 | ↑ | 0.034 |

Up/Down-regulation indicates the positive (red arrows) or negative (green arrows) correlation between the accumulation of the adaptive allele in TIB and the expression of gene.

**Table S12. Colocalization test for eQTLs and phenotype-associated candidate AGVs.**

The colocalization test for each gene-phenotype pair was performed using *coloc* version 3.1 (see Methods).

**Table S13. Effects of the candidate AGVs in *EPAS1* and those in *TMEM247* on the adaptive traits in Tibetans.**

The independent and interactive effects of candidate AGVs in *EPAS1* and those in *TMEM247* on red blood cell count (RBC), hemoglobin (HGB) and hematocrit (HCT) were tested based on three linear regression models (see Methods). Several representative candidates in *EPAS1* reported in previous studies, e.g., rs4953354 (190), rs372272284 (191), and rs149594770 (192), were also included in the analysis. Especially, rs149594770 was identified as one of the candidate AGVs in this study. The effect size of each variant and the genetic contribution of each model are shown in the table. Significant *p* values (*p* < 0.05) are highlighted with red fonts.

**Table S14. Selection coefficient estimated for rs116983452 in TIB.**

| **Model** | ***t*** | ***N_e_*** | ***p*_0_** | ***p*_t_** | ***s*** |
| --- | --- | --- | --- | --- | --- |
| 1 | [600, 360] | - | 0.026 | 0.763 | [0.0035, 0.0058] |
| 2 | [3240, 1256] | [2514, 2003] | 0.0002 | 0.763 | [0.0013, 0.0033] |
|  | [2436, 2212] | [1561, 1156] | [0.0003, 0.0004] | 0.763 | [0.0017, 0.0018] |

Abbreviations are defined as follows: *t*, selection time in generation assuming 25 years per generation; *p*_0_, initial frequency; *p*_t_, current frequency in TIB; *s*, selection coefficient in TIB. Model 1 assumes that the adaptation event occurred after the split of TIB and HAN, thus using the divergence time of these two populations (9,000 – 15,000 years ago, assuming 25 years per generation) as the duration of selection. Model 2 assumes that the adaptation event started right after the introgression of the T allele at rs116983452 from the archaic hominin to TIB, and considered the TMRCA calculated using two methods (see Methods) as the approximate of the selection time.

**Table S15. Validation of the association between rs116983452-T and hypoxia-related traits.**

| **Traits** | **Model** | **Association analysis** | | | |  | **Meta analysis** | |
| --- | --- | --- | --- | --- | --- | --- | --- | --- |
|  |  | **β** | ***p*** | **adjusted *p*** |  | | ***p*** | **adjusted *p*** |
| HGB | Additive | -0.422 | 0.808 | 0.836 |  | | 6.09 × 10^-4^ | 4.87 × 10^-3^ |
|  | Dominant | 3.023 | 0.181 | 0.512 |  | |  |  |
|  | Recessive | -1.887 | 0.577 | 0.727 |  | |  |  |
| RBC | Additive | 0.162 | 0.005 | 0.054 |  | | 0.034 | 0.181 |
|  | Dominant | -0.139 | 0.067 | 0.512 |  | |  |  |
|  | Recessive | 0.314 | 0.006 | 0.108 |  | |  |  |
| HCT | Additive | -0.001 | 0.805 | 0.836 |  | | 3.06 × 10^-4^ | 4.87 × 10^-3^ |
|  | Dominant | 0.006 | 0.311 | 0.537 |  | |  |  |
|  | Recessive | -0.005 | 0.637 | 0.727 |  | |  |  |

Significant adjusted *p* values are highlighted by the underlines.

**Table S16. Cross-conditional association analysis of *TMEM247* and *EPAS1*.**

| **SNP ID** | **UA** | **RBC** | **HGB** | **HCT** | **SNP ID** | **UA** | **RBC** | **HGB** | **HCT** | **SNP ID** | **UA** | **RBC** | **HGB** | **HCT** |
| --- | --- | --- | --- | --- | --- | --- | --- | --- | --- | --- | --- | --- | --- | --- |
| **rs149594770** | | | | | **rs4953361** | | | | |  | **rs7599883** | | | |
| rs1562453 | 0.017 | - | - | - | rs149594770 | 0.011 | 0.003 | 0.002 | 0.001 | rs149594770 | - | 0.001 | 0.035 | 0.012 |
| rs3088359 | 0.001 | 0.027 | - | - | rs375418933 | 0.005 | 0.004 | 0.002 | 0.001 | rs375418933 | - | 0.004 | - | 0.026 |
| rs1900592 | 0.011 | - | - | - | rs1562453 | 0.010 | 0.001 | 0.001 | 4.9e-4 | rs1562453 | - | 1.8e-4 | 0.004 | 0.001 |
| rs1868079 | 0.049 | - | - | - | rs141366568 | 0.001 | 1.3e-4 | 1.2e-4 | 6.4e-5 | rs141366568 | 0.020 | 2.6e-5 | 0.004 | 0.001 |
| rs112416191 | 0.049 | - | - | - | rs369097672 | 0.002 | 0.001 | 0.001 | 0.001 | rs4953361 | 0.024 | 0.027 | NA | NA |
| rs79542054 | 0.042 | - | - | - | rs3088359 | 0.007 | 2.9e-5 | 2.6e-4 | 1.3e-4 | rs369097672 | 0.018 | 2.3e-5 | 0.003 | 0.001 |
| **rs375418933** | | | | | rs1900592 | - | 0.002 | 0.002 | 0.002 | rs3088359 | 0.001 | 1.0e-5 | 0.004 | 0.001 |
| rs1562453 | 0.030 | - | - | - | rs1868079 | - | 3.3e-4 | 0.003 | 0.003 | rs1900592 | 0.022 | 0.002 | 0.030 | 0.017 |
| rs369097672 | - | 0.040 | - | - | rs57720200 | NA | 3.5e-4 | NA | NA | rs1868079 | 0.024 | 0.003 | 0.021 | 0.009 |
| rs3088359 | 0.001 | 0.010 | - | - | rs112416191 | - | 2.4e-4 | 0.003 | 0.002 | rs57720200 | NA | 0.001 | NA | NA |
| rs1900592 | 0.016 | 0.036 | - | - | rs116871724 | - | 1.8e-4 | 0.002 | 0.002 | rs112416191 | 0.021 | 0.003 | 0.021 | 0.009 |
| rs1868079 | 0.024 | - | - | - | rs3814047 | NA | 6.8e-5 | NA | 0.001 | rs116871724 | 0.021 | 0.003 | 0.020 | 0.008 |
| rs112416191 | 0.022 | - | - | - | rs116983452 | - | 0.001 | 0.008 | 0.008 | rs3814047 | NA | 4.6e-4 | NA | 0.001 |
| rs116871724 | 0.024 | - | - | - | rs79542054 | - | 3.0e-4 | 0.003 | 0.003 | rs192690066 | NA | 0.050 | NA | NA |
| rs116983452 | 0.042 | - | - | - | rs13018477 | NA | 3.1e-5 | NA | 0.001 | rs116983452 | 0.045 | 0.009 | 0.023 | 0.012 |
| rs79542054 | 0.022 | - | - | - | **rs1900592** | | | | | rs79542054 | 0.022 | 0.004 | 0.020 | 0.008 |
| **rs1562453** | | | | | rs141366568 | - | - | - | 0.047 | rs13018477 | NA | 4.8e-4 | NA | 0.001 |
| rs3088359 | 0.011 | 0.003 | - | 0.042 | rs3088359 | 0.043 | 0.011 | - | - |  | **rs1868079** | | | |
| rs3814047 | NA | 0.026 | NA | - | rs1868079 | - | 0.017 | 0.040 | 0.036 | rs149594770 | - | 0.024 | - | 0.023 |
| rs13018477 | NA | 0.034 | NA | - | rs57720200 | NA | 0.002 | NA | NA | rs375418933 | - | 0.048 | - | - |
| **rs369097672** | | | | | rs112416191 | - | 0.012 | 0.034 | 0.029 | rs1562453 | - | 0.008 | 0.034 | 0.014 |
| rs3088359 | 0.034 | 0.022 | - | - | rs116871724 | - | 0.010 | 0.031 | 0.027 | rs141366568 | - | 0.010 | - | 0.015 |
| **rs3088359** |  |  |  |  | rs3814047 | NA | 0.002 | NA | 0.007 | rs4953361 | 0.011 | - | NA | NA |
| rs141366568 | - | - | - | 0.042 | rs116983452 | - | 0.030 | 0.044 | 0.036 | rs369097672 | 0.023 | 0.014 | - | 0.022 |
| **rs141366568** |  |  |  |  | rs79542054 | - | 0.013 | 0.024 | 0.020 | rs3088359 | 0.002 | 0.001 | 0.022 | 0.008 |
| rs3088359 | 0.015 | - | - | - | rs13018477 | NA | 0.002 | NA | 0.004 | rs1900592 | 0.037 | - | - | - |
| **rs72618627** | | | | | **rs7595513** | | | | |  | **rs57720200** | | | |
| rs149594770 | 0.002 | 1.0e-4 | 0.001 | 9.6e-5 | rs149594770 | 0.001 | 6.9e-5 | 0.001 | 7.4e-5 | rs149594770 | 5.9e-5 | 3.5e-4 | 0.002 | 2.2e-4 |
| rs375418933 | 4.1e-4 | 7.3e-5 | 3.7e-4 | 6.2e-5 | rs375418933 | 3.2e-4 | 4.5e-5 | 3.2e-4 | 4.5e-5 | rs375418933 | 5.7e-5 | 4.9e-4 | 0.002 | 1.6e-4 |
| rs1562453 | 3.8e-4 | 2.7e-7 | 5.9e-5 | 8.4e-6 | rs1562453 | 4.0e-4 | 2.9e-7 | 6.0e-5 | 8.0e-6 | rs1562453 | 3.6e-5 | 2.5e-4 | 0.001 | 2.0e-4 |
| rs7599883 | 9.6e-4 | NA | NA | NA | rs7599883 | 0.001 | NA | NA | NA | rs7599883 | 1.3e-4 | NA | NA | NA |
| rs141366568 | 6.3e-6 | 1.2e-7 | 9.2e-6 | 1.1e-6 | rs141366568 | 4.9e-6 | 5.3e-8 | 6.3e-6 | 6.2e-7 | rs141366568 | 6.0e-7 | 6.5e-6 | 2.7e-5 | 1.7e-6 |
| rs4953361 | 0.002 | 0.004 | NA | NA | rs4953361 | 0.002 | 0.006 | NA | NA | rs4953361 | 2.5e-5 | 2.5e-5 | NA | NA |
| rs369097672 | 1.8e-5 | 2.8e-7 | 2.3e-5 | 3.3e-6 | rs369097672 | 1.4e-5 | 1.3e-7 | 1.5e-5 | 1.9e-6 | rs369097672 | 5.1e-7 | 4.0e-6 | 5.1e-5 | 2.5e-6 |
| rs3088359 | 1.5e-4 | 2.1e-7 | 2.4e-4 | 5.7e-5 | rs3088359 | 1.3e-4 | 3.0e-7 | 2.5e-4 | 6.2e-5 | rs3088359 | 4.7-7 | 4.2e-8 | 2.0e-5 | 1.1e-6 |
| rs1900592 | 0.008 | 4.6e-7 | 3.5e-5 | 1.1e-5 | rs1900592 | 0.008 | 4.7e-7 | 4.6e-5 | 1.2e-5 | rs1900592 | 1.6e-5 | 7.9e-7 | 8.5e-5 | 6.3e-6 |
| rs1868079 | 0.005 | 2.7e-8 | 1.4e-5 | 2.5e-6 | rs1868079 | 0.003 | 9.9e-9 | 9.3e-6 | 1.3e-6 | rs72618627 | NA | NA | NA | 2.1e-6 |
| rs57720200 | NA | 4.4e-8 | NA | NA | rs57720200 | NA | 9.5e-9 | NA | NA | rs1868079 | 7.2e-5 | 3.7e-6 | 1.8e-5 | 1.7e-6 |
| rs112416191 | 0.005 | 1.4e-8 | 8.6e-6 | 1.4e-6 | rs112416191 | 0.003 | 5.1e-9 | 5.7e-6 | 6.7e-7 | rs7595513 | NA | NA | NA | 1.6e-6 |
| rs116871724 | 0.006 | 8.3e-9 | 6.8e-6 | 1.1e-6 | rs116871724 | 0.003 | 3.0e-9 | 4.5e-6 | 5.4e-7 | rs112416191 | 6.9e-5 | 2.7e-6 | 1.5e-5 | 1.3e-6 |
| rs3814047 | NA | 8.0e-8 | NA | 2.3e-6 | rs3814047 | NA | 1.7e-8 | NA | 9.6e-7 | rs116871724 | 6.5e-5 | 2.1e-6 | 1.3e-5 | 1.1e-6 |
| rs192690066 | NA | 0.001 | NA | NA | rs192690066 | NA | 0.001 | NA | NA | rs192690066 | NA | 0.003 | NA | NA |
| rs116983452 | 0.010 | 5.7e-8 | 1.7e-5 | 2.9e-6 | rs116983452 | 0.006 | 2.3e-8 | 1.3e-5 | 1.6e-6 | rs116983452 | 3.8e-4 | 1.1e-5 | 3.6e-5 | 3.3e-6 |
| rs79542054 | 0.005 | 1.5e-8 | 6.8e-6 | 9.2e-7 | rs79542054 | 0.003 | 5.5e-9 | 4.5e-6 | 4.4e-7 | rs79542054 | 4.7e-5 | 2.6e-6 | 1.8e-5 | 1.4e-6 |
| rs13018477 | NA | 3.8e-8 | NA | 8.0e-7 | rs13018477 | NA | 7.6e-9 | NA | 3.5e-7 |  |  |  |  |  |
| *(Continued)* | | | | | | | | | | | | | | |
|  |  |  |  |  |  |  |  |  |  |  |  |  |  |  |
|  |  |  |  |  |  |  |  |  |  |  |  |  |  |  |
|  |  |  |  |  |  |  |  |  |  |  |  |  |  |  |
|  |  |  |  |  |  |  |  |  |  |  |  |  |  |  |
| **rs79542054** | | | | | **rs192690066** | | | | |  | **rs112416191** | | | |
| rs149594770 | - | 0.024 | - | 0.024 | rs149594770 | 0.001 | 0.002 | 0.002 | 2.7e-4 | rs149594770 | - | 0.023 | - | 0.023 |
| rs375418933 | - | 0.050 | - | - | rs375418933 | 0.002 | 0.003 | 0.004 | 4.9e-4 | rs375418933 | - | 0.044 | - | 0.048 |
| rs1562453 | - | 0.006 | 0.04231 | 0.017 | rs1562453 | 1.3e-4 | 2.2e-4 | 0.003 | 0.001 | rs1562453 | - | 0.006 | 0.036 | 0.015 |
| rs141366568 | - | 0.011 | - | 0.019 | rs7599883 | 3.7e-4 | NA | NA | NA | rs141366568 | - | 0.010 | - | 0.015 |
| rs4953361 | 0.008 | - | NA | NA | rs141366568 | 4.3e-6 | 3.3e-6 | 2.8e-5 | 1.7e-6 | rs4953361 | 0.010 | - | NA | NA |
| rs369097672 | 0.027 | 0.018 | - | 0.026 | rs4953361 | 1.7e-4 | 0.001 | NA | NA | rs369097672 | 0.027 | 0.013 | - | 0.021 |
| rs3088359 | 0.003 | 0.001 | 0.02581 | 0.01 | rs369097672 | 9.4e-6 | 4.3e-6 | 6.1e-5 | 4.4e-6 | rs3088359 | 0.002 | 0.001 | 0.025 | 0.009 |
| **rs13018477** | | | | | rs3088359 | 1.2e-6 | 4.3e-6 | 0.001 | 1.7e-4 | rs1900592 | 0.033 | - | - | - |
| rs149594770 | 1.3e-4 | 4.3e-4 | 0.003 | 2.4e-4 | rs1900592 | 2.8e-5 | 3.5e-6 | 1.7e-4 | 2.3e-5 |  | **rs3814047** | | | |
| rs375418933 | 5.9e-5 | 4.9e-4 | 0.002 | 1.7e-4 | rs72618627 | NA | NA | NA | 0.006 | rs149594770 | 6.5e-5 | 0.001 | 0.005 | 0.001 |
| rs1562453 | 3.8e-5 | 0.001 | 0.004 | 0.001 | rs1868079 | 3.1e-5 | 6.1e-6 | 8.4e-6 | 2.0e-6 | rs375418933 | 5.8e-5 | 0.002 | 0.004 | 0.001 |
| rs7599883 | 1.5e-4 | NA | NA | NA | rs7595513 | NA | NA | NA | 0.007 | rs1562453 | 1.8e-5 | 0.001 | 0.004 | 0.001 |
| rs141366568 | 5.0e-7 | 2.9e-6 | 2.1e-5 | 1.2e-6 | rs57720200 | NA | 0.002 | NA | NA | rs7599883 | 4.7e-5 | NA | NA | NA |
| rs4953361 | 2.1e-5 | 7.2e-5 | NA | NA | rs112416191 | 3.5e-5 | 4.3e-6 | 7.3e-6 | 1.6e-6 | rs141366568 | 1.1e-7 | 1.3e-5 | 4.0e-5 | 2.9e-6 |
| rs369097672 | 3.2e-7 | 3.0e-6 | 6.3e-5 | 2.8e-6 | rs116871724 | 3.7e-5 | 2.8e-6 | 6.9e-6 | 1.6e-6 | rs4953361 | 1.5e-5 | 7.2e-6 | NA | NA |
| rs3088359 | 1.3e-7 | 1.7e-7 | 1.2e-4 | 7.1e-6 | rs3814047 | NA | 0.002 | NA | 0.008 | rs369097672 | 6.9e-8 | 1.2e-5 | 1.2e-4 | 6.6e-6 |
| rs1900592 | 7.0e-6 | 1.1e-6 | 2.3e-4 | 1.5e-5 | rs116983452 | 1.7e-4 | 3.0e-5 | 1.9e-5 | 5.3e-6 | rs3088359 | 1.6e-7 | 9.6e-8 | 5.1e-5 | 2.7e-6 |
| rs72618627 | NA | NA | NA | 3.2e-6 | rs79542054 | 2.4e-5 | 2.6e-6 | 4.0e-6 | 7.5e-7 | rs1900592 | 6.5e-6 | 5.0e-7 | 8.9e-5 | 5.3e-6 |
| rs1868079 | 6.7e-5 | 3.4e-6 | 4.6e-5 | 3.0e-6 | rs13018477 | NA | 0.001 | NA | 0.006 | rs72618627 | NA | NA | NA | 6.4e-6 |
| rs7595513 | NA | NA | NA | 2.9e-6 | **rs116983452** | | | | | rs1868079 | 4.8e-5 | 1.0e-5 | 6.2e-5 | 7.1e-6 |
| rs112416191 | 6.2e-5 | 2.7e-6 | 4.1e-5 | 2.3e-6 | rs149594770 | - | 0.012 | - | 0.019 | rs7595513 | NA | NA | NA | 5.4e-6 |
| rs116871724 | 5.9e-5 | 2.0e-6 | 3.3e-5 | 2.0e-6 | rs375418933 | - | 0.023 | - | 0.043 | rs112416191 | 4.5e-5 | 7.6e-6 | 5.2e-5 | 5.4e-6 |
| rs192690066 | NA | 0.002 | NA | NA | rs1562453 | - | 0.002 | 0.028 | 0.008 | rs116871724 | 4.3e-5 | 6.0e-6 | 4.4e-5 | 4.7e-6 |
| rs116983452 | 2.7e-4 | 6.7e-6 | 5.1e-5 | 3.0e-6 | rs141366568 | 0.033 | 0.003 | 0.047 | 0.011 | rs192690066 | NA | 0.003 | NA | NA |
| **rs116871724** | | | | | rs4953361 | 0.005 | 0.030 | NA | NA | rs116983452 | 2.4e-4 | 2.0e-5 | 8.7e-5 | 9.0e-6 |
| rs149594770 | - | 0.028 | - | 0.023 | rs369097672 | 0.011 | 0.003 | 0.048 | 0.011 | rs79542054 | 2.8e-5 | 8.2e-6 | 5.6e-5 | 5.6e-6 |
| rs375418933 | - | - | - | 0.048 | rs3088359 | 0.001 | 1.1e-4 | 0.011 | 0.003 |  |  |  |  |  |
| rs1562453 | - | 0.006 | 0.036 | 0.015 | rs1900592 | 0.028 | 0.023 | - | 0.047 |  |  |  |  |  |
| rs141366568 | - | 0.010 | - | 0.015 | rs1868079 | - | 0.013 | - | - |  |  |  |  |  |
| rs4953361 | 0.009 | - | NA | NA | rs112416191 | - | 0.006 | - | 0.045 |  |  |  |  |  |
| rs369097672 | 0.027 | 0.013 | - | 0.021 | rs116871724 | - | 0.006 | - | 0.045 |  |  |  |  |  |
| rs3088359 | 0.002 | 0.001 | 0.025 | 0.009 | rs79542054 | - | 0.004 | 0.043 | 0.027 |  |  |  |  |  |
| rs1900592 | 0.032 | - | - | - |  |  |  |  |  |  |  |  |  |  |

The conditional associations of phenotype-associated loci in *EPAS1* and *TMEM247* with 4 traits (Table S9) were tested under a linear model (see \ Methods). The candidate AGV taken as the covariate in each analysis was highlighted with bold font. Candidate AGVs located in *EPAS1* are labeled in red; those in *TMEM247* are labeled in blue. Here listed the significant *p*-values (< 0.05). Candidate AGVs did not show significant association with any trait are not listed in this table. The short line indicates non-significant associations (*p* > 0.05). NA (not available) indicates that the locus is not associated with the phenotype in Table S9 and thus was not tested here.

**Table S17. HAA-related traits collected from literatures.**

| **Traits** | **References** |
| --- | --- |
| Chest circumference and lung volumes (TLC, VC, RV, TV) | (193) |
| FEV1, FVC, FEV1/FVC ratio | (194) |
| Diffusing capacity | (195) |
| A-a gradient | (196) |
| Right ventricular hypertrophy | (197) |
| Pulmonary artery pressures | (198) |
| Hemoglobin concentration | (199) |
| Oxygen content | (198) |
| Maximum heart rate | (200) |
| Cardiac output (ability to increase) | (201) |
| Heart rate (ability to increase) | (201) |
| Right ventricular hypertrophy | (197) |
| Myocardial glucose uptake | (202) |
| Cardiac PCr-to-ATP ratio | (203) |
| Vagal nerve dominance | (198) |
| Psycho-neurological symptoms | (204) |
| Maintenance of cerebral autoregulation | (205) |
| Internal carotid artery flow velocity | (206) |
| Intra-uterine growth retardation | (207) |
| Common iliac blood flow | (198) |
| Utero-placental oxygen delivery | (198) |
| Preeclampsia and gestational hypertension | (207) |
| Premature births and postnatal mortality | (207) |
| Muscle fiber cross-sectional area | (208) |
| Muscle mitochondrial volume density | (209) |
| Capillary density-to-muscle cross fiber ratio | (208) |
| Maximal oxygen consumption-to-mitochondrial volume ratio | (208) |
| Muscle carbohydrate oxidation | (210) |
| Myoglobin concentration | (209) |
| Muscular lipofuscin accumulation | (211) |
| Dominance of type I muscle fibers | (208) |
| Blood lactate levels | (212) |
| V ̇_O2_max | (200) |
| Height and weight | (213) |
| Basal metabolic rate | (214) |
| Weight loss | (215) |
| Blood flow velocity (ability to maximize) | (216) |
| Forearm blood flow | (217) |
| Concentration of bioactive NO products | (217) |
| Concentrations of VEGF-A, IL-8, VEGF-C | (218) |
| Exhaled NO | (219) |
| Rate of alveolar NO transfer | (220) |
| *EGLN1*, *EPAS1* overrepresentation | (220) |
| *(Continued)* | |
| oxygen saturation of hemoglobin | (220) |
| hemoglobin concentrations | (220) |
| Pulmonary O2 transport | (221) |
| Blood O_2_ transport | (221) |
| Erythropoeitic activity | (221) |
| Blood O_2_ affinity | (221) |
| Tissue O_2_ transport | (221) |
| Tissue O_2_ utilization | (221) |
| total metabolism | (221) |
| Weight | (222) |
| Leptin | (223) |
| Growth (children) | (224) |
| Intrauterine growth restriction (IUGR), birth weight | (207, 225-227) |
| Uterine artery blood flow | (228, 229) |
| Thorax growth | (230) |
| Muscle structural, muscle oxidative capacity | (230) |
| Smooth muscle | (231) |
| Arterial oxygen saturation | (232) |
| Metabolic syndrome | (233) |
| Glucose uptake, insulin sensitivity | (234) |
| Obesity | (235, 236) |
| Blood hormone concentrations, endocrine response | (237) |
| High resting ventilation, brisk hypoxic ventilatory sensitivities | (238) |
| Pulmonary arterial hypertension | (239, 240) |
| Nitric oxide | (219, 241) |
| Red cell mass, hemoglobin (Hb) concentration, hematocrit and blood erythropoietin (Epo) levels | (199, 242-248) |
| Chronic mountain sickness (CMS) | (249, 250) |

**Table S18. Parameters used in the simulation.**

| Parameter | Explanation | Value |
| --- | --- | --- |
| *T*_split(AFR-nonAFR)_ | Time of Africans and non-Africans divergence | 51,000 |
| *m*_(AFR-nonAFR)_ | Migration rate between Africans and non-Africans | 1.5 × 10^-5^ |
| *N_1(AFR)_* | *N*_e_ of Africans (constant size) | 14,474 |
| *N_1(nonAFR)_* | *N*_e_ of non-Africans (constant size) | 1,861 |
| *N_1(EUR)_* | *N*_e_ of Europeans (before divergence) | 1,032 |
| *N_1(EAS)_* | *N*_e_ of East Asians (before divergence) | 550 |
| *T*_split(EAS-EUR)_ | Time of East Asians and Europeans divergence | 23,000 |
| *m*_1(EUR-EAS)_ | Migration rate between East Asians and Europeans | 3.11 × 10^-5^ |
| *m*_1(AFR-EUR)_ | Migration rate between Europeans and Africans | 2.5 × 10^-5^ |
| *m*_1(AFR-EAS)_ | Migration rate between East Asians and Africans | 7.8 × 10^-6^ |
| *N_2(AFR)_* | *N*_e_ of Africans (constant size) | 14,474 |
| *N_2(EUR)_* | *N*_e_ of Europeans after exponential size growth | 9,475 |
| *N_2(EAS)_* | *N*_e_ of East Asians after exponential size growth | 8,879 |
| *T_expansion_* | Time of huge expansion of Africans, East Asians and Europeans | 5,115 |
| *m_2_*_(EUR-EAS)_ | Migration rate between East Asians and Europeans | 3.11 × 10^-5^ |
| *m*_2(AFR-EUR)_ | Migration rate between Europeans and Africans | 2.5 × 10^-5^ |
| *m*_2(AFR-EAS)_ | Migration rate between East Asians and Africans | 7.8 × 10^-6^ |
| *N_3(AFR)_* | *N*_e_ of Africans after exponential size growth | 424,000 |
| *N_3(EUR)_* | *N*_e_ of Europeans after exponential size growth | 512,000 |
| *N_3(EAS)_* | *N*_e_ of East Asians after exponential size growth | 1,370,990 |

**Table S19. TIB-specific markers used in the calculation of TMRCA for the haplotypes carrying rs116983452-T.**

| Chromosome | Position (bp) | SNP ID | Ancestral allele | Derived allele | DAF |
| --- | --- | --- | --- | --- | --- |
| 2 | 46657114 | rs75260984 | A | G | 0.763 |
| 2 | 46676881 | rs144204988 | A | G | 0.763 |
| 2 | 46690015 | rs117392422 | T | C | 0.763 |
| 2 | 46691256 | rs76941392 | G | C | 0.763 |
| 2 | 46693330 | rs182127341 | C | A | 0.750 |
| 2 | 46693993 | rs116871724 | T | A | 0.763 |
| 2 | 46705028 | rs141308894 | G | T | 0.763 |
| 2 | 46720473 | rs117677853 | C | T | 0.763 |
| 2 | 46720802 | rs117446572 | G | A | 0.763 |
| 2 | 46722636 | rs80062802 | C | T | 0.763 |
| 2 | 46724022 | rs78788956 | C | A | 0.763 |
| 2 | 46727004 | rs78779826 | G | A | 0.763 |
| 2 | 46728028 | rs143743257 | A | T | 0.763 |
| 2 | 46729845 | rs79117809 | G | C | 0.763 |
| 2 | 46730100 | rs117996002 | C | T | 0.750 |
| 2 | 46732521 | rs74929470 | T | C | 0.737 |
| 2 | 46733738 | rs117128262 | T | C | 0.737 |
| 2 | 46738390 | rs77117514 | C | A | 0.737 |
| 2 | 46752178 | rs117813469 | C | G | 0.724 |
| 2 | 46752292 | rs78082841 | G | A | 0.724 |
| 2 | 46752876 | rs150798075 | G | T | 0.724 |
| 2 | 46754702 | rs138754673 | C | T | 0.724 |
| 2 | 46757984 | rs142764723 | A | G | 0.724 |
| 2 | 46764692 | rs74502090 | T | C | 0.724 |
| 2 | 46768078 | rs75498296 | C | T | 0.724 |
| 2 | 46772997 | rs74869223 | A | G | 0.724 |

**References**

1. Locke, AE, Kahali, B, Berndt, SI*, et al.* Genetic studies of body mass index yield new insights for obesity biology. *Nature*. 2015; **518**(7538): 197-206.

2. Huerta-Sánchez, E, Jin, X, Asan*, et al.* Altitude adaptation in Tibetans caused by introgression of Denisovan-like DNA. *Nature*. 2014; **512**(7513): 194-7.

3. Hackinger, S, Kraaijenbrink, T, Xue, Y*, et al.* Wide distribution and altitude correlation of an archaic high-altitude-adaptive EPAS1 haplotype in the Himalayas. *Human Genetics*. 2016; **135**(4): 393-402.

4. Hanaoka, M, Droma, Y, Basnyat, B*, et al.* Genetic variants in EPAS1 contribute to adaptation to high-altitude hypoxia in Sherpas. *PLoS ONE*. 2012; **7**(12): e50566-e.

5. Xiang, K, Peng, Y, Yang, Z*, et al.* Identification of a Tibetan-specific mutation in the hypoxic gene EGLN1 and its contribution to high-altitude adaptation. *Molecular biology and evolution*. 2013; **30**(8): 1889-98.

6. Lorenzo, FR, Huff, C, Olenchock, B*, et al.* A genetic mechanism for Tibetan high-altitude adaptation. *Nature genetics*. 2014; **46**(9): 951-6.

7. Lawson, DJ, Hellenthal, G, Myers, S*, et al.* Inference of population structure using dense haplotype data. *PLoS genetics*. 2012; **8**(1): e1002453-e.

8. Zhang, C, Lu, Y, Feng, QD*, et al.* Differentiated demographic histories and local adaptations between Sherpas and Tibetans. *Genome Biology*. 2017; **18**.

9. Cho, YS, Hu, L, Hou, HL*, et al.* The tiger genome and comparative analysis with lion and snow leopard genomes. *Nat Commun*. 2013; **4**.

10. Scheinfeldt, LB, Soi, S, Thompson, S*, et al.* Genetic adaptation to high altitude in the Ethiopian highlands. *Genome Biology*. 2012; **13**(1).

11. Wang, BB, Zhang, YB, Zhang, F*, et al.* On the Origin of Tibetans and Their Genetic Basis in Adapting High-Altitude Environments. *Plos One*. 2011; **6**(2).

12. Foll, M, Gaggiotti, OE, Daub, JT*, et al.* Widespread Signals of Convergent Adaptation to High Altitude in Asia and America. *American Journal of Human Genetics*. 2014; **95**(4): 394-407.

13. Ai, HS, Yang, B, Li, J*, et al.* Population history and genomic signatures for high-altitude adaptation in Tibetan pigs. *Bmc Genomics*. 2014; **15**.

14. Horikoshi, M, Beaumont, RN, Day, FR*, et al.* Genome-wide associations for birth weight and correlations with adult disease. *Nature*. 2016; **538**(7624): 248-52.

15. Yi, X, Liang, Y, Huerta-Sanchez, E*, et al.* Sequencing of 50 human exomes reveals adaptation to high altitude. *Science*. 2010; **329**(5987): 75-8.

16. Astle, WJ, Elding, H, Jiang, T*, et al.* The allelic landscape of human blood cell trait variation and links to common complex disease. *Cell*. 2016; **167**(5): 1415-29.

17. Simonson, TS, Yang, YZ, Huff, CD*, et al.* Genetic evidence for high-altitude adaptation in Tibet. *Science*. 2010; **329**(5987): 72-5.

18. Suhre, K, Arnold, M, Bhagwat, AM*, et al.* Connecting genetic risk to disease end points through the human blood plasma proteome. *Nat Commun*. 2017; **8**.

19. Winkler, TW, Justice, AE, Graff, M*, et al.* The Influence of Age and Sex on Genetic Associations with Adult Body Size and Shape: A Large-Scale Genome-Wide Interaction Study. *Plos Genetics*. 2015; **11**(10).

20. Kanai, M, Akiyama, M, Takahashi, A*, et al.* Genetic analysis of quantitative traits in the Japanese population links cell types to complex human diseases. *Nature Genetics*. 2018; **50**(3): 390-+.

21. Sun, BB, Maranville, JC, Peters, JE*, et al.* Genomic atlas of the human plasma proteome. *Nature*. 2018; **558**(7708): 73-9.

22. Warren, HR, Evangelou, E, Cabrera, CP*, et al.* Genome-wide association analysis identifies novel blood pressure loci and offers biological insights into cardiovascular risk. *Nature Genetics*. 2017; **49**(3): 403-15.

23. Graff, M, Scott, RA, Justice, AE*, et al.* Genome-wide physical activity interactions in adiposity. A meta-analysis of 200,452 adults. *Plos Genetics*. 2017; **13**(4).

24. Akiyama, M, Okada, Y, Kanai, M*, et al.* Genome-wide association study identifies 112 new loci for body mass index in the Japanese population. *Nature Genetics*. 2017; **49**(10): 1458-+.

25. Soler Artigas, M, Loth, DW, Wain, LV*, et al.* Genome-wide association and large-scale follow up identifies 16 new loci influencing lung function. *Nature Genetics*. 2011; **43**(11): 1082-90.

26. Hu, H, Petousi, N, Glusman, G*, et al.* Evolutionary history of Tibetans inferred from whole-genome sequencing. *Plos Genet*. 2017; **13**(4).

27. Shin, SY, Fauman, EB, Petersen, AK*, et al.* An atlas of genetic influences on human blood metabolites. *Nature Genetics*. 2014; **46**(6): 543-50.

28. Boger, CA, Gorski, M, McMahon, GM*, et al.* NFAT5 and SLC4A10 Loci Associate with Plasma Osmolality. *J Am Soc Nephrol*. 2017; **28**(8): 2311-21.

29. van der Valk, RJP, Duijts, L, Timpson, NJ*, et al.* Fraction of exhaled nitric oxide values in childhood are associated with 17q11.2-q12 and 17q12-q21 variants. *J Allergy Clin Immun*. 2014; **134**(1): 46-55.

30. Parmar, PG, Taal, HR, Timpson, NJ*, et al.* International Genome-Wide Association Study Consortium Identifies Novel Loci Associated With Blood Pressure in Children and Adolescents. *Circ-Cardiovasc Gene*. 2016; **9**(3): 266-+.

31. Anderson, D, Holt, BJ, Pennell, CE*, et al.* Genome-wide association study of vitamin D levels in children: replication in the Western Australian Pregnancy Cohort (Raine) study. *Genes and Immunity*. 2014; **15**(8): 578-83.

32. Horikoshi, M, Yaghootkar, H, Mook-Kanamori, DO*, et al.* New loci associated with birth weight identify genetic links between intrauterine growth and adult height and metabolism. *Nature Genetics*. 2013; **45**(1): 76-U115.

33. Nagy, R, Boutin, TS, Marten, J*, et al.* Exploration of haplotype research consortium imputation for genome-wide association studies in 20,032 Generation Scotland participants. *Genome Med*. 2017; **9**.

34. Palmer, ND, Goodarzi, MO, Langefeld, CD*, et al.* Genetic variants associated with quantitative glucose homeostasis traits translate to type 2 diabetes in Mexican Americans: the GUARDIAN (Genetics Underlying Diabetes in Hispanics) Consortium. *Diabetes*. 2015; **64**(5): 1853-66.

35. Gharib, SA, Khalyfa, A, Abdelkarim, A*, et al.* Intermittent hypoxia activates temporally coordinated transcriptional programs in visceral adipose tissue. *J Mol Med*. 2012; **90**(4): 435-45.

36. Shungin, D, Winkler, TW, Croteau-Chonka, DC*, et al.* New genetic loci link adipose and insulin biology to body fat distribution. *Nature*. 2015; **518**(7538): 187-96.

37. van Rooij, FJA, Qayyum, R, Smith, AV*, et al.* Genome-wide Trans-ethnic Meta-analysis Identifies Seven Genetic Loci Influencing Erythrocyte Traits and a Role for RBPMS in Erythropoiesis. *American Journal of Human Genetics*. 2017; **100**(1): 51-63.

38. Kamatani, Y, Matsuda, K, Okada, Y*, et al.* Genome-wide association study of hematological and biochemical traits in a Japanese population. *Nature Genetics*. 2010; **42**(3): 210-U25.

39. Harst, PVD, Zhang, W, Leach, IM*, et al.* Seventy-five genetic loci influencing the human red blood cell. *Nature*. 2012; **492**(7429): 369-75.

40. Ganesh, SK, Zakai, NA, van Rooij, FJA*, et al.* Multiple loci influence erythrocyte phenotypes in the CHARGE Consortium. *Nature Genetics*. 2009; **41**(11): 1191-U48.

41. Chen, Z, Tang, H, Qayyum, R*, et al.* Genome-wide association analysis of red blood cell traits in African Americans: the COGENT Network. *Human Molecular Genetics*. 2013; **22**(12): 2529-38.

42. Hodonsky, CJ, Jain, D, Schick, UM*, et al.* Genome- wide association study of red blood cell traits in Hispanics/ Latinos: The Hispanic Community Health Study/ Study of Latinos. *Plos Genetics*. 2017; **13**(4).

43. Ehret, GB, Munroe, PB, Rice, KM*, et al.* Genetic variants in novel pathways influence blood pressure and cardiovascular disease risk. *Nature*. 2011; **478**(7367): 103-9.

44. Wain, LV, Verwoert, GC, O'Reilly, PF*, et al.* Genome-wide association study identifies six new loci influencing pulse pressure and mean arterial pressure. *Nature Genetics*. 2011; **43**(10): 1005-U122.

45. Wain, LV, Vaez, A, Jansen, R*, et al.* Novel Blood Pressure Locus and Gene Discovery Using Genome-Wide Association Study and Expression Data Sets From Blood and the Kidney. *Hypertension*. 2017; **70**(3): E4-+.

46. Kato, N, Loh, M, Takeuchi, F*, et al.* Trans-ancestry genome-wide association study identifies 12 genetic loci influencing blood pressure and implicates a role for DNA methylation. *Nature Genetics*. 2015; **47**(11): 1282-+.

47. Gao, C, Langefeld, CD, Ziegler, JT*, et al.* Genome-wide study of subcutaneous and visceral adipose tissue reveals novel sex-specific adiposity loci in Mexican Americans. *Obesity*. 2018; **26**(1): 202-12.

48. Mahajan, A, Go, MJ, Zhang, WH*, et al.* Genome-wide trans-ancestry meta-analysis provides insight into the genetic architecture of type 2 diabetes susceptibility. *Nature Genetics*. 2014; **46**(3): 234-+.

49. Bonas-Guarch, S, Guindo-Martinez, M, Miguel-Escalada, I*, et al.* Re-analysis of public genetic data reveals a rare X-chromosomal variant associated with type 2 diabetes. *Nat Commun*. 2018; **9**.

50. Wain, LV, Shrine, N, Artigas, MS*, et al.* Genome-wide association analyses for lung function and chronic obstructive pulmonary disease identify new loci and potential druggable targets. *Nature Genetics*. 2017; **49**(3): 416-25.

51. Zhao, W, Rasheed, A, Tikkanen, E*, et al.* Identification of new susceptibility loci for type 2 diabetes and shared etiological pathways with coronary heart disease. *Nature Genetics*. 2017; **49**(10): 1450-+.

52. Comuzzie, AG, Cole, SA, Laston, SL*, et al.* Novel Genetic Loci Identified for the Pathophysiology of Childhood Obesity in the Hispanic Population. *Plos One*. 2012; **7**(12).

53. den Hoed, M, Eijgelsheim, M, Esko, T*, et al.* Identification of heart rate-associated loci and their effects on cardiac conduction and rhythm disorders. *Nature Genetics*. 2013; **45**(6): 621-+.

54. Shao, JM, Raza, MS, Zhuoma, B*, et al.* Evolutionary significance of selected EDAR variants in Tibetan high-altitude adaptations. *Sci China Life Sci*. 2018; **61**(1): 68-78.

55. Fuchs, O, Gorlanova, O, Latzin, P*, et al.* 6q12 and 11p14 variants are associated with postnatal exhaled nitric oxide levels and respiratory symptoms. *J Allergy Clin Immun*. 2017; **140**(4): 1015-23.

56. Yang, J, Jin, ZB, Chen, J*, et al.* Genetic signatures of high-altitude adaptation in Tibetans. *Proceedings of the National Academy of Sciences of the United States of America*. 2017; **114**(16): 4189-94.

57. Peng, Y, Yang, ZH, Zhang, H*, et al.* Genetic Variations in Tibetan Populations and High-Altitude Adaptation at the Himalayas. *Molecular Biology and Evolution*. 2011; **28**(2): 1075-81.

58. Guo, YB, He, YX, Cui, CY*, et al.* GCH1 plays a role in the high-altitude adaptation of Tibetans. *Zool Res*. 2017; **38**(3): 155-62.

59. Franceschini, N, Fox, E, Zhang, Z*, et al.* Genome-wide Association Analysis of Blood-Pressure Traits in African-Ancestry Individuals Reveals Common Associated Genes in African and Non-African Populations. *American Journal of Human Genetics*. 2013; **93**(3): 545-54.

60. Lu, XF, Wang, LY, Lin, X*, et al.* Genome-wide association study in Chinese identifies novel loci for blood pressure and hypertension. *Human Molecular Genetics*. 2015; **24**(3): 865-74.

61. Surendran, P, Drenos, F, Young, R*, et al.* Trans-ancestry meta-analyses identify rare and common variants associated with blood pressure and hypertension. *Nature Genetics*. 2016; **48**(10): 1151-61.

62. Jeong, CW, Alkorta-Aranburu, G, Basnyat, B*, et al.* Admixture facilitates genetic adaptations to high altitude in Tibet. *Nat Commun*. 2014; **5**.

63. Johansson, A, Marroni, F, Hayward, C*, et al.* Linkage and genome-wide association analysis of obesity-related phenotypes: association of weight with the MGAT1 gene. *Obesity*. 2010; **18**(4): 803-8.

64. Bryan, MS, Argos, M, Pierce, B*, et al.* Genome-Wide Association Studies and Heritability Estimates of Body Mass Index Related Phenotypes in Bangladeshi Adults. *Plos One*. 2014; **9**(8).

65. Loth, DW, Artigas, MS, Gharib, SA*, et al.* Genome-wide association analysis identifies six new loci associated with forced vital capacity. *Nature Genetics*. 2014; **46**(7): 669-77.

66. He, J, Kelly, TN, Zhao, Q*, et al.* Genome-Wide Association Study Identifies 8 Novel Loci Associated With Blood Pressure Responses to Interventions in Han Chinese. *Circ-Cardiovasc Gene*. 2013; **6**(6): 598-607.

67. Surakka, I, Isaacs, A, Karssen, LC*, et al.* A Genome-Wide Screen for Interactions Reveals a New Locus on 4p15 Modifying the Effect of Waist-to-Hip Ratio on Total Cholesterol. *Plos Genetics*. 2011; **7**(10).

68. Ong, BA, Li, J, McDonough, JM*, et al.* Gene Network Analysis in a Pediatric Cohort Identifies Novel Lung Function Genes. *Plos One*. 2013; **8**(9).

69. Bouzigon, E, Nadif, R, Thompson, EE*, et al.* A common variant in RAB27A gene is associated with fractional exhaled nitric oxide levels in adults. *Clin Exp Allergy*. 2015; **45**(4): 797-806.

70. Morris, AP, Voight, BF, Teslovich, TM*, et al.* Large-scale association analysis provides insights into the genetic architecture and pathophysiology of type 2 diabetes. *Nature Genetics*. 2012; **44**(9): 981-+.

71. Scott, LJ, Mohlke, KL, Bonnycastle, LL*, et al.* A genome-wide association study of type 2 diabetes in Finns detects multiple susceptibility variants. *Science*. 2007; **316**(5829): 1341-5.

72. Sladek, R, Rocheleau, G, Rung, J*, et al.* A genome-wide association study identifies novel risk loci for type 2 diabetes. *Nature*. 2007; **445**(7130): 881-5.

73. Voight, BF, Scott, LJ, Steinthorsdottir, V*, et al.* Twelve type 2 diabetes susceptibility loci identified through large-scale association analysis. *Nature Genetics*. 2010; **42**(7): 579-U155.

74. Franklin, CS, Aulchenko, YS, Huffman, JE*, et al.* The TCF7L2 Diabetes Risk Variant is Associated with HbA(1C) Levels: a Genome-Wide Association Meta-Analysis. *Annals of Human Genetics*. 2010; **74**: 471-8.

75. Salonen, JT, Uimari, P, Aalto, JM*, et al.* Type 2 diabetes whole-genome association study in four populations: The DiaGen consortium. *American Journal of Human Genetics*. 2007; **81**(2): 338-45.

76. Cook, JP, Morris, AP. Multi-ethnic genome-wide association study identifies novel locus for type 2 diabetes susceptibility. *European Journal of Human Genetics*. 2016; **24**(8): 1175-80.

77. Kulminski, AM, Huang, J, Loika, Y*, et al.* Strong impact of natural-selection-free heterogeneity in genetics of age-related phenotypes. *Aging-Us*. 2018; **10**(3): 492-514.

78. Manning, AK, Hivert, M-F, Scott, RA*, et al.* A genome-wide approach accounting for body mass index identifies genetic variants influencing fasting glycemic traits and insulin resistance. *Nature genetics*. 2012; **44**(6): 659-69.

79. Imamura, M, Takahashi, A, Yamauchi, T*, et al.* Genome-wide association studies in the Japanese population identify seven novel loci for type 2 diabetes. *Nat Commun*. 2016; **7**.

80. Hara, K, Fujita, H, Johnson, TA*, et al.* Genome-wide association study identifies three novel loci for type 2 diabetes. *Human Molecular Genetics*. 2014; **23**(1): 239-46.

81. Rung, J, Cauchi, S, Albrechtsen, A*, et al.* Genetic variant near IRS1 is associated with type 2 diabetes, insulin resistance and hyperinsulinemia. *Nature Genetics*. 2009; **41**(10): 1110-U89.

82. Steinthorsdottir, V, Thorleifsson, G, Reynisdottir, I*, et al.* A variant in CDKAL1 influences insulin response and risk of type 2 diabetes. *Nature Genetics*. 2007; **39**(6): 770-5.

83. Zeggini, E, Scott, LJ, Saxena, R*, et al.* Meta-analysis of genome-wide association data and large-scale replication identifies additional susceptibility loci for type 2 diabetes. *Nature Genetics*. 2008; **40**(5): 638-45.

84. Perry, JRB, Voight, BF, Yengo, L*, et al.* Stratifying Type 2 Diabetes Cases by BMI Identifies Genetic Risk Variants in LAMA1 and Enrichment for Risk Variants in Lean Compared to Obese Cases. *Plos Genetics*. 2012; **8**(5).

85. Ng, MCY, Shriner, D, Chen, BH*, et al.* Meta-Analysis of Genome-Wide Association Studies in African Americans Provides Insights into the Genetic Architecture of Type 2 Diabetes. *Plos Genetics*. 2014; **10**(8).

86. Strawbridge, RJ, Dupuis, J, Prokopenko, I*, et al.* Genome-wide association identifies nine common variants associated with fasting proinsulin levels and provides new insights Into the pathophysiology of type 2 diabetes. *Diabetes*. 2011; **60**(10): 2624-34.

87. Saxena, R, Saleheen, D, Been, LF*, et al.* Genome-wide association study identifies a novel locus contributing to type 2 diabetes susceptibility in Sikhs of Punjabi origin From India. *Diabetes*. 2013; **62**(5): 1746-55.

88. Ghassibe-Sabbagh, M, Haber, M, Salloum, AK*, et al.* T2DM GWAS in the Lebanese population confirms the role of TCF7L2 and CDKAL1 in disease susceptibility. *Scientific Reports*. 2014; **4**.

89. Takeuchi, F, Serizawa, M, Yamamoto, K*, et al.* Confirmation of multiple risk loci and genetic impacts by a genome-wide association study of type 2 diabetes in the Japanese population. *Diabetes*. 2009; **58**(7): 1690-9.

90. Saxena, R, Voight, BF, Lyssenko, V*, et al.* Genome-wide association analysis identifies loci for type 2 diabetes and triglyceride levels. *Science*. 2007; **316**(5829): 1331-6.

91. Zeggini, E, Weedon, MN, Lindgren, CM*, et al.* Replication of genome-wide association signals in UK samples reveals risk loci for type 2 diabetes. *Science*. 2007; **316**(5829): 1336-41.

92. Tabassum, R, Chauhan, G, Dwivedi, OP*, et al.* Genome-wide association study for type 2 diabetes in Indians identifies a new susceptibility locus at 2q21. *Diabetes*. 2013; **62**(3): 977-86.

93. Kho, AN, Hayes, MG, Rasmussen-Torvik, L*, et al.* Use of diverse electronic medical record systems to identify genetic risk for type 2 diabetes within a genome-wide association study. *J Am Med Inform Assn*. 2012; **19**(2): 212-8.

94. Lettre, G, Palmer, CD, Young, T*, et al.* Genome-Wide Association Study of Coronary Heart Disease and Its Risk Factors in 8,090 African Americans: The NHLBI CARe Project. *Plos Genetics*. 2011; **7**(2).

95. Williams, AL, Jacobs, SBR, Moreno-Macias, H*, et al.* Sequence variants in SLC16A11 are a common risk factor for type 2 diabetes in Mexico. *Nature*. 2014; **506**(7486): 97-101.

96. Dupuis, J, Langenberg, C, Prokopenko, I*, et al.* New genetic loci implicated in fasting glucose homeostasis and their impact on type 2 diabetes risk. *Nature Genetics*. 2010; **42**(2): 105-U32.

97. Qi, QB, Stilp, AM, Sofer, T*, et al.* Genetics of type 2 diabetes in US Hispanic/Latino individuals: results from the Hispanic Community Health Study/Study of Latinos (HCHS/SOL). *Diabetes*. 2017; **66**(5): 1419-25.

98. Burton, PR, Clayton, DG, Cardon, LR*, et al.* Genome-wide association study of 14,000 cases of seven common diseases and 3,000 shared controls. *Nature*. 2007; **447**(7145): 661-78.

99. Timpson, NJ, Lindgren, CM, Weedon, MN*, et al.* Adiposity-related heterogeneity in patterns of type 2 diabetes susceptibility observed in genome-wide association data. *Diabetes*. 2009; **58**(2): 505-10.

100. Wheeler, E, Leong, A, Liu, CT*, et al.* Impact of common genetic determinants of Hemoglobin A1c on type 2 diabetes risk and diagnosis in ancestrally diverse populations: A transethnic genome-wide meta-analysis. *Plos Med*. 2017; **14**(9).

101. Simino, J, Shi, G, Bis, JC*, et al.* Gene-Age Interactions in Blood Pressure Regulation: A Large-Scale Investigation with the CHARGE, Global BPgen, and ICBP Consortia. *American Journal of Human Genetics*. 2014; **95**(1): 24-38.

102. Suh, YJ, Lee, CY. Genome-wide association study for genetic variants related with maximal voluntary ventilation reveals two novel genomic signals associated with lung function. *Medicine*. 2017; **96**(44).

103. Eichstaedt, CA, Antao, T, Pagani, L*, et al.* The Andean Adaptive Toolkit to Counteract High Altitude Maladaptation: Genome-Wide and Phenotypic Analysis of the Collas. *Plos One*. 2014; **9**(3).

104. Kim, YK, Kim, Y, Hwang, MY*, et al.* Identification of a genetic variant at 2q12.1 associated with blood pressure in East-Asians by genome-wide scan including gene-environment interactions. *Bmc Med Genet*. 2014; **15**.

105. Eppinga, RN, Hagemeijer, Y, Burgess, S*, et al.* Identification of genomic loci associated with resting heart rate and shared genetic predictors with all-cause mortality. *Nature Genetics*. 2016; **48**(12): 1557-63.

106. Alkorta-Aranburu, G, Beall, CM, Witonsky, DB*, et al.* The Genetic Architecture of Adaptations to High Altitude in Ethiopia. *Plos Genetics*. 2012; **8**(12).

107. Below, JE, Gamazon, ER, Morrison, JV*, et al.* Genome-wide association and meta-analysis in populations from Starr County, Texas, and Mexico City identify type 2 diabetes susceptibility loci and enrichment for expression quantitative trait loci in top signals. *Diabetologia*. 2011; **54**(8): 2047-55.

108. Tsai, FJ, Yang, CF, Chen, CC*, et al.* A Genome-Wide Association Study Identifies Susceptibility Variants for Type 2 Diabetes in Han Chinese. *Plos Genetics*. 2010; **6**(2).

109. Udpa, N, Ronen, R, Zhou, D*, et al.* Whole genome sequencing of Ethiopian highlanders reveals conserved hypoxia tolerance genes. *Genome Biology*. 2014; **15**(2).

110. Bernard, N, Girouard, J, Forest, JC*, et al.* The combination of ApoCIII, hepatic lipase and hormono sensitive lipase gene polymorphisms suggests an association with susceptibility to gestational hypertension. *J Hum Genet*. 2007; **52**(3): 244-54.

111. Lampidonis, AD, Rogdakis, E, Voutsinas, GE*, et al.* The resurgence of Hormone-Sensitive Lipase (HSL) in mammalian lipolysis. *Gene*. 2011; **477**(1-2): 1-11.

112. Weber, GJ, Choe, SE, Dooley, KA*, et al.* Mutant-specific gene programs in the zebrafish. *Blood*. 2005; **106**(2): 521-30.

113. Iqbal, J, Weisenburger, DD, Chowdhury, A*, et al.* Natural killer cell lymphoma shares strikingly similar molecular features with a group of non-hepatosplenic gamma delta T-cell lymphoma and is highly sensitive to a novel aurora kinase A inhibitor in vitro. *Leukemia*. 2011; **25**(2): 348-58.

114. Chen, CH. Platelet-activating factor acetylhydrolase: is it good or bad for you? *Current Opinion in Lipidology*. 2004; **15**(3): 337-41.

115. Arai, H, Koizumi, H, Aoki, J*, et al.* Platelet-activating factor acetylhydrolase (PAF-AH). *Journal of Biochemistry*. 2002; **131**(5): 635-40.

116. Clozel, M, Gray, GA, Breu, V*, et al.* The endothelin ETB receptor mediates both vasodilation and vasoconstriction in vivo. *Biochem Biophys Res Commun*. 1992; **186**(2): 867-73.

117. Mcculloch, KM, Maclean, MR. Endothelin(B) Receptor-Mediated Contraction of Human and Rat Pulmonary Resistance Arteries and the Effect of Pulmonary-Hypertension on Endothelin Responses in the Rat. *J Cardiovasc Pharm*. 1995; **26**: S169-S76.

118. Bigham, AW, Mao, X, Mei, R*, et al.* Identifying positive selection candidate loci for high-altitude adaptation in Andean populations. *Hum Genomics*. 2009; **4**(2): 79-90.

119. Beall, CM, Cavalleri, GL, Deng, LB*, et al.* Natural selection on EPAS1 (HIF2 alpha) associated with low hemoglobin concentration in Tibetan highlanders. *Proceedings of the National Academy of Sciences of the United States of America*. 2010; **107**(25): 11459-64.

120. Huerta-Sanchez, E, DeGiorgio, M, Pagani, L*, et al.* Genetic Signatures Reveal High-Altitude Adaptation in a Set of Ethiopian Populations. *Molecular Biology and Evolution*. 2013; **30**(8): 1877-88.

121. Sanson, M, Ingueneau, C, Vindis, C*, et al.* Oxygen-regulated protein-150 prevents calcium homeostasis deregulation and apoptosis induced by oxidized LDL in vascular cells. *Cell Death and Differentiation*. 2008; **15**(8): 1255-65.

122. Cechowska-Pasko, M, Bankowski, E, Chene, P. The effect of hypoxia on the expression of 150 kDa oxygen-regulated protein (ORP 150) in HeLa cells. *Cellular Physiology and Biochemistry*. 2006; **17**(1-2): 89-96.

123. Gubin, AN, Miller, JL. Human erythroid porphobilinogen deaminase exists in 2 splice variants. *Blood*. 2001; **97**(3): 815-7.

124. Huerta-Sanchez, E, Jin, X, Asan*, et al.* Altitude adaptation in Tibetans caused by introgression of Denisovan-like DNA. *Nature*. 2014; **512**(7513): 194-+.

125. Bigham, A, Bauchet, M, Pinto, D*, et al.* Identifying Signatures of Natural Selection in Tibetan and Andean Populations Using Dense Genome Scan Data. *Plos Genetics*. 2010; **6**(9).

126. Xu, SH, Li, SL, Yang, YJ*, et al.* A Genome-Wide Search for Signals of High-Altitude Adaptation in Tibetans. *Molecular Biology and Evolution*. 2011; **28**(2): 1003-11.

127. Simonson, TS, McClain, DA, Jorde, LB*, et al.* Genetic determinants of Tibetan high-altitude adaptation. *Human Genetics*. 2012; **131**(4): 527-33.

128. Mazzeo, RS, Donovan, D, Fleshner, M*, et al.* Interleukin-6 response to exercise and high-altitude exposure: influence of alpha-adrenergic blockade. *J Appl Physiol*. 2001; **91**(5): 2143-9.

129. Fang, XX, Jiang, XL, Han, XH*, et al.* Neuroprotection of Interleukin-6 Against NMDA-induced Neurotoxicity is Mediated by JAK/STAT3, MAPK/ERK, and PI3K/AKT Signaling Pathways. *Cellular and Molecular Neurobiology*. 2013; **33**(2): 241-51.

130. Zhou, D, Udpa, N, Ronen, R*, et al.* Whole-Genome Sequencing Uncovers the Genetic Basis of Chronic Mountain Sickness in Andean Highlanders. *American Journal of Human Genetics*. 2013; **93**(3): 452-62.

131. Xing, JC, Wuren, TN, Simonson, TS*, et al.* Genomic Analysis of Natural Selection and Phenotypic Variation in High-Altitude Mongolians. *Plos Genetics*. 2013; **9**(7).

132. Gou, X, Wang, Z, Li, N*, et al.* Whole-genome sequencing of six dog breeds from continuous altitudes reveals adaptation to high-altitude hypoxia. *Genome Research*. 2014; **24**(8): 1308-15.

133. Qiu, Q, Zhang, GJ, Ma, T*, et al.* The yak genome and adaptation to life at high altitude. *Nature Genetics*. 2012; **44**(8): 946-+.

134. Zhang, W, Fan, Z, Han, E*, et al.* Hypoxia adaptations in the grey wolf (Canis lupus chanco) from Qinghai-Tibet Plateau. *PLoS Genet*. 2014; **10**(7): e1004466.

135. Wang, MS, Li, Y, Peng, MS*, et al.* Genomic Analyses Reveal Potential Independent Adaptation to High Altitude in Tibetan Chickens. *Molecular Biology and Evolution*. 2015; **32**(7): 1880-9.

136. Ge, RL, Cai, QL, Shen, YY*, et al.* Draft genome sequence of the Tibetan antelope. *Nat Commun*. 2013; **4**.

137. Li, MZ, Tian, SL, Jin, L*, et al.* Genomic analyses identify distinct patterns of selection in domesticated pigs and Tibetan wild boars. *Nature Genetics*. 2013; **45**(12): 1431-U180.

138. Zhou, D, Udpa, N, Gersten, M*, et al.* Experimental selection of hypoxia-tolerant Drosophila melanogaster. *Proceedings of the National Academy of Sciences of the United States of America*. 2011; **108**(6): 2349-54.

139. Wei, CH, Wang, HH, Liu, G*, et al.* Genome-wide analysis reveals adaptation to high altitudes in Tibetan sheep. *Scientific Reports*. 2016; **6**.

140. Shi, Y, Hu, YS, Wang, J*, et al.* Genetic diversities of MT-ND1 and MT-ND2 genes are associated with high-altitude adaptation in yak. *Mitochondrial DNA A*. 2018; **29**(3): 485-94.

141. Wang, J, Shi, Y, Elzo, MA*, et al.* Genetic diversity of ATP8 and ATP6 genes is associated with high-altitude adaptation in yak. *Mitochondrial DNA A*. 2018; **29**(3): 385-93.

142. Zhang, YW, Gou, WY, Ma, J*, et al.* Genome methylation and regulatory functions for hypoxic adaptation in Tibetan chicken embryos. *Peerj*. 2017; **5**.

143. Wang, GD, Zhang, BL, Zhou, WW*, et al.* Selection and environmental adaptation along a path to speciation in the Tibetan frog Nanorana parkeri. *Proceedings of the National Academy of Sciences of the United States of America*. 2018; **115**(22): E5056-E65.

144. Li, JT, Gao, YD, Xie, L*, et al.* Comparative genomic investigation of high-elevation adaptation in ectothermic snakes. *Proceedings of the National Academy of Sciences of the United States of America*. 2018; **115**(33): 8406-11.

145. Lian, T, Li, DY, Tan, XX*, et al.* Genetic diversity and natural selection in wild fruit flies revealed by whole-genome resequencing. *Genomics*. 2018; **110**(5): 304-9.

146. Zheng, WS, He, YX, Cui, CY*, et al.* EP300 contributes to high-altitude adaptation in Tibetans by regulating nitric oxide production. *Zool Res*. 2017; **38**(3): 163-70.

147. Yang, DY, Peng, Y, Ouzhuluobu*, et al.* HMOX2 Functions as a Modifier Gene for High-Altitude Adaptation in Tibetans. *Human Mutation*. 2016; **37**(2): 216-23.

148. Bigham, AW. Genetics of human origin and evolution: high-altitude adaptations. *Current Opinion in Genetics & Development*. 2016; **41**: 8-13.

149. Jha, AR, Zhou, D, Brown, CD*, et al.* Shared Genetic Signals of Hypoxia Adaptation in Drosophila and in High-Altitude Human Populations. *Molecular Biology and Evolution*. 2016; **33**(2): 501-17.

150. Arciero, E, Kraaijenbrink, T, Asan*, et al.* Demographic History and Genetic Adaptation in the Himalayan Region Inferred from Genome-Wide SNP Genotypes of 49 Populations. *Molecular Biology and Evolution*. 2018; **35**(8): 1916-33.

151. Spivak, JL. The blood in systemic disorders. *Lancet*. 2000; **355**(9216): 1707-12.

152. Ebara, S, Adachi, S, Takenaka, S*, et al.* Hypoxia-induced megaloblastosis in vitamin B12-deficient rats. *Br J Nutr*. 2003; **89**(4): 441-4.

153. Yu, L, Chen, Y, Wang, W*, et al.* Multi-vitamin B supplementation reverses hypoxia-induced tau hyperphosphorylation and improves memory function in adult mice. *J Alzheimers Dis*. 2016; **54**(1): 297-306.

154. Chalupsky, K, Kracun, D, Kanchev, I*, et al.* Folic acid promotes recycling of tetrahydrobiopterin and protects against hypoxia-induced pulmonary hypertension by recoupling endothelial nitric oxide synthase. *Antioxid Redox Signal*. 2015; **23**(14): 1076-91.

155. Cheng, F, Lan, J, Xia, W*, et al.* Folic acid attenuates vascular endothelial cell injury caused by hypoxia via the inhibition of ERK1/2/NOX4/ROS pathway. *Cell Biochem Biophys*. 2016; **74**(2): 205-11.

156. Huang, XY, He, ZY, Jiang, XW*, et al.* Folic Acid Represses Hypoxia-Induced Inflammation in THP-1 Cells through Inhibition of the PI3K/Akt/HIF-1 alpha Pathway. *Plos One*. 2016; **11**(3).

157. Raz, S, Sheban, D, Gonen, N*, et al.* Severe hypoxia induces complete antifolate resistance in carcinoma cells due to cell cycle arrest. *Cell Death Dis*. 2014; **5**.

158. Yoshida, Y, Takahashi, K, Okita, K*, et al.* Hypoxia enhances the generation of induced pluripotent stem cells. *Cell Stem Cell*. 2009; **5**(3): 237-41.

159. Wang, GL, Jiang, BH, Semenza, GL. Effect of Protein-Kinase and Phosphatase Inhibitors on Expression of Hypoxia-Inducible Factor-1. *Biochem Bioph Res Co*. 1995; **216**(2): 669-75.

160. Hansensmith, FM, Blackwell, LH, Joswiak, GR. Expression of Muscle Capillary Alkaline-Phosphatase Is Affected by Hypoxia. *J Appl Physiol*. 1992; **73**(2): 776-80.

161. Ren, HY, Cao, Y, Zhao, QJ*, et al.* Proliferation and differentiation of bone marrow stromal cells under hypoxic conditions. *Biochem Bioph Res Co*. 2006; **347**(1): 12-21.

162. Yu, CH, Kastin, AJ, Ding, YM*, et al.* Gamma glutamyl transpeptidase is a dynamic indicator of endothelial response to stroke. *Exp Neurol*. 2007; **203**(1): 116-22.

163. Stastny, F, Tomasova, H, Trojan, S. Effects of Short-Term and Prolonged Aerogenic Hypoxia on Gamma-Glutamyl-Transferase Transpeptidase Activity in the Brain, Liver, and Biological-Fluids of Young-Rats. *Neurochem Res*. 1985; **10**(6): 819-28.

164. JoyceBrady, M, Oakes, SM, Wuthrich, D*, et al.* Three alternative promoters of the rat gamma-glutamyl transferase gene are active in developing lung and are differentially regulated by oxygen after birth. *J Clin Invest*. 1996; **97**(7): 1774-9.

165. Gude, F, Rey-Garcia, J, Fernandez-Merino, C*, et al.* Serum levels of gamma-glutamyl transferase are associated with markers of nocturnal hypoxemia in a general adult population. *Clin Chim Acta*. 2009; **407**(1-2): 67-71.

166. Elsayed, NM, Nakashima, JM, Postlethwait, EM. Measurement of Uric-Acid as a Marker of Oxygen-Tension in the Lung. *Arch Biochem Biophys*. 1993; **302**(1): 228-32.

167. Saito, H, Nishimura, M, Shibuya, E*, et al.* Tissue hypoxia in sleep apnea syndrome assessed by uric acid and adenosine. *Chest*. 2002; **122**(5): 1686-94.

168. Leyva, F, Anker, S, Swan, JW*, et al.* Serum uric acid as an index of impaired oxidative metabolism in chronic heart failure. *Eur Heart J*. 1997; **18**(5): 858-65.

169. Marro, PJ, Mcgowan, JE, Razdan, B*, et al.* Effect of Allopurinol on Uric-Acid Levels and Brain-Cell Membrane Na+,K+-Atpase Activity during Hypoxia in Newborn Piglets. *Brain Res*. 1994; **650**(1): 9-15.

170. Julian, RJ, Summers, J, Wilson, JB. Right Ventricular Failure and Ascites in Broiler-Chickens Caused by Phosphorus-Deficient Diets. *Avian Dis*. 1986; **30**(3): 453-9.

171. Kusuoka, H, Weisfeldt, ML, Zweier, JL*, et al.* Mechanism of Early Contractile Failure during Hypoxia in Intact Ferret Heart - Evidence for Modulation of Maximal Ca-2+-Activated Force by Inorganic-Phosphate. *Circ Res*. 1986; **59**(3): 270-82.

172. Dietrich, HH, Ellsworth, ML, Sprague, RS*, et al.* Red blood cell regulation of microvascular tone through adenosine triphosphate. *Am J Physiol-Heart C*. 2000; **278**(4): H1294-H8.

173. Bergfeld, GR, Forrester, T. Release of Atp from Human Erythrocytes in Response to a Brief Period of Hypoxia and Hypercapnia. *Cardiovasc Res*. 1992; **26**(1): 40-7.

174. Blaise, S, Alberto, JM, Nedelec, E*, et al.* Mild neonatal hypoxia exacerbates the effects of vitamin-deficient diet on homocysteine metabolism in rats. *Pediatr Res*. 2005; **57**(6): 777-82.

175. Blaise, SA, Nedelec, E, Alberto, JM*, et al.* Short hypoxia could attenuate the adverse effects of hyperhomocysteinemia on the developing rat brain by inducing neurogenesis. *Exp Neurol*. 2009; **216**(1): 231-8.

176. Li, LJ, Huang, XH, Jia, ZH*, et al.* Evaluation of the impacts of homocysteine and hypoxia on vascular endothelial function based on the profiling of neuro-endocrine-immunity network in rats. *Pharmacol Res*. 2009; **60**(4): 277-83.

177. Roybal, CN, Yang, SJ, Sun, CW*, et al.* Homocysteine increases the expression of vascular endothelial growth factor by a mechanism involving endoplasmic reticulum stress and transcription factor ATF4. *J Biol Chem*. 2004; **279**(15): 14844-52.

178. Parthasarathi, K, Lipowsky, HH. Capillary recruitment in response to tissue hypoxia and its dependence on red blood cell deformability. *Am J Physiol-Heart C*. 1999; **277**(6): H2145-H57.

179. Phelan, JP, Ahn, MO, Korst, LM*, et al.* Nucleated Red-Blood-Cells - a Marker for Fetal Asphyxia. *Am J Obstet Gynecol*. 1995; **173**(5): 1380-4.

180. Bracci, R, Perrone, S, Buonocore, G. Red blood cell involvement in fetal/neonatal hypoxia. *Biol Neonate*. 2001; **79**(3-4): 210-2.

181. Phelan, JP, Kirkendall, C, Korst, LM*, et al.* Nucleated red blood cell and platelet counts in asphyxiated neonates sufficient to result in permanent neurologic impairment. *J Matern-Fetal Neo M*. 2007; **20**(5): 377-80.

182. Storz, JF, Moriyama, H. Mechanisms of hemoglobin adaptation to high altitude hypoxia. *High Alt Med Biol*. 2008; **9**(2): 148-57.

183. Allen, BW, Piantadosi, CA. How do red blood cells cause hypoxic vasodilation? The SNO-hemoglobin paradigm. *Am J Physiol-Heart C*. 2006; **291**(4): H1507-H12.

184. Beall, CM, Reichsman, AB. Hemoglobin Levels in a Himalayan High-Altitude Population. *Am J Phys Anthropol*. 1984; **63**(3): 301-6.

185. Heinicke, K, Prommer, N, Cajigal, J*, et al.* Long-term exposure to intermittent hypoxia results in increased hemoglobin mass, reduced plasma volume, and elevated erythropoietin plasma levels in man. *Eur J Appl Physiol*. 2003; **88**(6): 535-43.

186. Deem, S, Swenson, ER, Alberts, MK*, et al.* Red-blood-cell augmentation of hypoxic pulmonary vasoconstriction - Hematocrit dependence and the importance of nitric oxide. *Am J Resp Crit Care*. 1998; **157**(4): 1181-6.

187. Voelkel, NF. Mechanisms of Hypoxic Pulmonary Vasoconstriction. *Am Rev Respir Dis*. 1986; **133**(6): 1186-95.

188. Burton, RR, Smith, AH, Carlisle, JC*, et al.* Role of Hematocrit, Heart Mass, and High-Altitude Exposure in Acute Hypoxia Tolerance. *J Appl Physiol*. 1969; **27**(1): 49-&.

189. Ulatowski, JA, Bucci, E, Razynska, A*, et al.* Cerebral blood flow during hypoxic hypoxia with plasma-based hemoglobin at reduced hematocrit. *Am J Physiol-Heart C*. 1998; **274**(6): H1933-H42.

190. Beall, CM, Cavalleri, GL, Deng, L*, et al.* Natural selection on EPAS1 (HIF2alpha) associated with low hemoglobin concentration in Tibetan highlanders. *Proc Natl Acad Sci U S A*. 2010; **107**(25): 11459-64.

191. Jeong, C, Witonsky, DB, Basnyat, B*, et al.* Detecting past and ongoing natural selection among ethnically Tibetan women at high altitude in Nepal. *Plos Genet*. 2018; **14**(9): e1007650.

192. Peng, Y, Cui, CY, He, YX*, et al.* Down-Regulation of EPAS1 Transcription and Genetic Adaptation of Tibetans to High-Altitude Hypoxia. *Molecular Biology and Evolution*. 2017; **34**(4): 818-30.

193. Chen, QH, Ge, RL, Wang, XZ*, et al.* Exercise performance of Tibetan and Han adolescents at altitudes of 3,417 and 4,300 m. *J Appl Physiol*. 1997; **83**(2): 661-7.

194. Havryk, AP, Gilbert, M, Burgess, KR. Spirometry values in Himalayan high altitude residents (Sherpas). *Resp Physiol Neurobi*. 2002; **132**(2): 223-32.

195. Droma, T, McCullough, RG, McCullough, RE*, et al.* Increased vital and total lung capacities in Tibetan compared to Han residents of Lhasa (3,658 m). *Am J Phys Anthropol*. 1991; **86**(3): 341-51.

196. Brutsaert, TD. Do high-altitude natives have enhanced exercise performance at altitude? *Appl Physiol Nutr Me*. 2008; **33**(3): 582-92.

197. Halperin, BD, Sun, SF, Zhuang, JG*, et al.* ECG observations in Tibetan and han residents of Lhasa. *J Electrocardiol*. 1998; **31**(3): 237-43.

198. Gilbert-Kawai, ET, Milledge, JS, Grocott, MPW*, et al.* King of the mountains: Tibetan and Sherpa physiological adaptations for life at high altitude. *Physiology*. 2014; **29**(6): 388-402.

199. Wu, TY, Wang, XQ, Wei, CY*, et al.* Hemoglobin levels in Qinghai-Tibet: different effects of gender for Tibetans vs. Han. *J Appl Physiol*. 2005; **98**(2): 598-604.

200. Pugh, LGCE. Excerpts from: Physiological and medical aspects of the Himalayan Scientific and Mountaineering Expedition, 1960-61. *Wild Environ Med*. 2002; **13**(1): 57-.

201. Moore, LG, Niermeyer, S, Zamudio, S. Human adaptation to high altitude: Regional and life-cycle perspectives. *Yearb Phys Anthropol*. 1998; **41**: 25-64.

202. Holden, JE, Stone, CK, Clark, CM*, et al.* Enhanced Cardiac Metabolism of Plasma-Glucose in High-Altitude Natives - Adaptation against Chronic Hypoxia. *J Appl Physiol*. 1995; **79**(1): 222-8.

203. Hochachka, PW, Clark, CM, Holden, JE*, et al.* P-31 magnetic resonance spectroscopy of the Sherpa heart: A phosphocreatine adenosine triphosphate signature of metabolic defense against hypobaric hypoxia. *Proceedings of the National Academy of Sciences of the United States of America*. 1996; **93**(3): 1215-20.

204. Garrido, E, Segura, R, Capdevila, A*, et al.* Are Himalayan Sherpas better protected against brain damage associated with extreme altitude climbs? *Clin Sci*. 1996; **90**(1): 81-5.

205. Jansen, GFA, Krins, A, Basnyat, B*, et al.* Cerebral autoregulation in subjects adapted and not adapted to high altitude. *Stroke*. 2000; **31**(10): 2314-8.

206. Huang, SY, Sun, SF, Droma, T*, et al.* Internal Carotid Arterial Flow Velocity during Exercise in Tibetan and Han Residents of Lhasa (3,658-M). *J Appl Physiol*. 1992; **73**(6): 2638-42.

207. Zamudio, S, Droma, T, Norkyel, KY*, et al.* Protection from Intrauterine Growth-Retardation in Tibetans at High-Altitude. *Am J Phys Anthropol*. 1993; **91**(2): 215-24.

208. Kayser, B, Hoppeler, H, Claassen, H*, et al.* Muscle Structure and Performance Capacity of Himalayan Sherpas. *J Appl Physiol*. 1991; **70**(5): 1938-42.

209. Marconi, C, Marzorati, M, Cerretelli, P. Work capacity of permanent residents of high altitude. *High Alt Med Biol*. 2006; **7**(2): 105-15.

210. Hochachka, PW, Stanley, C, Mckenzie, DC*, et al.* Enzyme Mechanisms for Pyruvate-to-Lactate Flux Attenuation - a Study of Sherpas, Quechuas, and Hummingbirds. *Int J Sports Med*. 1992; **13**: S119-S22.

211. Gelfi, C, De Palma, S, Ripamonti, M*, et al.* New aspects of altitude adaptation in Tibetans: a proteomic approach. *Faseb J*. 2004; **18**(1): 612-+.

212. West, JB. Lactate during Exercise at Extreme Altitude. *Federation Proceedings*. 1986; **45**(13): 2953-7.

213. Weitz, CA, Garruto, RM, Chin, CT*, et al.* Growth of Qinghai Tibetans living at three different high altitudes. *Am J Phys Anthropol*. 2000; **111**(1): 69-88.

214. Gill, MB, Pugh, LGC. Basal Metabolism + Respiration in Men Living at 5,800 M (19,000 Ft). *J Appl Physiol*. 1964; **19**(5): 949-&.

215. Ward, M. High Altitude Deterioration. *Proc R Soc Ser B-Bio*. 1954; **143**(910): 40-2.

216. Schneider, A, Greene, RE, Keyl, C*, et al.* Peripheral arterial vascular function at altitude: sea-level natives versus Himalayan high-altitude natives. *J Hypertens*. 2001; **19**(2): 213-22.

217. Erzurum, SC, Ghosh, S, Janocha, AJ*, et al.* Higher blood flow and circulating NO products offset high-altitude hypoxia among Tibetans. *Proceedings of the National Academy of Sciences of the United States of America*. 2007; **104**(45): 17593-8.

218. Patitucci, M, Lugrin, D, Pages, G. Angiogenic/lymphangiogenic factors and adaptation to extreme altitudes during an expedition to Mount Everest. *Acta Physiol*. 2009; **196**(2): 259-65.

219. Beall, CM, Laskowski, D, Strohl, KP*, et al.* Pulmonary nitric oxide in mountain dwellers. *Nature*. 2001; **414**(6862): 411-2.

220. Scheinfeldt, LB, Tishkoff, SA. Living the high life: high-altitude adaptation. *Genome Biology*. 2010; **11**(9).

221. Storz, JF, Scott, GR, Cheviron, ZA. Phenotypic plasticity and genetic adaptation to high-altitude hypoxia in vertebrates. *Journal of Experimental Biology*. 2010; **213**(24): 4125-36.

222. Lippl, FJ, Neubauer, S, Schipfer, S*, et al.* Hypobaric hypoxia causes body weight reduction in obese subjects. *Obesity*. 2010; **18**(4): 675-81.

223. Xi, HJ, Zhang, LP, Guol, ZY*, et al.* Serum Leptin Concentration and Its Effect on Puberty in Naqu Tibetan Adolescents. *J Physiol Anthropol*. 2011; **30**(3): 111-7.

224. Argnani, L, Cogo, A, Gualdi-Russo, E. Growth and nutritional status of Tibetan children at high altitude. *Coll Antropol*. 2008; **32**(3): 807-12.

225. Julian, CG, Wilson, MJ, Moore, LG. Evolutionary Adaptation to High Altitude: A View From In Utero. *American Journal of Human Biology*. 2009; **21**(5): 614-22.

226. Moore, LG, Shriver, M, Bemis, L*, et al.* Maternal adaptation to high-altitude pregnancy: an experiment of nature--a review. *Placenta*. 2004; **25 Suppl A**: S60-71.

227. Moore, LG, Young, D, McCullough, RE*, et al.* Tibetan protection from intrauterine growth restriction (IUGR) and reproductive loss at high altitude. *American Journal of Human Biology*. 2001; **13**(5): 635-44.

228. Moore, LG, Zamudio, S, Zhuang, JG*, et al.* Oxygen transport in Tibetan women during pregnancy at 3,658 m. *Am J Phys Anthropol*. 2001; **114**(1): 42-53.

229. Chen, D, Zhou, X, Zhu, Y*, et al.* Comparison study on uterine and umbilical artery blood flow during pregnancy at high altitude and at low altitude. *Zhonghua Fu Chan Ke Za Zhi*. 2002; **37**(2): 69-71.

230. Weitz, CA, Garruto, RM, Chin, CT*, et al.* Morphological growth and thorax dimensions among Tibetan compared to Han children, adolescents and young adults born and raised at high altitude. *Annals of Human Biology*. 2004; **31**(3): 292-310.

231. Gupta, ML, Rao, KS, Anand, IS*, et al.* Lack of Smooth-Muscle in the Small Pulmonary-Arteries of the Native Ladakhi - Is the Himalayan Highlander Adapted. *Am Rev Respir Dis*. 1992; **145**(5): 1201-4.

232. Niermeyer, S, Yang, P, Shanmina*, et al.* Arterial Oxygen-Saturation in Tibetan and Han Infants Born in Lhasa, Tibet. *New Engl J Med*. 1995; **333**(19): 1248-52.

233. Baracco, R, Mohanna, S, Seclen, S. A comparison of the prevalence of metabolic syndrome and its components in high and low altitude populations in peru. *Metab Syndr Relat Disord*. 2007; **5**(1): 55-62.

234. Gamboa, JL, Garcia-Cazarin, ML, Andrade, FH. Chronic hypoxia increases insulin-stimulated glucose uptake in mouse soleus muscle. *Am J Physiol-Reg I*. 2011; **300**(1): R85-R91.

235. Khalid, MEM, Ali, ME. Relationship of Body-Weight to Altitude in Saudi-Arabia. *Ann Saudi Med*. 1994; **14**(4): 300-3.

236. Sherpa, LY, Deji, Stigum, H*, et al.* Obesity in Tibetans Aged 30-70 Living at Different Altitudes under the North and South Faces of Mt. Everest. *Int J Env Res Pub He*. 2010; **7**(4): 1670-80.

237. Barnholt, KE, Hoffman, AR, Rock, PB*, et al.* Endocrine responses to acute and chronic high-altitude exposure (4,300 meters): modulating effects of caloric restriction. *Am J Physiol-Endoc M*. 2006; **290**(6): E1078-E88.

238. Hackett, PH, Reeves, JT, Reeves, CD*, et al.* Control of Breathing in Sherpas at Low and High-Altitude. *J Appl Physiol*. 1980; **49**(3): 374-9.

239. Motley, HL, Cournand, A, Werko, L*, et al.* The Influence of Short Periods of Induced Acute Anoxia Upon Pulmonary Artery Pressures in Man. *Am J Physiol*. 1947; **150**(2): 315-20.

240. Groves, BM, Droma, T, Sutton, JR*, et al.* Minimal Hypoxic Pulmonary-Hypertension in Normal Tibetans at 3,658-M. *J Appl Physiol*. 1993; **74**(1): 312-8.

241. Hoit, BD, Dalton, ND, Erzurum, SC*, et al.* Nitric oxide and cardiopulmonary hemodynamics in Tibetan highlanders. *J Appl Physiol*. 2005; **99**(5): 1796-801.

242. Richalet, JP, Souberbielle, JC, Antezana, AM*, et al.* Control of Erythropoiesis in Humans during Prolonged Exposure to the Altitude of 6,542-M. *Am J Physiol*. 1994; **266**(3): R756-R64.

243. Beall, CM. Two routes to functional adaptation: Tibetan and Andean high-altitude natives. *Proc Natl Acad Sci U S A*. 2007; **104 Suppl 1**: 8655-60.

244. Beall, CM, Brittenham, GM, Strohl, KP*, et al.* Hemoglobin concentration of high-altitude Tibetans and Bolivian Aymara. *Am J Phys Anthropol*. 1998; **106**(3): 385-400.

245. Garruto, RM, Chin, CT, Weitz, CA*, et al.* Hematological differences during growth among Tibetans and Han Chinese born and raised at high altitude in Qinghai, China. *Am J Phys Anthropol*. 2003; **122**(2): 171-83.

246. Winslow, RM, Chapman, KW, Gibson, CC*, et al.* Different Hematologic Responses to Hypoxia in Sherpas and Quechua Indians. *J Appl Physiol*. 1989; **66**(4): 1561-9.

247. Wu, TY, Kayser, B. High altitude adaptation in Tibetans. *High Alt Med Biol*. 2006; **7**(3): 193-208.

248. Tannheimer, M, Fusch, C, Boning, D*, et al.* Changes of hematocrit and hemoglobin concentration in the cold Himalayan environment in dependence on total body fluid. *Sleep Breath*. 2010; **14**(3): 193-9.

249. Moore, LG, Armaza, F, Villena, M*, et al.* Comparative aspects of high-altitude adaptation in human populations. *Adv Exp Med Biol*. 2000; **475**: 45-62.

250. Monge, CC, Arregui, A, Leon-Velarde, F. Pathophysiology and epidemiology of chronic mountain sickness. *Int J Sports Med*. 1992; **13 Suppl 1**: S79-81.
